# Protein Structural Modeling and Transport Thermodynamics Reveal That Plant Cation–Chloride Cotransporters Mediate Potassium–Chloride Symport

**DOI:** 10.3390/ijms252312955

**Published:** 2024-12-02

**Authors:** Sam W. Henderson, Saeed Nourmohammadi, Maria Hrmova

**Affiliations:** School of Agriculture, Food and Wine, Waite Research Institute, Faculty of Sciences, Engineering and Technology, University of Adelaide, Waite Campus Precinct, Glen Osmond, Adelaide, SA 5064, Australia; sam.henderson@adelaide.edu.au (S.W.H.); saeed.nourmohammadi@adelaide.edu.au (S.N.)

**Keywords:** endomembrane system, KCC, net ion flux, NKCC, structure–function

## Abstract

Plant cation–chloride cotransporters (CCCs) are proposed to be Na^+^-K^+^-2Cl^−^ transporting membrane proteins, although evolutionarily, they associate more closely with K^+^-Cl^−^ cotransporters (KCCs). Here, we investigated grapevine (*Vitis vinifera* L.) VvCCC using 3D protein modeling, bioinformatics, and electrophysiology with a heterologously expressed protein. The 3D protein modeling revealed that the signatures of ion binding sites in plant CCCs resembled those of animal KCCs, which was supported by phylogenomic analyses and ancestral sequence reconstruction. The conserved features of plant CCCs and animal KCCs included predicted K^+^ and Cl^−^-binding sites and the absence of a Na^+^-binding site. Measurements with *VvCCC*-injected *Xenopus laevis* oocytes with VvCCC localizing to plasma membranes indicated that the oocytes had depleted intracellular Cl^−^ and net ^86^Rb fluxes, which agreed with thermodynamic predictions for KCC cotransport. The ^86^Rb uptake by *VvCCC*-injected oocytes was Cl^−^-dependent, did not require external Na^+^, and was partially inhibited by the non-specific CCC-blocker bumetanide, implying that these properties are typical of KCC transporters. A loop diuretic-insensitive Na^+^ conductance in *VvCCC*-injected oocytes may account for earlier observations of Na^+^ uptake by plant CCC proteins expressed in oocytes. Our data suggest plant CCC membrane proteins are likely to function as K^+^-Cl^−^ cotransporters, which opens the avenues to define their biophysical properties and roles in plant physiology.

## 1. Introduction

Cation–chloride cotransporters (CCCs), classified in the solute carrier family 12 (SLC12) [1], mediate electroneutral secondary transport of monovalent Cl^−^ and Na^+^ or K^+^ across membranes. Animal cells have three types of CCCs: NKCC proteins that simultaneously transport Na^+^, K^+^, and Cl^−^; NCC proteins that cotransport Na^+^ and Cl^−^; and KCC proteins that cotransport K^+^ and Cl^−^ [2]. The biological roles of animal NKCC and NCC proteins reside in re-absorptive and secretory transepithelial transport, depending on their epithelial tissues’ localization. Unlike animals, land plants encode fewer CCC proteins in their genomes and plant CCC substrate stoichiometry is unknown [3].

Thermodynamic properties of animal NKCCs and KCCs were validated experimentally [4]. Most animal CCC proteins reside on plasma membranes where ion gradients established by the Na^+^/K^+^-ATPase determine transport direction and physiological function. It was predicted that CCC proteins are accommodated in highly curved sphere-shaped membranes bent toward the cytoplasmic sides [5]. Here, the KCC proteins mediate a net efflux of K^+^-Cl^−^ down the chemical concentration gradient for K^+^, which is vital for inhibitory neurotransmission [6]. NCC and NKCC proteins mediate the net influx of Na^+^, K^+^, and 2Cl^−^, driven by chemical gradients for Na^+^ and Cl^−^, which is important for solute reabsorption. Conversely, the plant CCC proteins reside in the Golgi, *trans*-Golgi network (TGN), and early endosome (EE) membranes [3,7,8]. Their kinetics and thermodynamic properties are debated [9], and even water cotransport against a thermodynamic gradient has been reported [10]. However, in-depth structural and biophysical studies of plant CCCs, which would define transport properties, are lacking.

Plant CCCs are considered to be Na^+^-K^+^-2Cl^−^ symporters [3,10,11,12,13]. This is because *Xenopus laevis* oocytes expressing AtCCC from *Arabidopsis thaliana* showed greater ^22^Na, ^86^Rb, and ^36^Cl radiotracer uptake than water-injected control oocytes [12]; these findings were validated with the orthologous VvCCC protein from grapevine (*Vitis vinifera* L.) expressed in oocytes [3]. The radiotracer fluxes of *X. laevis* oocytes-expressed AtCCC mediated the coordinated symport of K^+^, Na^+^, and Cl^−^ ions under both isotonic and hypotonic conditions, and these activities were inhibited by 100 µM bumetanide, a loop diuretic generic drug and a vertebrate Na^+^-K^+^-Cl^−^ cotransporter inhibitor [14]. Further, cotransport in AtCCC was pH-dependent, although it did not behave as an H^+^-dependent cotransporter. These data indicated that in *Xenopus* oocytes, cotransport mediated by AtCCC required the simultaneous presence of all three ions [3,12]. However, bumetanide also inhibits KCC proteins, but at higher concentrations [4]. Expression of *Oryza sativa* OsCCC1 in yeast (*Saccharomyces cerevisiae*) led to greater K^+^, Cl^−^, and Na^+^ uptake [11], further suggesting that plant CCCs could facilitate the cotransport of the three ions.

Phylogenetically, however, plant CCC proteins reside within the KCC rather than the NKCC clade across the biological kingdoms [12,15,16]. It was emphasized that despite Na^+^-dependent cotransport by AtCCC, plant CCCs could be more closely related to the animal KCC K^+^-coupled cotransporters and thus conform to the relationship between phylogeny and ion specificity of KCCs and NKCCs [12]. Based on this predicament in the present study, we hypothesize that plant CCCs might display the electroneutral KCC (K^+^, Cl^−^ in 1:1 ratio) transport activity rather than the NKCC (K^+^-Na^+^-Cl^−^) activity.

Cryogenic electron microscopy (cryo-EM) structures of human NKCC1 (hNKCC1) [17], *Danio rerio* (zebrafish) NKCC (DrNKCC1) [18], human KCC1 (hKCC1) [19], and mouse KCC4 [20] were determined. These CCC proteins form stable homo-dimers, where each dimer consists of an N-terminal transmembrane α-helical domain (TMD) and a C-terminal α/β fold domain (CTD). The TMD component folds into 12 α-helices, enclosing highly conserved residues that form a single K^+^-binding site and two Cl^−^-binding sites [21]. However, one of the Cl^−^-binding sites [(SCl (1)] in human KCC is believed to be artificial [19]. In addition, NKCC proteins contain residues that coordinate Na^+^, but they are absent in KCC proteins [19,21]. Based on atomistic simulations, it was revealed that hNKCC1 adopts a rocking-bundle mechanism through cooperative angular motions of transmembrane α-helices [22].

Although the atomic structures of the plant CCC proteins are unknown, the breakthroughs in animal CCCs suggested the potential residue identities underlying permeation in plant CCCs, using, e.g., comparative structural modeling and bioinformatics. Yet, the confirmation of the roles of essential residues for ion binding in plant CCCs remains unclear. This study focuses on the structural and transport properties of VvCCC from grapevine. At sequence and structural levels, VvCCC shows the hallmarks of KCC rather than NKCC proteins. The net ^86^Rb flux thermodynamics of VvCCC in *Xenopus* oocytes revealed the KCC-like transport properties and voltage-clamp electrophysiology explained Na^+^ conductance reported previously with VvCCC-expressing oocytes [3]. Our data indicate that VvCCC may function as a K^+^ and Cl^−^ cotransporter in heterologous expression systems, which agrees with phylogenetic analyses [3,12]. Future work is required to determine the precise activity of CCC transport proteins in planta, and whether KCC-like symporter activity is a conserved feature of CCCs across the plant kingdom.

## 2. Results and Discussion

### 2.1. Plant and Animal CCC Proteins Share Residues for K^+^ but Not for Na^+^ Binding

**Figure 1:**
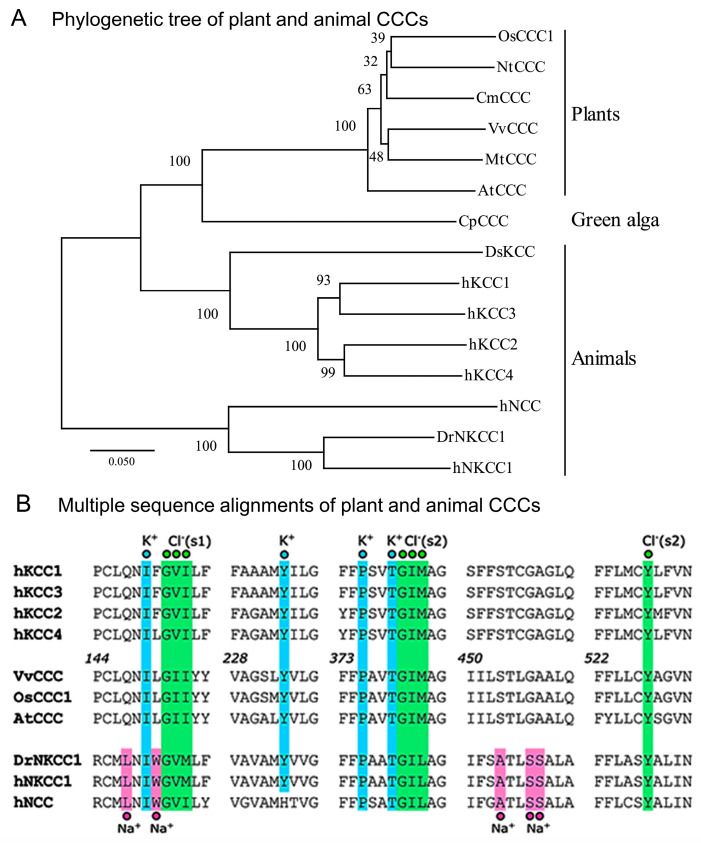
Plant CCC proteins display phylogenetic hallmarks of KCC cotransporters. (**A**) Bootstrapped cladogram of representative plant and animal CCCs inferred from 1000 replicates. Scale = substitution per site. (**B**) Sequence alignments of K^+^-(cyan), Na^+^-(magenta), and Cl^−^-(green) binding sites (s1 and s2). Numbers in italics indicate residue positions in VvCCC.

**Figure 2:**
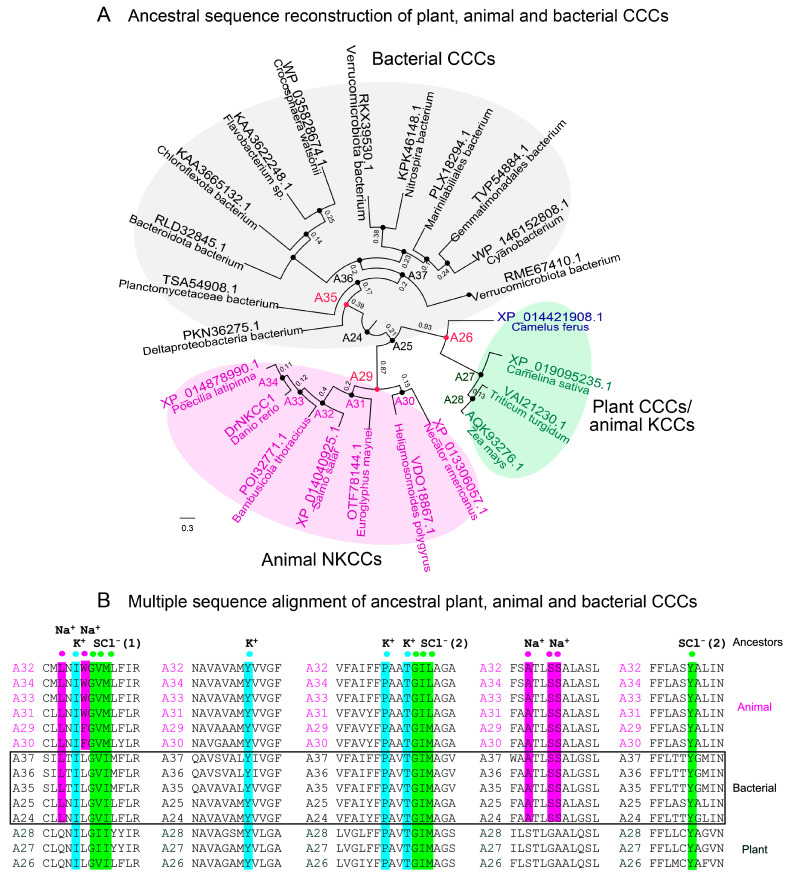
Ancestral sequence reconstruction with FireProt^ASR^ (https://loschmidt.chemi.muni.cz/fireprot, accessed on 25 October 2024) estimates the evolutionary history of CCCs across biological kingdoms. Scale = substitution per site. (**A**) Three clades of CCCs are indicated, where animal KCC and plant CCCs form one clade. (**B**) ProMals3D alignment of animal (magenta), bacterial (black; also framed), and plant (green) CCC ancestral sequences A24-A37. Descriptors above alignments indicate residues forming K^+^-(cyan), Na^+^-(magenta), and two Cl^−^-(green) binding sites (SCl (1) and SCl (2)).

The atomic structures of animal CCCs revealed the identity of residues in ion binding [18,19]. To define the phylogenetic relationships between plant and animal taxa, we analyzed their evolutionary relationship (Figure 1A, Supplementary Figure S1) and the roles of residues in ion binding (Figure 1B). The cladogram of 15 selected CCC proteins across plants and animals showed that OsCCC1, NtCCC, CmCCC, VvCCC, MtCCC, AtCCC, and green alga CpCCC, associated with the animal clade of KCCs rather than NKCCs. Further, a larger cladogram of 76 sequences showed that they segregated in plant CCCs (and protist green alga), animal KCCs, animal NCCs, animal NKCCs, and bacterial CCCs (Supplementary Figure S1, Supplementary Dataset S1). Multiple sequence alignments of VvCCC, AtCCC, and OsCCC1 with animal CCCs disclosed the residues of K^+^-binding sites, which were conserved (Figure 1B). Here, animal TMDs contain two Cl^−^-binding sites coordinated by two sets of three consecutive residues and tyrosine [18,19] (Figure 1B). Plant and animal CCCs had comparable signatures for the first Cl^−^-binding site Cl^−^ (s1) with isoleucine replaced by valine in an equivalent position 151 in VvCCC/AtCCC/OsCCC1 (Figure 1B). For the second Cl^−^-binding site Cl^−^ (s2), the triplets of GIM/GIL and tyrosine carried conserved features in CCCs. It was vital to validate that while NCCs and NKCCs formed a Na^+^-binding site with the LNIW and ATLSS motifs [17,18], these patterns were replaced by the QNIL and STLGA signatures and, thus, were absent in plant CCCs (Figure 1 and Figure 2).

The following ancestral sequence reconstruction (ASR) of CCCs using FireProt^ASR^ [23] assessed the evolutionary relationship of plant CCC cotransporters and deduced the tree of mined sequences across three phyla, and the ancestral nodes (Figure 2A). The plant entries (Figure 2A, green), which have evolved from ancestor 24, formed a clade with animal KCCs. Conversely, bacterial CCCs and animal NKCCs (Figure 2A, grey and magenta, respectively) formed separate clades. In this analysis, 22 ancestral sequences were identified partaking during the evolution of CCCs, with plant and animal KCC ancestors carrying the QNIL and STLGA motifs, while bacterial and animal CCCs with the Na^+^-binding site had the LNIL (W/F) and ATLSS signatures (Figure 2B, Supplementary Datasets S2 and S3). Ancestors A26, A29, and A35 (Figure 2A; red) represent the likely progenitors of plant and animal KCCs, animal NKCCs, and bacterial CCCs, respectively.

We also examined the presence of phosphorylation sites using NetPhos v3.1 [24], and serine/threonine and tyrosine kinase sites with GPS v6.0 [25] in VvCCC, AtCCC [12], and human hKCC1 [19], and compared with DrNKCC1 [22]. In plant CCCs, we found more phosphorylation sites positioned at N-terminal extensions and α-helical bundle TMD regions (at serine, threonine, and tyrosine residues), while in DrNKCC1, the ratio of N-terminal versus C-terminal sites was approximately 1:1 (respective 50 and 52 sites). Predictions of serine/threonine/tyrosine kinase sites in VvCCC (respective 24 and 11 at N- and C-terminal regions) and DrNKCC1 (respective 27 and 25) matched the phosphorylation sites, with the majority catalyzed by AGC, CK1, CMGC, and tyrosine kinase families.

In summary, based upon the primary sequence analyses, plant CCC proteins, similarly to human KCCs, did not share Na^+^-binding residues (Figure 1 and Figure 2), indicating that VvCCC may coordinate the neutral K^+^-Cl^−^ pair rather than Na^+^-K^+^-2Cl^−^ for ion cotransport.

### 2.2. Structural Modeling Reveals That K^+^ and Not Na^+^ Is the Preferred Cation for VvCCC

**Figure 3:**
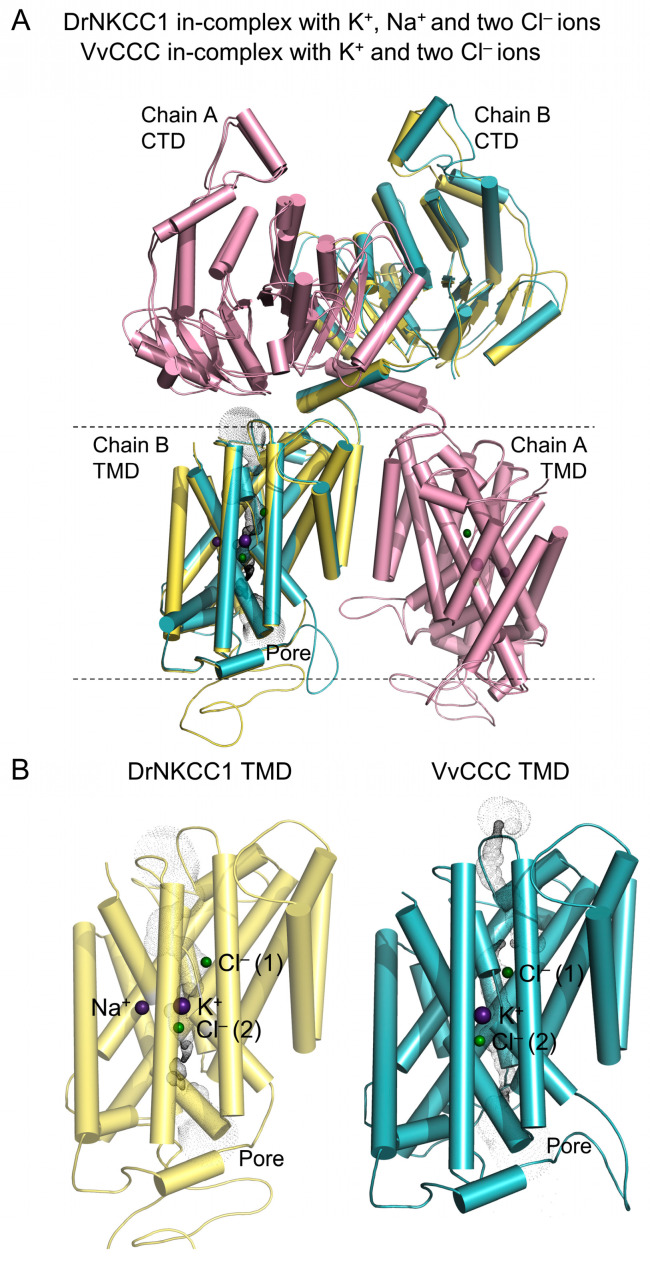
Cryo-EM structure of DrNKCC1 and full-length molecular model of VvCCC in-complex with ions. (**A**) Cartoon representations of superposed DrNKCC1 and VvCCC viewed from the membrane plane (rmsd value 0.94 Å over Cα carbons of 1720 and 1696 residues, respectively). Cartoons illustrate the dispositions of dimers with cylindrical α-helices in chains A or B (pink for DrNKCC1, yellow and cyan for VvCCC) with pore TMDs. The pore of DrNKCC1 accommodates K^+^ and two Cl^−^ ions (cpk spheres) with Na^+^ bound near K^+^, while pores of VvCCC accommodate K^+^ and two Cl^−^ ions. Membrane boundaries are indicated in black dashed lines. (**B**) Cartoon representations of TMDs (chains B) of DrNKCC1 (yellow) and VvCCC (cyan), illustrating poses of ions (cpk spheres) in pores. Geometries of pores in TMD regions, calculated by HOLE [35], were visualized through the sets of grey dots distributed continuously across the pore diameters.

**Figure 4:**
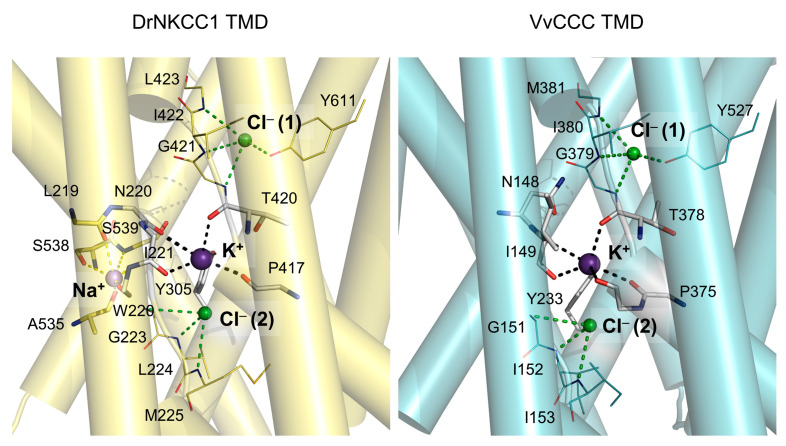
Details of TMDs of the DrNKCC1 cryo-EM structure (chain B; (**left**) panel) and VvCCC molecular model (chain B; (**right**) panel) in-complex with ions (cpk spheres) illustrate the poses of K^+^ and docked Na^+^ in DrNKCC1, and two Cl^−^ ions [Cl^−^ (1) and Cl^−^ (2)] in pores. Residues participating in ion binding are: yellow cpk sticks for Na^+^ in DrNKCC1, atomic-colored cpk sticks for K^+^ in VvCCC, and yellow and cyan cpk lines for two Cl^−^ in DrNKCC1 and VvCCC, respectively. Separations in dashed lines between residues and K^+^ (black), Na^+^ (yellow) and Cl^−^ (green) are between 2.8 Å and 3.1 Å (K^+^), 2.1 Å and 3.0 Å (Na^+^), and 3.1 Å and 3.7 Å (two Cl^−^) for DrNKCC1, and between 2.8 Å and 3.6 Å (K^+^), and 3.3 Å and 3.8 Å (2Cl^−^) for VvCCC.

Structural modeling was performed to investigate ion binding patterns of plant CCC proteins, given the absence of atomic structures (Figure 3 and Figure 4). The most suitable template for VvCCC identified by Phyre2 and LOMETS was the DrNKCC1 cryo-EM structure in a complex with K^+^ and two Cl^−^ ions [18]. The second and third considered templates were hKCC1 [19] and hKCC3 [26], in a complex with K^+^ and two Cl^−^ ions. In these structures, the two Cl^−^ ions bind at Cl^−^ (1) and Cl^−^ (2) sites, with the latter site localized on the surface of the structure, which is deemed to be artificial. However, the possibility remains that it could support the structural integrity of K^+^-binding sites in animal KCCs [19,26]. The hKCC1 and hKCC3 templates were considered less favorable due to substantial mismatches in secondary structure distributions between TMDs of VvCCC and both hKCCs, in addition to a higher gap presence (Supplementary Table S1; Supplementary Figure S2). These mismatches and gaps were due to a 120-residue-long loop in TMD of hKCC1 or hKCC3 between TM4 and TM5, which was shorter in DrNKCC1 (32 residues), hNKCC1 (18 residues), and VvCCC (12 residues). This 120-residue loop is unique to animal KCCs [22] and likely forms a gate. Therefore, it is possible that plant and animal CCCs could be differentially gated, although further research is needed.

The additional analysis of TMD displacements indicated a higher structural similarity in packing between DrNKCC1 and VvCCC when using the DrNKCC1 template compared to any KCC templates, thus validating DrNKCC1 as a preferred template.

The 3D models of full-length VvCCC revealed the overall architectures consisting of a dimeric quaternary assembly (each dimer with two protomers) related by a pseudo-twofold symmetry axis. These folds formed the α-helical TMD and α/β folds CTD regions (Figure 3; boundary marked in dashed lines) with K^+^ and two Cl^−^ ions bound in the TMD pores (Figure 3 and Figure 4). The docked Na^+^ in both dimers of DrNKCC1 was placed in a small cavity, bounded by α-helical breaks and four short α-helices, at around 6–7 Å distance from the K^+^ ion. The Na^+^ ion in DrNKCC1 was coordinated by two Ser, and Ala and Leu residues substituted in plant CCCs by Ala, Gly, Ser, and Gln, respectively (Figure 4). It would appear that Na^+^ and K^+^ ions could compete in animal CCCs for binding sites in pores and cavities with opposing strengths [18,19,26,27]. Interestingly, the AlphaFold Protein Structure Database [28] contains the 3D monomeric VvCCC model (accession AF-F6HLW8; likely modeled on hKCC1), with the root-mean-square-deviation (rmsd) value of 1.8 Å between the two structures. We also generated the dimeric model of VvCCC using Cosmic^2^ [29] with the rmsd value of ~6 Å to VvCCC (modeled on DrNKCC1 in this work) and nearly identical profiles of secondary structure elements.

The ConSurf and ProMals3D sequence analyses of residues that participated in K^+^, Na^+^, and Cl^−^ ion binding showed that the residues at the K^+^, and two Cl^−^-binding sites were highly conserved between DrNKCC1, hKCC1, and VvCCC (Figure 1, Figure 2 and Figure 4). Conversely, the sites for the second cation (Na^+^ or K^+^) showed the absence of conservation between VvCCC and DrNKCC1 but strong conservation between VvCCC and hKCC1 (Figure 4). These comparisons suggested that although VvCCC and DrNKCC1 were structurally similar, the site that could potentially bind Na^+^ in VvCCC resembled that of hKCC1 (Figure 4). The other possibility coexisted that VvCCC binds two K^+^ and two Cl^−^ ions, where the pose of the second K^+^ ion would be in the predicted Na^+^ pose of DrNKCC1 [18,19,27]. The ion binding sites modeled in this work showed characteristics consistent with cation/anion environments, where, in the former case, carbonyl oxygens participated in the binding of cations, while main-chain amide groups secured the positions of anions. Notably, the cation-binding sites of CCCs were reminiscent of the Na^+^-binding sites in HKT transporters with participating carbonyl oxygens [30,31,32,33,34].

The ConSurf sequence conservation patterns (Table 1), based on the VvCCC and AtCCC models in-complex with ions, revealed high scores of residues (9.9 and 9.8) and those of SCl (1) and SCl (2) sites (9.8/9.9) involved in K^+^- and Cl^−^-binding. It could be predicted that VvCCCs and AtCCCs bind K^+^ and Cl^−^ in a 1:1 ratio or K^+^ and Cl^−^ in a 2:2 ratio to assure electroneutral binding. The third possibility of binding K^+^, Na^+^, and Cl^−^ in a 1:1:2 ratio is unlikely, given the conservation patterns of a putative Na^+^-binding site (Table 1, Supplementary Table S1). This is supported by analyses of 150 homologous sequences to VvCCC and 250 sequences to AtCCC, where the STLGA signature for the second cation binding is conserved in VvCCC, AtCCC, and hKCC1, implying that Na^+^-binding is absent.

**Figure 5:**
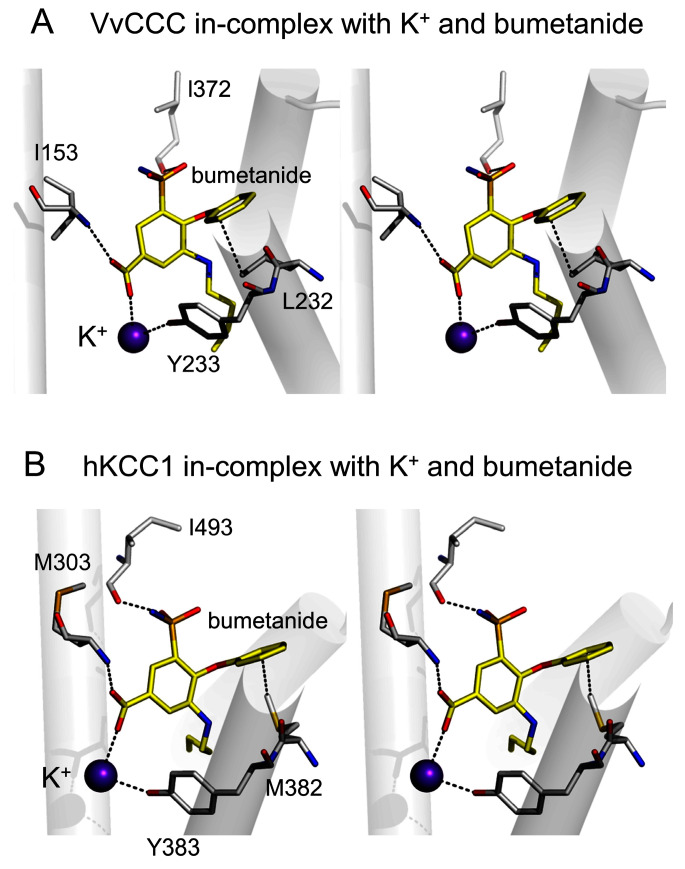
Cross-eyed stereo views illustrating dispositions of K^+^ and bumetanide in TMD pores of VvCCC and hNKCC1. In both panels, interacting residues are indicated in cpk sticks, and separations between residues, K^+,^ and bumetanide is shown at ≤3.6 Å as dashed lines. (**A**) VvCCC and (**B**) hNKCC1 complexes illustrate poses of K^+^ (cpk spheres) and bumetanide (yellow cpk sticks) on the background of selected TMD α-helices.

We also predicted the binding position of bumetanide in VvCCC to reveal its inhibitory mechanism through structural modeling and bioinformatics and taking advantage of a bumetanide pose identified in electroneutral hNKCC1 [37,38] (Figure 5). Intriguingly, bumetanide binding in hNKCC1 involved the input of a carboxy-terminal region through long-distance conformational coupling mediated via movements of N- and C-terminal regions [37,38]. In the VvCCC–bumetanide complex, we observed that the inhibitor was wedged in the α-helical TMD region near K^+^ (Figure 5A), which was comparable with the hNKCC1 complex [37,38]. We noted that VvCCC formed hydrophobic I153, L232, and L372 contacts, and Y233 a hydrogen bond with bumetanide, which corresponded to interactions seen in hNKCC1 via M303, M382, I493, and Y383 residues (Figure 5B).

Taken together, 3D modeling and bioinformatics demonstrated that VvCCC shared structural features that are typical of animal KCC proteins (e.g., hKCC1). Further, the dispositions of ion-binding residues revealed that VvCCC could bind K^+^-but not Na^+^.

### 2.3. VvCCC Is Intracellular in Yeast and HEK293 Cells

Transport properties of CCCs have been traditionally investigated in *Xenopus laevis* oocytes using radiotracers such as ^86^Rb, which, by its atomic nature, mimics Na- and K-containing alkali metals. However, radionuclides are hazardous and become more difficult to source. So, alternative methods, such as fluorescent probes in mammalian cells or whole-cell perforated patch-clamping, are often used [39]. These assays require that CCCs localize to the plasma membrane. However, in HEK293 cells, a VvCCC-DsRed fusion protein showed an intracellular signal that did not co-localize with the plasma membrane (CellMask^TM^) or nuclear (Hoechst) stain markers (Supplementary Figure S3A). Thus, HEK293 cells could not be used for functional investigations. Factors influencing VvCCCs subcellular localization and its membrane targeting in HEK293 cells remain to be open, although it is known that phosphorylation influences the plasma membrane targeting of hKCC2 in HEK293 cells [40,41].

Growth of Na^+^-sensitive and K^+^-uptake deficient yeast was used previously to functionally characterize rice OsCCC1 as a K^+^ and Na^+^ cotransporter [11]. We used this approach and expressed the VvCCC-YFP fusion protein in yeast and stained cells with the FM4-64 dye, which stains the plasma membrane of live cells by intercalation before being internalized (Supplementary Figure S3B). Here, the perimeter of yeast cells and some intracellular compartments remained visible when stained with FM4-64, while the signal did not co-localize at endocytic vesicles of yeast.

In conclusion, using confocal laser-scanning microscopy, we showed that the injection of cRNA encoding the *VvCCC-YFP* fusion into oocytes generated a fluorescent signal at the plasma membrane, while this fluorescence was undetectable in water-injected control oocytes [3]. Therefore, the *VvCCC* cRNA-injected oocytes were used for transport studies described below.

### 2.4. VvCCC Displays KCC-like ^86^Rb Flux Dynamics in Xenopus laevis Oocytes

**Figure 6:**
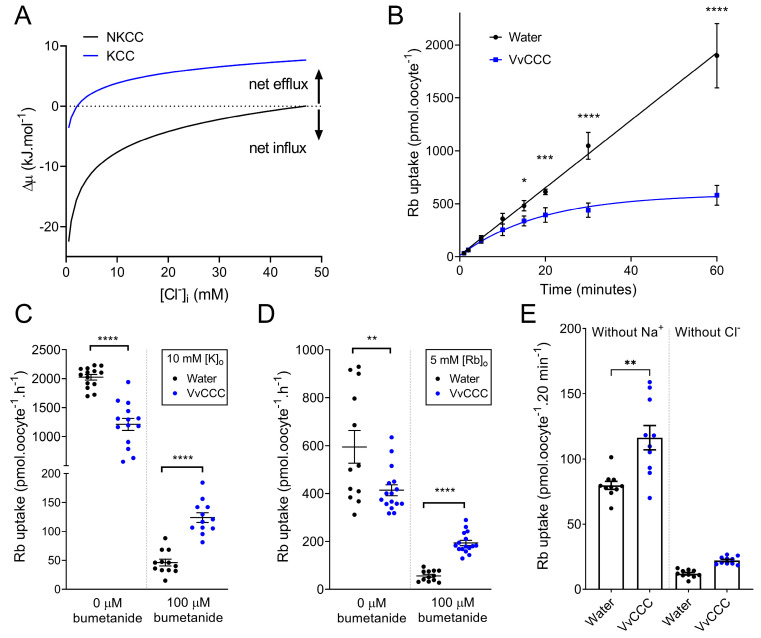
VvCCC-mediated ^86^Rb tracer fluxes display thermodynamic hallmarks of KCC cotransporters. (**A**) Predicted thermodynamic driving forces for KCC and NKCC cotransport into *Xenopus* oocytes as a function of [Cl^−^]_i_ in the ND96 solution with 106.6 mM [Cl^−^]_o_, 96 mM [Na^+^]_o_, and 2 mM [K^+^]_o_. Driving forces were calculated assuming 100 mM [K^+^]_i_ and 10 mM [Na^+^]_i_ at 25 °C using the following equations: ${\Delta µ}_{KCC}=RTln\frac{{\left[K^{+}\right]}_{i\;}}{{\left[K^{+}\right]}_{o}}+\frac{{\left[{Cl}^{-}\right]}_{i}}{{\left[{Cl}^{-}\right]}_{o}}$, and ${\Delta µ}_{NKCC}=RTln\frac{{\left[{Na}^{+}\right]}_{i}{\left[K^{+}\right]}_{i\;}{\left[{Cl}^{-}\right]}_{i}^{2}}{{\left[{Na}^{+}\right]}_{o}{\left[K^{+}\right]}_{o}{\left[{Cl}^{-}\right]}_{o}^{2}}$, where R is the gas constant (8.314 kJ·mol^−1^·K^−1^) and T is the absolute temperature. The dashed line represents FRP. (**B**) Time course of ^86^Rb uptake in oocytes injected with VvCCC or water in standard ND96 solution. Oocytes were pre-incubated in Cl^−^-free ND96 overnight before measurements (Cl^−^ replaced with gluconate). Each data point is the mean ± standard deviation (SD, calculated in Microsoft Xcel 2019) of six oocytes (water) or eight oocytes (VvCCC). Asterisks represent significant differences at single time points (one-way ANOVA). (**C**,**D**) ^86^Rb uptake in oocytes injected with VvCCC or water after 1 h in ND96 with 10 mM KCl (**C**) or 5 mM RbCl (**D**) in the absence or presence of 100 µM bumetanide. Bars indicate mean ± standard error of the mean (SEM, calculated in Microsoft Xcel 2019). Asterisks represent significant differences between means (unpaired *t*-test). (**E**) ^86^Rb uptake in oocytes injected with VvCCC (blue) or water (black) after 20 min in ND96 without Na^+^ (left) or without Cl^−^ (right). Bars indicate mean ± SEM. Asterisks represent significant differences between means (unpaired t-test). For all panels, asterisks denote * *p* < 0.05 ** *p* < 0.01 *** *p* = 0.001 **** *p* < 0.0001.

Payne (1997) [6] calculated thermodynamic driving forces for hKCC2 in neurons as a function of external K^+^. Similar calculations were performed for NKCC [42], which predicted the expected outcomes of NKCC and KCC flux experiments in *Xenopus* oocytes at different Cl^−^ internal concentrations ([Cl^−^]_i_). In *Xenopus* oocytes, CCC-mediated tracer uptake is typically investigated after pre-incubation in Cl^−^-free medium, which reduces [Cl^−^]_i_ to negligible levels [43]. We adopted an alternate approach and found out that at low [Cl^−^]_i_, the chemical driving force (Δµ) for NKCC transport was negative in standard ND96 solution (96 mM NaCl; 2 mM KCl), meaning that the driving force for coupled 1Na^+^:1K^+^:2Cl^−^ cotransport was inward (Figure 6A, black dotted line). By contrast, KCC operated closer to the predicted flux reversal point (FRP), where net flux equaled zero (Figure 6A, blue line). Cl^−^-depleted oocytes with expressed KCC would have a negative Δμ initially, but FRP would be reached when [Cl^−^]_i_ accumulated to 2.4 mM, compared to 46.7 mM for NKCC (Figure 6A, X-axis intercepts). Thus, in oocytes depleted of [Cl^−^]_i_, thermodynamics evaluations predicted that ^86^Rb uptake through a KCC would rapidly achieve equilibrium while NKCC would mediate ^86^Rb influx longer (Figure 6A).

**Figure 7:**
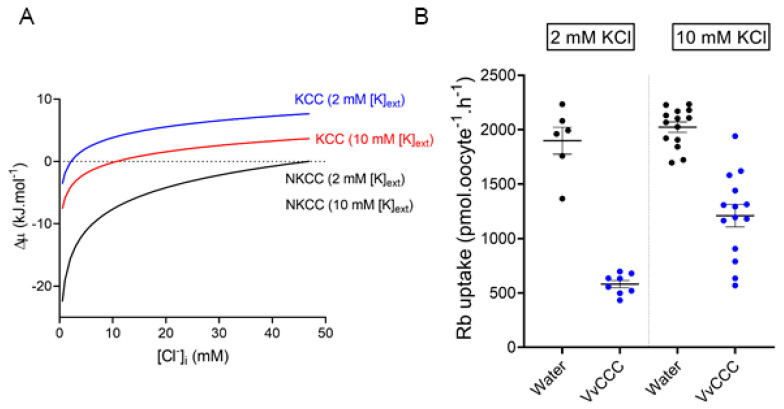
The driving force for VvCCC-mediated ^86^Rb uptake is positively correlated with external [K^+^]. (**A**) Predicted thermodynamic forces for KCC and NKCC cotransport in *Xenopus* oocytes as a function of [Cl^−^]_i_ in ND96 solution with 2 mM [K^+^]_o_ or 10 mM [K^+^]_o_. Calculations were performed as described in Materials and Methods. The flux reversal point is indicated by the intercept with the horizontal dotted line. Changes in [K^+^]_o_ alter predicted FRP for a KCC mechanism (blue and red lines) but not for an NKCC mechanism (black line). (**B**) ^86^Rb uptake in oocytes injected with VvCCC (blue circles) or water (black circles) after 1 h in ND96 with 2 mM KCl (left) or 10 mM KCl (right). Note that increasing [K^+^]_o_ only changed the magnitude of ^86^Rb uptake by *VvCCC*-injected oocytes.

VvCCC flux dynamics in ND96 solution was compared with thermodynamic predictions using an hour time course of ^86^Rb uptake by Cl^−^-depleted oocytes. The *VvCCC*-injected oocytes reached saturation with a half-time of 13.23 min determined from the one-phase association curve (R^2^ = 0.92) (Figure 6B). By contrast, water-injected (control) oocytes accumulated significantly more ^86^Rb, and the uptake displayed a linear relationship (R^2^ = 0.96) (Figure 6B). These results were consistent with thermodynamic predictions that VvCCC mediated KCC cotransport and that NKCC cotransport occurred in water-injected control oocytes, which was mediated by their endogenous NKCC [44] (Figure 6A). The ^86^Rb uptake by control oocytes was almost completely inhibited by 100 µM of bumetanide, supporting that endogenous NKCC is responsible for ^86^Rb uptake in control oocytes (Figure 6C,D). When [K^+^]_o_ was increased from 2 mM to 10 mM, ^86^Rb uptake by the *VvCCC*-injected oocytes increased, while ^86^Rb uptake by the water-injected controls remained unchanged (Figure 6B,C and Figure 7). This agrees with a predicted shift in FRP for a KCC-mediated uptake mechanism by VvCCC but lacks a shift in FRP for an NKCC-mediated uptake mechanism of control oocytes (Figure 6). Replacing [K^+^]_o_ with 5 mM Rb^+^ led to similar outcomes but with a reduced total ^86^Rb uptake, which might point to differences in VvCCC selectivity or batch-to-batch oocytes (Figure 6D). Alternatively, these observations may be explained by differences in apparent affinity constants for both ions. Unlike the water-injected control oocytes, ^86^Rb uptake by *VvCCC*-injected oocytes was partially blocked by 100 µM bumetanide (Figure 6C,D). This is consistent with ^86^Rb uptake data by *VvCCC*-injected oocytes [3]. Bumetanide affinity to KCCs is significantly lower than that of NKCCs. NKCCs have a typical half-maximal effective concentration (EC_50_) of ~0.1 µM, while the typical EC_50_ value for KCCs is ~100 µM bumetanide [4].

To determine if VvCCC-mediated ^86^Rb uptake was Na^+^ coupled, Na^+^ was removed from the medium. Fluxes were carried out for 20 min, as VvCCC would have already reached saturation. In Na^+^-free ND96, the *VvCCC*-injected oocytes accumulated more ^86^Rb than the water-injected controls (Figure 6E). When Cl^−^ was removed from the flux solution, neither *VvCCC*-injected nor water-injected controls accrued significant ^86^Rb, suggesting that ^86^Rb uptake was Cl^−^-coupled for both transport pathways (Figure 6). These data indicated that VvCCC has the K^+^-Cl^−^-like cotransporter activity.

### 2.5. VvCCC Elicits Na^+^ Conductance in Xenopus Oocytes but Is Independent of Electroneutral VvCCC Function

**Figure 8:**
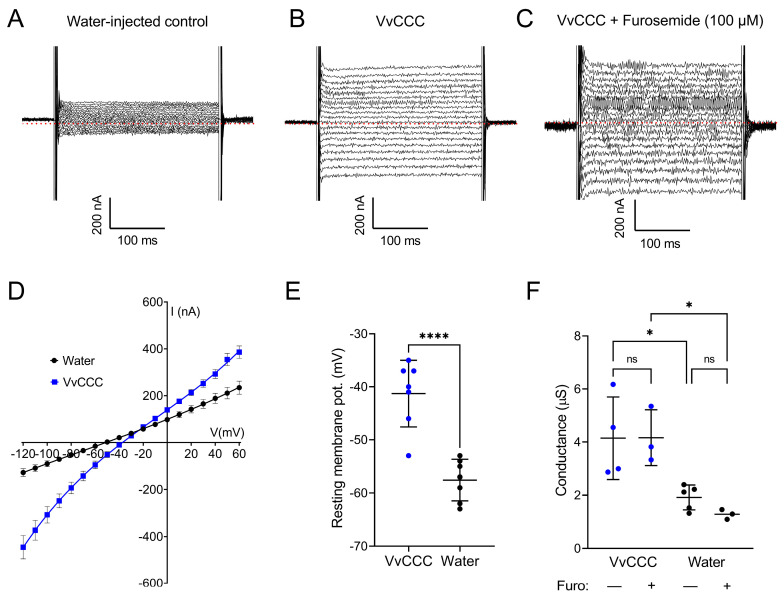
*VvCCC* expression induces an electrogenic cation conductance in *Xenopus* oocytes. (**A**–**C**) Whole-cell electrophysiology traces showing currents recorded from water-injected control oocytes (**A**), *VvCCC*-injected oocytes (**B**), and *VvCCC*-injected oocytes pre-incubated for 30 min and recorded in the presence of 100 µM furosemide (**C**). Dotted red lines denote zero current level. (**D**) Current-voltage relationships of oocytes injected with VvCCC (blue) or water (black) in ND96 solution. Data are the mean ± SEM of four oocytes. Curves were generated by fitting data with a polynomial function. (**E**) Resting membrane potential of oocytes injected with VvCCC (blue) or water (black) in the ND96 solution. Data indicate mean ± SEM. Asterisks denote statistically significant differences (unpaired *t*-test). (**F**) Whole-cell conductance of oocytes injected with VvCCC (blue) or water (black) pre-incubated for 30 min with or without 100 µM furosemide. Conductance was determined from the slope of the I/V curve close to the reversal potential. Asterisks denote statistically significant differences (one-way ANOVA with Tukey’s multiple comparison test). In all panels, asterisks denote * *p* < 0.05 **** *p* < 0.0001.

Previously, the expression of plant CCCs in oocytes led to a higher ^22^Na influx, and the conclusion was made that plant CCCs have NKCC characteristics [3,12]. To explore this suggestion, two-electrode voltage-clamping (TEVC) was performed in the ND96 solution. Whole-cell currents of *VvCCC*-injected oocytes were greater than those of water-injected controls (Figure 8A–C). Currents were inward at physiological resting membrane potential (V_m_), consistent with a cation influx (Figure 8D). Resting V_m_ in VvCCC-expressing oocytes was depolarised compared to water-injected controls (Figure 8E). This is expected for oocytes in 100 mM [Na^+^]_ext_ that express an electrogenic Na^+^ conductance, as the Nernst equation predicts that V_m_ will move towards the equilibrium potential for Na^+^. To determine if this conductance was mediated by VvCCC, the oocytes were pre-incubated and clamped in 100 µM furosemide. In hNKCCs, furosemide binds at the cytosolic CTD and bends the structure of hNKCC through rigid body movement toward the membrane surface by ~9 Å compared to apo-hNKCC [37].

Upon 100 µM furosemide application, we observed that the conductance of *VvCCC*-injected oocytes changed only slightly (Figure 8F), as it is known that furosemide is not a high-affinity inhibitor of KCCs. It was reported that its K_i_ value was around 180 uM for KCC1 and lower (900 µM) for KCC4 with CCCs expressed in oocytes [45]. Furosemide did not affect the conductance of control oocytes, which was much lower than in the *VvCCC*-injected oocytes in its presence or absence (Figure 8F). No Cl^−^ conductance was observed in this case, but the conductance mediated inward K^+^ currents under high external K^+^ (Supplementary Figure S4).

Collectively, our results indicate that an electrogenic Na^+^ (and K^+^) conductance is activated in *VvCCC*-injected oocytes and is independent of electroneutral VvCCC function. This conductance was formerly unidentified and would likely contribute towards the ^22^Na uptake, which was observed earlier [3]. Consistent with our findings, the expression of mosquito AeCCC2 from *Aedes aegypti* in *Xenopus* oocytes induced the Na^+^ and Li^+^ conductance of a similar amplitude to VvCCC, which was not inhibited by loop diuretics [46]. Cation conductance might be an inherent property of AeCCC2 and VvCCC. However, the induction of endogenous channels by high levels of heterologous membrane proteins in *Xenopus* oocytes was also reported [47]. Whether AtCCC also could elicit a cation conductance in oocytes is uncertain, which is another experimental target.

### 2.6. How Might a KCC-like Protein Function in Plant Cells?

In plants, Golgi, TGN, and EE networks are parts of an endocytic pathway, which are important for cellular trafficking and recycling to and from a plasma membrane [48]. A progressive pH gradient (acidic lumen) is required for normal endosomal function, driven by the coordinated action of the proton (H^+^) pumping of V-type ATPase along with a predicted CLCd (Cl^−^/H^+^ exchange channel) and known KEA, NHX5, NHX6, CHX17 K^+^/H^+^ exchangers to balance charge. Arabidopsis AtCCC was proposed to complete the transport circuit in TGN/EE networks through the coupled efflux of cations and anions out of the lumen towards the cytoplasm [8]. Under non-stress conditions, the cytosolic K^+^ concentration is around 100 mM [49], while that of Cl^−^_cyt_ is around 15 mM [50,51]. The K^+^ and Cl^−^ concentrations in the TGN/EE lumen are unknown but are likely higher than in the cytoplasm as both ions are actively transported against their electrochemical gradients. This would support the CCC-mediated efflux mechanism from endosomes [49]. The magnitude of CCC-mediated fluxes could be influenced by the combined activity of secondary active proton exchangers located at endo-membranes. Plasma membrane K+ and Cl may also influence CCC fluxes^−^ channels generating localized concentration gradients within cytosol, especially as the TGN/EE networks are mobile organelles.

If the CCC activity is modulated, for example, by phosphorylation or de-phosphorylation, the counter-ion concentration and, thus, the pH value within the TGN/EE lumen could be partially regulated. Multiple studies demonstrated that AtCCC is phosphorylated in vivo [24,52]. Interacting partners of plant CCC proteins are unknown; however, the mitogen-activated protein kinase recognition sequence was identified at the N-terminus of AtCCC [53]. AtCCC and its co-localization partner CLCd could act as negative regulators of pathogen-associated molecular pattern-triggered immunity [13,54]; this potentially suggests a new signaling pathway that CCC proteins may be part of.

## 3. Materials and Methods

### 3.1. Phylogenomics, Sequence Alignments, and Ancestral Sequence Reconstruction (ASR)

The phylogenetic tree of representative CCC sequences was constructed in MEGA11 [55] with 15 and 76 plant and animal sequences aligned in MUSCLE [56] using the Neighbour-Joining method with evolutionary distances computed by the *p*-distance method [57]. The Bootstrapped consensus tree was inferred from 1000 replicates. The following protein sequences were used in the analysis of 15 sequences: human hKCC1 (NP_005063.1), hKCC2 (NP_001128243.1), hKCC3 (NP_598408.1), hKCC4 (NP_006589.2), hNKCC1 (NP_001037.1), hNCC (NP_000330.3), *Danio rerio* DrNKCC1 (NP_001002080), *Drosophila simulans* DsKCC (XP_016029323.1), *Vitis vinifera* VvCCC (XP_010655720.1), *Arabidopsis thaliana* AtCCC (NP_849732.1), *Oryza sativa* OsCCC1 (Q6Z0E2.1), *Medicago truncatula* MtCCC (CAJ38499.1), *Nicotiana tabacum* NtCCC (AAC49874.1), *Cucurbita moschata* CmCCC1 (XP_022955334.1), and *Chloropicon primus* (green alga) CpCCC (QDZ20292.1). Accessions and species origins of 76 sequences are detailed in the Supplementary Information. Ancestral sequence reconstruction (ASR) was performed using the FireProt^ASR^ server (https://loschmidt.chemi.muni.cz/fireprot/, accessed on 25 October 2024) [23] in one step using the default parameters for evolutionary models and the bootstrap selection at 20–90% of the DrNKCC1 protein sequence. Additional details are specified in the Supplementary Information.

### 3.2. Identification of the Template for 3D Homology Modeling of Vitis vinifera (VvCCC)

The most suitable template for VvCCC was identified by Phyre2 [58] and LOMETS [59] and through evaluations of 21 structures of NKCC and KCC cotransporters retrieved from the Protein Data Bank (PDB). These sequences were aligned with ProMals3D [60], and distributions of secondary structure elements were analyzed with PsiPred [61].

### 3.3. Protein Structural (3D) Molecular Modeling

The cryo-EM structures of the cation–chloride cotransporters from *Danio rerio* (PDB accession 6npl, chains A and B) (DrNKCC1) [18] and human (PDB accession 6m1y, chains A and B) (hKCC3) [26] in-complex with K^+^ and two Cl^−^ were used as templates for homology modeling of VvCCC. Both templates represent inward-open inactive states [18,19,26,27]. Additionally, human hKCC1 (PDB accession 6kkr, chains A and B) [19] was used as a template. As the atomic structure of DrNKCC1 does not define positions of two Na^+^, in chains A and B, they were docked in TMDs, based on predicted poses [18,19,27]. Top-scoring models were subjected to energy minimization using the knowledge-based YASARA2 forcefield (bond distances, planarity of peptide bonds, bond angles, Coulomb terms, dihedral angles, and van der Waals forces) [62], combined with the particle-mesh-Ewald (PME) energy function for long-range electrostatics (cut-off 8.0 Å) to obtain smoothed electrostatic potentials. To correct the covalent geometry, conformational stress was removed by a short steepest descent minimization (time 5000 fs, 1 fs time steps, 298 K), followed by simulated annealing (time step 1 fs, atom velocities scaled down by 0.9 every 10th step) until convergence (710 steps) with energy improvement of less than 0.05 kJ/mol per atom during 200 steps in YASARA [63]. 3D models of VvCCC in-complex with three ions (K^+^ and two Cl^−^) using the DrNKCC1 and hKCC3 structural templates were generated in MODELER 10v1 [64] as described [30,31,32]. Best-scoring models from the ensemble of 100 models were selected based on the modeler objective function [65], discrete optimized protein energy [66], and statistically optimized atomic potential [67] terms, PROCHECK [68], and ProSa 2003 [69]. Geometries of pores in TMD regions, calculated by HOLE [35], were visualized through the sets of grey dots distributed continuously across the pore diameters. Structural images were generated using PyMOL Molecular Graphics System v2.5.2 (Schrődinger LLC, Portland, OR, USA).

The ensembles of 100 structural models of the VvCCC full-length and TMD domain transporters with K^+^ and two Cl^−^ using the DrNKCC1 and hKCC3 templates were evaluated, and top-scoring models were selected. Stereo-chemical parameters evaluated by PROCHECK (excluding Gly and Pro residues) in DrNKCC1, hKCC3, and VvCCC full-length and TMD structures and models revealed their favorable parameters (Supplementary Table S1), meaning that the VvCCC models were placed in allowed Z-score conformational energy regions [67], and thus these models were reliable.

Bumetanide docking was performed by using a 3D disposition identified in electroneutral hNKCC1 (PDB accession 7smp, chains A and B) [37,38] and HDOCK [70], which is based on a hybrid algorithm of template-based 3D modeling and ab initio-free docking of protein-ligand systems through generating up to 100 docking poses. The top-scoring complexes were energy-minimized in YASARA2 forcefield [62].

### 3.4. Structural Bioinformatics

Sequence conservation patterns of VvCCC, DrNKCC1, and hKCC3 were analyzed with ConSurf [36] at sequence identities between 35% and 95% (specifications: HMMMER homolog search algorithm, UNIREF-90 Protein database with the E-value cut off of 1·10^−4^, Bayesian model of substitution) based on top-scoring VvCCC models.

### 3.5. Thermodynamic Predictions

Thermodynamic driving forces for KCC proteins with K^+^:Cl^−^ stoichiometry (ΔμKCC) were calculated at different [Cl^−^]_i_ using: ${\Delta µ}_{KCC}=RTln\frac{{\left[K^{+}\right]}_{i\;}}{{\left[K^{+}\right]}_{o}}+\frac{{\left[{Cl}^{-}\right]}_{i}}{{\left[{Cl}^{-}\right]}_{o}}$ assuming 100 mM [K^+^]_i_. Thermodynamic driving forces for NKCC proteins with Na^+^:K^+^:2Cl^−^ stoichiometry (ΔμNKCC) were calculated using: ${\Delta µ}_{NKCC}=RTln\frac{{\left[{Na}^{+}\right]}_{i}{\left[K^{+}\right]}_{i\;}{\left[{Cl}^{-}\right]}_{i}^{2}}{{\left[{Na}^{+}\right]}_{o}{\left[K^{+}\right]}_{o}{\left[{Cl}^{-}\right]}_{o}^{2}}$ assuming 100 mM [K^+^]_i_ and 10 mM [Na^+^]_i_. Assumed intracellular Na^+^ and K^+^ concentrations fall within the typical range reported for *Xenopus laevis* oocytes [71]. Extracellular Cl^−^ was set to 106.6 mM [Cl^−^]_o,_ which is the concentration within the ND96 solution. Subscripts *i* and *o* denote intracellular and extracellular compartments, respectively, R is the gas constant (8.314 J mol^−1^ K^− 1^), and T is the absolute temperature (298.15 °K).

### 3.6. Xenopus Oocyte Electrophysiology Experiments

Radiotracer flux experiments were performed as described [3]. Briefly, VvCCC capped RNA (cRNA) was synthesized using the T7 mMessage mMachine Kit (Thermo Fisher Scientific Inc., Waltham, MA, USA). Oocytes were injected with 25 ng of cRNA and incubated in Ringer’s solution at 18 °C for two days. Oocytes were pre-incubated in a Cl^−^-free ND96 solution (Cl^−^ replaced with gluconate) for two hours to overnight, to deplete internal [Cl^−^]_i_. Oocytes were transferred to flux solutions containing 1 µCi of ^86^Rb radionuclide (as RbCl) (Catalogue number NEZ072001MC, Perkin Elmer, Rodgau, Germany). Flux solutions consisted of standard ND96 (96 mM NaCl, 2 mM KCl, 1.8 mM CaCl_2_, 1 mM MgCl_2_, 5 mM HEPES, pH 7.4), or ND96, where 2 mM KCl was replaced with 10 mM KCl or standard ND96 where 2 mM KCl was replaced with 5 mM RbCl. In both cases, the NaCl concentration was adjusted to maintain osmolality. A flux solution consisting of ND96 was prepared, and 96 mM NaCl was replaced with NMDG-Cl. All flux solutions contained 0.1 mM ouabain to block the oocyte Na^+^/K^+^-ATPase, and some solutions included 100 µM bumetanide. Oocytes were pre-incubated in inhibitors for ten minutes before being transferred to a flux solution. Oocytes were rinsed three times in an ice-cold flux solution without radionuclide, transferred individually to scintillation vials, and lysed with 10% (*w*/*v*) SDS. The scintillation cocktail was added, and samples were counted using a liquid scintillation counter (Beckman LS6500, Beckman Coulter Inc., Fullerton, CA, USA). Counts per minute were converted to pmoles per oocyte according to $\frac{\left(CPM\;sample-CPM\;blank\right)}{\left(Specific\;Activity\right)}$. The Specific activity (SA) in 10 µL aliquots of a flux solution was calculated according to:

$$SA=\frac{\left(CPM\;solution-CPM\;blank\right)}{\left(pmoles\;of\;K^{+}\;in\;aliquot\right)}\cdot \mathrm{monovalent}$$

Two-electrode voltage-clamping was performed in the standard isotonic Ca^2+^-free ND96 solution (100 mM NaCl, 2 mM KCl, 5 mM MgCl_2_, 10 mM HEPES, pH 7.4). Resting membrane potentials (V_m_) and whole-cell currents were recorded using a GeneClamp 500B amplifier (Molecular Devices LLC, San Jose, CA, USA) connected to two microelectrodes made of borosilicate glass that were backfilled with 1 M KCl. From a holding potential of −40 mV, oocytes were clamped stepwise from −120 mV to +60 mV for 300 ms, and whole-cell currents were recorded. Data were digitized using a Digidata 1322A (Molecular Devices LLC), and data were analyzed using pClamp version 9.0 software (Molecular Devices LLC).

### 3.7. Microscopy

An expression construct was assembled to visualize the subcellular localization of VvCCC-DsRed in HEK293 cells. pcDNA3.2-DEST vector backbone (Thermo Fisher Scientific Inc.) was linearised by PCR using primers 5′-TTGATCTAGAGGGCCCGCG-3′ and 5′-TGATAGCTTAACTAGCCAGCTTGG-3′. Full-length VvCCC coding sequence without stop codon was amplified from pCR8-VvCCC [3], using primers 5′-CTAGTTAAGCTATCACACCATGGACAACGGAGACATTGA-3′ and 5′-CGATCCTCCTCCTCCTGTGAAAAGGGTGACAACATCT-3′. DsRed was amplified from pAG423GAL-ccdB-DsRed (Addgene plasmid repository, plasmid #14365) using primers 5′-GGAGGAGGAGGATCGATGGACAACACCGAGGACGT-3′ and 5′-GGCCCTCTAGATCAACTACTGGGAGCCGGAGTGG-3′. PCR was performed using Platinum™ PCR SuperMix High Fidelity (Thermo Fisher Scientific Inc.) containing 0.8 µM of forward and reverse primer plus 2.5 ng of a plasmid template. Purified PCR fragments were assembled using Gibson Assembly^®^ Master Mix (New England Biolabs, Ipswich, MA, USA) following the manufacturer’s procedures.

Human Embryonic Kidney 293 (HEK293) cells were grown at 37 °C with 5% (*v*/*v*) CO_2_ in Dulbecco’s modified eagles medium (DMEM). Cells were cultured to 80% confluence in an ibidi 8-well µ-slide (Ibidi GmbH, Gräfelfing, Germany) and transfected with 1 µg of pcDNA3.2-DEST-VvCCC-DsRed using lipofectamine 3000 reagent (Thermo Fisher Scientific Inc.). After two days, the cells were stained for 15 min at 37 °C with Hoechst nuclear stain and CellMask™ Green Plasma Membrane Stain (Thermo Fisher Scientific Inc.). Cells were washed three times with phosphate-buffered saline to remove the unbound stain, resuspended in DMEM, and imaged with an FV3000 Confocal Microscope fitted with an ×40 oil immersion objective lens (Olympus Australia Pty Ltd., Notting Hill, Australia). Excitation/emission conditions were Hoechst (405 nm/430–470 nm), CellMask™ (488 nm/500–540 nm), and DsRed (561 nm/570–670 nm).

For yeast expression, VvCCC-YFP was cloned into pYES-DEST52 (Thermo Fisher Scientific Inc.) by LR recombination with pCR8-VvCCC-YFP [3] with Gateway LR clonase (Thermo Fisher Scientific Inc.). The expression construct was introduced into *Saccharomyces cerevisiae* strain B31 (MATα ena1–4::HIS3 nha1::LEU2) [72] using the lithium acetate procedure. Transformants were selected on a synthetic complete medium without uracil (SC-uracil) at 28 °C. Yeast cells were grown overnight in liquid SC-uracil containing 2% (*w*/*v*) d-glucose at 28 °C with shaking. To induce protein expression, cells were pelleted and resuspended in SC-uracil containing 2% (*w*/*v*) d-galactose and incubated overnight at 28 °C with shaking. Before imaging, cells were incubated in a growth medium containing 10 µg/mL of FM4-64 (Sigma, St Louis, MO, USA), which stains the plasma membrane of living cells before being internalized [73]. Cells were rinsed and imaged using a Nikon A1R confocal laser scanning microscope with ×63 water objective lens and NIS-Elements C software, Ver5.22.00 for Windows (Nikon Corporation, Tokyo, Japan). Excitation/emission conditions were GFP (488 nm/500–550 nm) and FM4-64 (561 nm/570–620 nm).

## 4. Conclusions

In the grapevine cation–chloride VvCCC cotransporter, we detected the absence of a Na^+^-binding site at the structural level, supported by the phylogenomic analyses and electrophysiology measurements. Furthermore, our ion permeation data agree with the thermodynamic predictions for K^+^-Cl^−^ cotransport in VvCCC.

We believe the findings reported in this work might shed new light on the roles of plant CCC membrane proteins and pave the way for future correlative studies, which could inspire bioengineering efforts in creating a sustainable bio-economy.

## Figures and Tables

**Table 1:** Residue conservation scores involved in the binding of K^+^ and two Cl^−^ ions and a putative second cation by VvCCC or AtCCC, examined at sequence identities between 35% and 95%.

Ion	Residue	Aligned	Scores ^2^	Residue Variability ^3^	
	VvCCC ^1^	Sequences		VvCCC	AtCCC
K^+^					
N	ASN148	144/150	9.8	S, N, Q, T, H	S, N, Q, T, H
I	Ile149	144/150	9.9	I, V, M	Q, I, L, V, M
Y	Tyr:233	149/150	9.8	N, S, E, Y, H	N, E, S, H, Y
SCl (1)					
G	Gly379	150/150	9.9	G, D	R, G, D
I	Ile380	149/150	9.9	P, I, F, V	V, Y, I, F, P
M	Met381	149/150	9.9	M, L, F	L, V, M, E, F
Y	Tyr527	141/150	9.9	D, Y	D, C, Y, L
SCl (2)					
G	Gly151	144/150	9.9	S, G	G, S
I	Ile152	144/150	9.9	V, I, T	V, I, T
I	Ile:153	144/150	9.8	L, T, V, I	M, V, L, I, T
Second cation (Na^+^) ^4^					
S	Ser453	141/150	9.9	C, A, S	V, A, S, T, I
G	Gly456	141/150	9.9	G, A	G, S, A
A	Ala457	141/150	7.5	T, M, A, S, L, V, C, F	Q, G, S, A

^1^ Numbering of VvCCC (UniProt accession F6HLW8). ^2^ Conservation scores estimated by the Bayesian method for calculating confidence intervals [36]. ^3^ Residue variability analyzed by ConSurf [36]. ^4^ Residues corresponding to those that coordinate Na^+^ in DrNKCC1 [18].

## Data Availability

The data presented in this study are available upon request from the corresponding author.

## Supplementary Materials

The following supporting information can be downloaded at: https://www.mdpi.com/article/10.3390/ijms252312955/s1. Reference [74] is cited in the Supplementary Materials.

## List of Abbreviations

CCC(s), cation–chloride cotransporter(s); VvCCC, Vitis vinifera CCC; KCC(s), K^+^-Cl^−^ cotransporter(s); NKCC(s), Na^+^, K^+^-Cl^−^ cotransporter(s); SLC12, solute carrier family 12; cryo-EM, cryogenic-electron microscopy; TGN, trans-Golgi network; EE, early endosome; AtCCC, *Arabidopsis thaliana* CCC; OsCCC1, *Oryza sativa* CCC1; DrNKCC1, Danio rerio NKCC1; hNKCC1, human NKCC1; TMD(s), transmembrane α-helical domain(s); CTD(s), C-terminal α/β fold domain(s); SCl(1), Cl^−^ binding site 1; SCl (2), Cl^−^ binding site 2; NtCCC, *Nicotiana tabacum* CCC; CmCCC, *Cucurbita moschata* CCC; MtCCC, *Medicago truncatula* CCC; CpCCC, *Chloropicon primus* (green alga) CCC, DsKCC, *Drosophila simulans* KCC; human hKCC1, human KCC1; hKCC2, human KCC2; hKCC3, human KCC3; hKCC4, human KCC4; hNCC, human NCC; ASR, ancestral sequence reconstruction; AGC, cAMP-cGMP-PKC kinases; CK1, casein kinase 1; CMGC, CDK-, MAPK-, GSK-, CLK-kinases; RMSD, root-mean-square-deviation; cpk, Corey, Pauling, Kultin (atom colouring); FRP, flux reversal point; SEM, standard error of the mean; Vm, resting membrane potential; TEVC, two-electrode voltage-clamping; AeCCC2, *Aedes aegypti* CCC; CLCd, Cl^−^/H^+^ exchange channel; KEA, K^+^ efflux antiporter; NHX5, sodium hydrogen exchanger 5; NHX6, sodium hydrogen exchanger 6; CHX17, cation/proton exchanger 17; PDB, Protein Data Bank.
