# Supplementary material for: Protein Structural Modeling and Transport Thermodynamics Reveal That Plant Cation–Chloride Cotransporters Mediate Potassium–Chloride Symport
Source: Int J Mol Sci. 2024 Dec 2;25(23):12955. doi: 10.3390/ijms252312955 (PMC11641049; doi:10.3390/ijms252312955)
Supplement: Supplementary file 1 [file ijms-25-12955-s001.zip › ijms-3305924-Supplementary Information.pdf]

Supplementary Information

Protein Structural Modeling and Transport Thermodynamics Reveal That Plant Cation–Chloride Cotransporters Mediate Potassium–Chloride Symport

Sam W. Henderson, Saeed Nourmohammadi, and Maria Hrmova*

School of Agriculture, Food and Wine, and Waite Research Institute, Faculty of Sciences, Engineering and Technology, University of Adelaide, Waite Campus precinct, Glen Osmond, South Australia 5064, Australia; sam.henderson@adeladie.edu.au (S.W.H); saeed.nourmohammadi@adelaide.edu.au (S.N.); maria.hrmova@adelaide.edu.au (M.H.).

* Correspondence: maria.hrmova@adelaide.edu.au.

Supplementary Materials and Methods

Phylogenetic tree generation using MEGA11

Step 1: The sequence alignment of representative 15 entries across plant (and algal) CCCs, and animal NKCCC and NCCCs (accession numbers are listed in Materials and Methods of the main article), was performed in MUSCEL [1] with the UPMGA (Unweighted Pair Group Method with Arithmetic Mean) cluster method and the following parameters: Gap open 2.90; Gap extend 0.00; Hydrophobicity multiplier 1.20.

Step 2: The phylogenetic tree was constructed, based on the MUSCLE alignment of 15 entries (detailed above) [1], using the Neighbour-Joining statistical method [2] with evolutionary distances computed by the *p*-distance method [3]. The following parameters were used: Test of phylogeny: Bootstrap method; Number of Bootstrap replications 1000; Substitution model: Amino acid (*p*-distance); Rates and patterns: Uniform; Patterns among lineages: homogenous; Gaps/missing data treatment: pairwise deletion; Number of threads: 8. This analysis was repeated wise, and the same results were obtained.

Using the procedure described above we also generated the cladogram of 76 plant, animal and bacterial CCC proteins. We conducted the BLASTP search (E-value cutoff $<1e^{-5}$ with conditional composition score matrix adjustment) [4], using VvCCC, DrNKCC, hKCC1 and *Flavobacterium* sp. sequences. Hits of around 100 sequences were retrieved, and protein lengths analyzed to remove partial sequences.

Ancestral sequence reconstruction with FireProt^{ASR}

This analysis proceeded in one step at the FireProt^{ASR} server (https://loschmidt.chemi.muni.cz/fireprot) [5] using the protein sequence of the transmembrane α-helical domain (TMD) region in DrNKCC1 (the NKCCC family transporter from *Danio rerio*). For the analysis, we specified the residues (also shown colour-coded below in the Fasta protein sequence) involved in K^+ (I16, Y100, P212, T215; cyan) and Cl^- (G128, G216, I217 and Y406; green) binding, but not those involved in Na^+ (W17, A330, S333, S334; magenta), as they do not exist in plant CCCs (and would not be mined by FireProt^{ASR}, as confirmed by our analysis), in accordance with the cryo-EM structure of DrNKCC1 (PDB accession 6m1y) [6]. This analysis was repeated twice, and the same results were obtained.

Default parameters for evolutionary models and bootstrap selection at 20-90% with DrNKCC1 were recorded in the output configuration file of the FireProt^{ASR} analysis (model=Automatic, gamma=false, frequencies=false, invariants=false, tool=RAxML, bootstrap=50.0, gaps=0.5).

```
>NCBI Reference Sequence NP_001002080.1; solute carrier family 12 member 2 isoform 1
[Danio rerio]; TMD region - 472 amino acid residues
KFGWIKGVLVRCMLNIWGVMLFIRMTWIVGQAGIAYSCIIVIMATVVTTITGCSTSAIATNGFVRGGGAYYLISRSLGPEFGG
SIGLIFAFANAVAVAMYVVGFAETVVELLMDSGLLMIDQTNDIRVIGTITVILLLGISVAGMEWEAKAQIFLLVILITAIFNY
FIGSFIAVDSKKKFGFFSYDAGILAENFGPDFRGQTFFSVFSIFFPAATGILAGANISGDLADPQMAIPKGTLLAILITGLVY
VGVAISAGACIVRDATGIESNFTLISNCTDAACKYGYDFSSCRPTVEGEVSSCKFGLHNDFQVMSVVSGFSPLISAGIFSATL
SSALASLVSAPKVFQALCKDNIYPGIAIFGKGYGKNNEPLRGYFLTFGIALAFILIAELNVIAPIISNFFLASYALINFSVFH
ASLANSPGWRPSFKYYNMWASLAGAILCCVVMFIINWWAALLTNVIVLSLYIYVSYK
```

Cyan: K^+-binding sites
Magenta: Na^+-binding sites
Green: Cl^--binding sites

References

Please note that these references are also included in the article. Their numbering differs in both documents to make the information in this document self-contained.

1. Edgar, R.C. MUSCLE: a multiple sequence alignment method with reduced time and space complexity. *BMC Bioinform.* **2004**, *5*, 113.

2. Saitou, N.; Nei, M. The neighbor-joining method: A new method for reconstructing phylogenetic trees. *Mol. Biol. Evol.* **1987**, *4,* 406–425.
3. Nei, M.; Kumar, S. Molecular Evolution and Phylogenetics. Oxford University Press, New York, **2000**.
4. Altschul, S.F.; Madden, T.L.; Schäffer, A.A.; Zhang, J.; Zhang, Z.; Miller, W.; Lipman, D.J. GAPPED BLAST and PSI-BLAST: a new generation of protein database search programs. *Nucleic Acids Res.* **1997**, *25,* 3389–3402.
5. Khan, R.T.; Musil, M.; Stourac, J.; Damborsky, J.; Bednar, D. Fully automated ancestral sequence reconstruction using FireProt^{ASR}. *Curr. Protoc.* **2021**, *1,* e30.
6. Chew, T.A.; Orlando, B.J.; Zhang, J.; Latorraca, N.R.; Wang, A.; Hollingsworth, S.A.; Chen, D.-H.; Dror, R.O.; Liao, M.; Feng, L. Structure and mechanism of the cation–chloride cotransporter NKCC1. *Nature* **2019,** *572,* 488–492.

Supplementary Table 1. Sequence identities/similarities of VvCCC, AtCCC, and DrNKCC1, hKCC1 and hKCC3 proteins, and 3D protein modelling evaluation parameters of full-length (FL) and transmembrane α-helical domain (TMDs) proteins.

	**Sequence identities / similarities (%)**				
	TMD			Full-length (FL)	
	DrNKCC1	hKCC1	hKCC3	DrNKCC1	hKCC3
AtCCC	33/48	40/54	41/56	29/47	34/53
Gap (%)	14	21	17	15	15
VvCCC	32/49	39/55		29/47	
Gap (%)	15	19		15	

	**3D modelling evaluation parameters**							
	DrNKCC1 template		VvCCC (modelled with DrNKCC1 template)		hKCC3 template		VvCCC (modelled with hKCC3 template)	
	TMD	FL	TMD	FL	TMD	FL	TMD	FL
Number of residues	476	1,720	465	1,696	460	1,460	432	1,312
PROCHECK G scores	0.07	-0.01	0.02	-0.15	-0.01	0.01	-0.01	-0.03
PROCHECK allowed (%)	100	100	100	99.9	100	99.8	99.7	99.6
PROCHECK disallowed (%)	0	0	0	0.1	0	0.2[a]	0.3	0.4
ProSa Z score	-5.5	-8.4	-4.0	-6.6	-4.0	-8.3	-4.3	-6.2

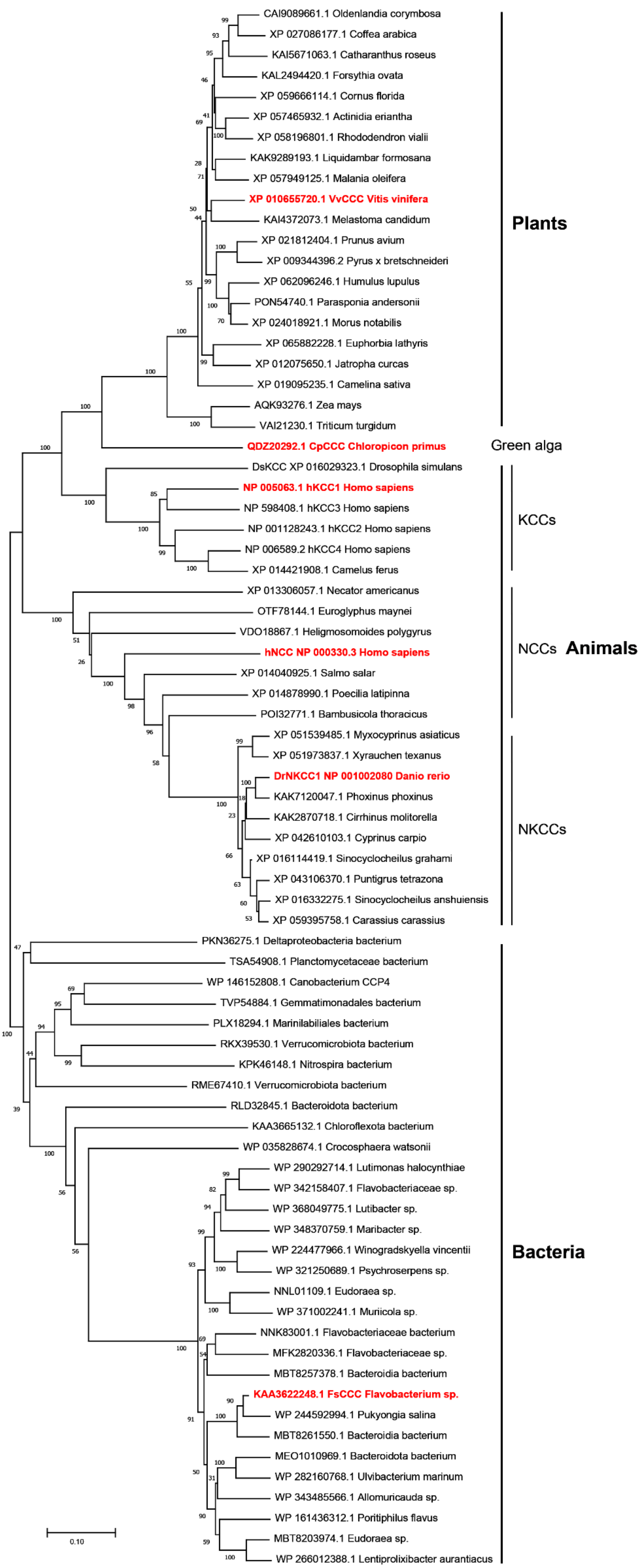
CAI9089661.1 Oldenlandia corymbosa
XP 027086177.1 Coffea arabica
KAI5671063.1 Catharanthus roseus
KAL2494420.1 Forsythia ovata
XP 059666114.1 Cornus florida
XP 057465932.1 Actinidia eriantha
XP 058196801.1 Rhododendron vialii
KAK9289193.1 Liquidambar formosana
XP 057949125.1 Malania oleifera
XP 010655720.1 VvCCC Vitis vinifera
KAI4372073.1 Melastoma candidum
XP 021812404.1 Prunus avium
XP 009344396.2 Pyrus x bretschneideri
XP 062096246.1 Humulus lupulus
PON54740.1 Parasponia andersonii
XP 024018921.1 Morus notabilis
XP 065882228.1 Euphorbia lathyris
XP 012075650.1 Jatropha curcas
XP 019095235.1 Camelina sativa
AQK93276.1 Zea mays
VAI21230.1 Triticum turgidum
Plants
QDZ20292.1 CpCCC Chloropicon primus
Green alga
DsKCC XP 016029323.1 Drosophila simulans
NP 005063.1 hKCC1 Homo sapiens
NP 598408.1 hKCC3 Homo sapiens
NP 001128243.1 hKCC2 Homo sapiens
NP 006589.2 hKCC4 Homo sapiens
XP 014421908.1 Camelus ferus
KCCs
XP 013306057.1 Necator americanus
OTF78144.1 Euroglyphus maynei
VDO18867.1 Heligmosomoides polygyrus
hNCC NP 000330.3 Homo sapiens
XP 014040925.1 Salmo salar
XP 014878990.1 Poecilia latipinna
POI32771.1 Bambusicola thoracicus
NCCs
Animals
XP 051539485.1 Myxocyprinus asiaticus
XP 051973837.1 Xyrauchen texanus
DrNKCC1 NP 001002080 Danio rerio
KAK7120047.1 Phoxinus phoxinus
KAK2870718.1 Cirrhinus molitorella
XP 042610103.1 Cyprinus carpio
XP 016114419.1 Sinocyclocheilus grahami
XP 043106370.1 Puntigrus tetrazona
XP 016332275.1 Sinocyclocheilus anshuiensis
XP 059395758.1 Carassius carassius
NKCCs
PKN36275.1 Deltaproteobacteria bacterium
TSA54908.1 Planctomycetaceae bacterium
WP 146152808.1 Canobacterium CCP4
TVP54884.1 Gemmatimonadales bacterium
PLX18294.1 Marinilabiliales bacterium
RKX39530.1 Verrucomicrobiota bacterium
KPK46148.1 Nitrospira bacterium
RME67410.1 Verrucomicrobiota bacterium
RLD32845.1 Bacteroidota bacterium
KAA3665132.1 Chloroflexota bacterium
WP 035828674.1 Crocosphaera watsonii
WP 290292714.1 Lutimonas halocynthiae
WP 342158407.1 Flavobacteriaceae sp.
WP 368049775.1 Lutibacter sp.
WP 348370759.1 Maribacter sp.
WP 224477966.1 Winogradskyella vincentii
WP 321250689.1 Psychroserpens sp.
NNL01109.1 Eudoraea sp.
WP 371002241.1 Muriicola sp.
NNK83001.1 Flavobacteriaceae bacterium
MFK2820336.1 Flavobacteriaceae sp.
MBT8257378.1 Bacteroidia bacterium
KAA3622248.1 FsCCC Flavobacterium sp.
WP 244592994.1 Pukyongia salina
MBT8261550.1 Bacteroidia bacterium
MEO1010969.1 Bacteroidota bacterium
WP 282160768.1 Ulvibacterium marinum
WP 343485566.1 Allomuricauda sp.
WP 161436312.1 Poritiphilus flavus
MBT8203974.1 Eudoraea sp.
WP 266012388.1 Lentiprolixibacter aurantiacus
Bacteria
0.10

Supplementary Figure 1. Plant, animal and bacterial CCC proteins (76 sequences) segregate in five clades, representing plant CCCs (including Protist green alga), animal KCCs, animal NCCs, animal NKCCs and bacterial CCCs. Bootstrapped cladogram of representative CCCs was inferred from 1000 replicates through the BLASTP search. Scale=substitution per site.

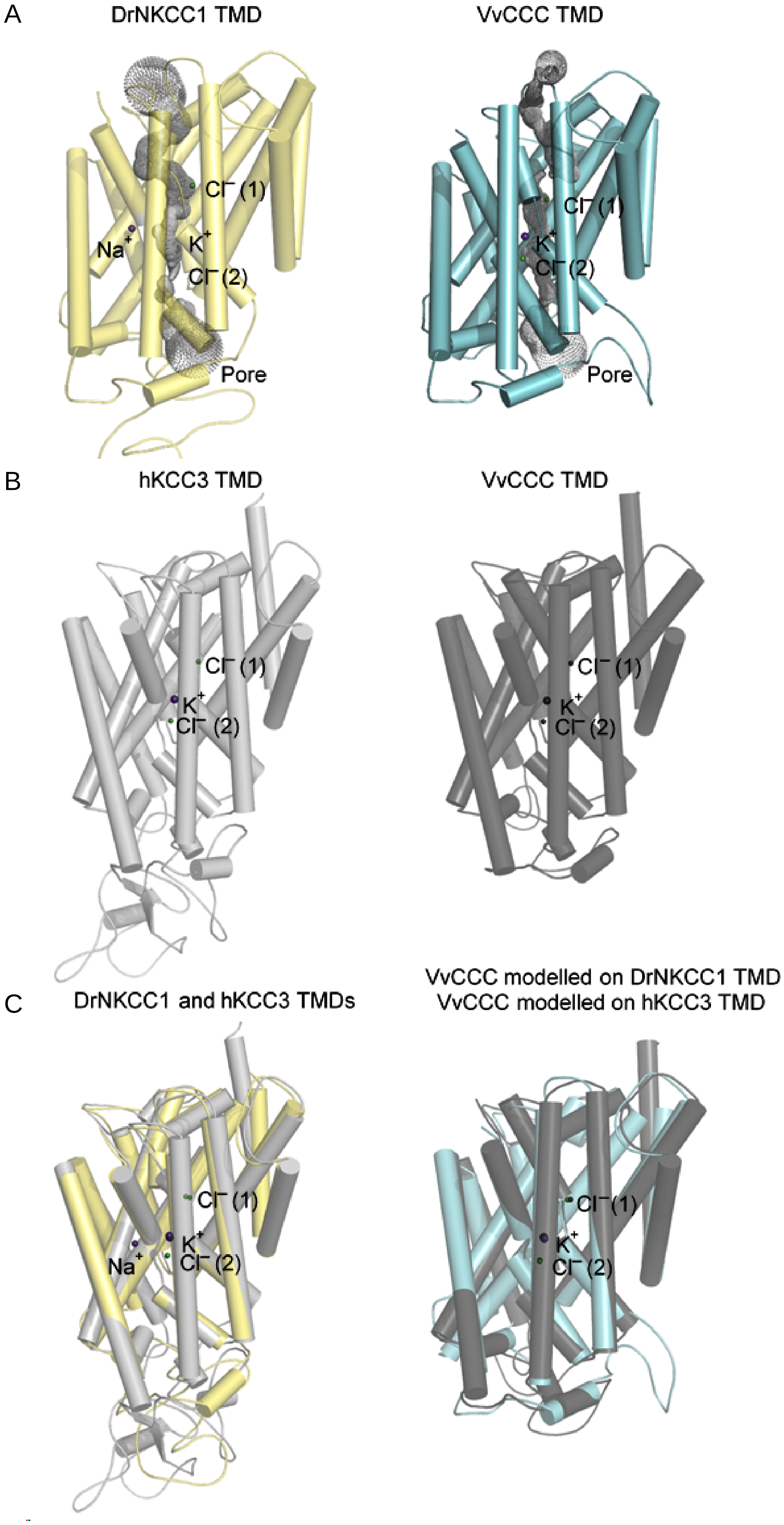
A
DrNKCC1 TMD
VvCCC TMD
Cl⁻ (1)
Na⁺
K⁺
Cl⁻ (2)
Pore
B
hKCC3 TMD
VvCCC TMD
C
DrNKCC1 and hKCC3 TMDs
VvCCC modelled on DrNKCC1 TMD
VvCCC modelled on hKCC3 TMD

Supplementary Figure 2. Comparison of VvCCC models using DrNKCC1 and hKCC3 proteins as templates.

(**A**) Cartoon representations of TMDs (chains B) of DrNKCC1 (yellow) structure, and VvCCC model (cyan) based on TMD template of DrNKCC1, illustrating poses of Na^+ (only in DrNKCC1), K^+ and two $Cl^–$ ions (cpk spheres) in pores.
(**B**) Cartoon representations of TMDs (chains B) of cryo-EM hKCC3 structure (light grey), and VvCCC (dark grey) models based on TMD template of hKCC3, illustrating poses of K^+ and two $Cl^–$ ions (cpk spheres) in pores.
(**C**) Superpositions of TMDs (chains B) of DrNKCC1 (yellow) and hKCC3 (grey) structures, and VvCCC model based on TMD templates of DrNKCC1 (cyan) or hKCC3 (dark grey). Respective root-mean-square-deviation values of superposed structures are 1.5 Å (472 and 544 residues), and 1.6 Å (460 and 472 residues).

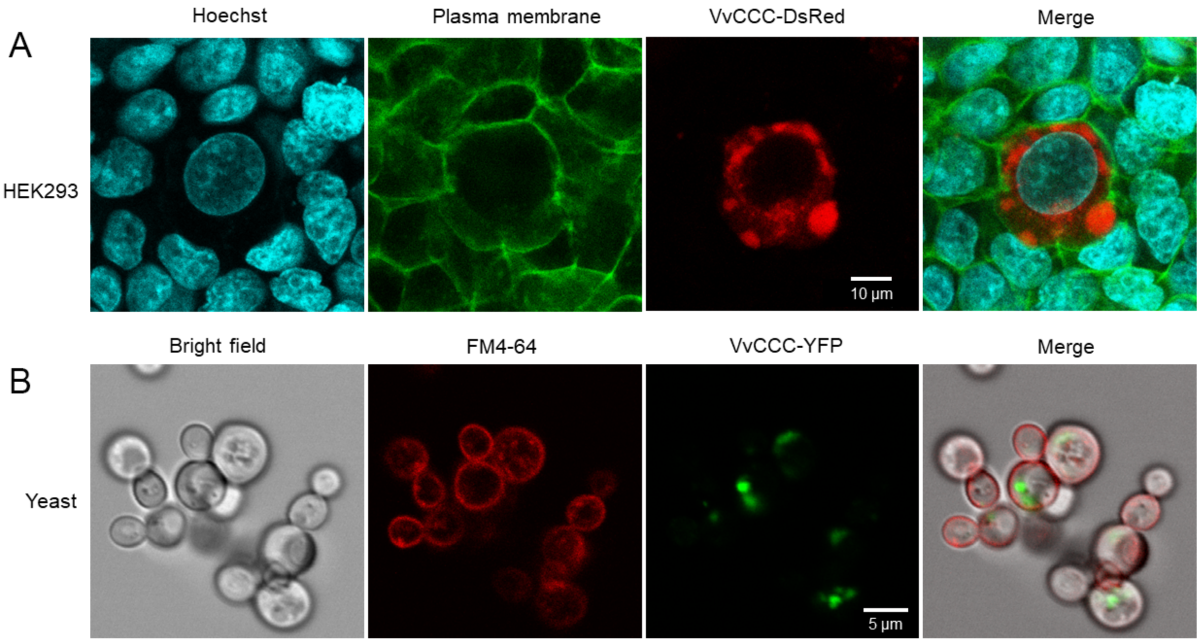

Supplementary Figure 3. Fluorescently tagged VvCCC localises to intracellular compartments of yeast and HEK293 cells.

(**A**) Transient expression of VvCCC-RFP in transfected HEK293 cells. Cells were counter stained with Hoechst nuclear dye and CellMask™ plasma membrane stain and imaged by confocal microscopy.
(**B**) Transient expression of VvCCC-YFP in *Saccharomyces cerevisiae* yeast. Cells were counter stained with FM4-64 as a membrane marker and imaged by confocal microscopy.

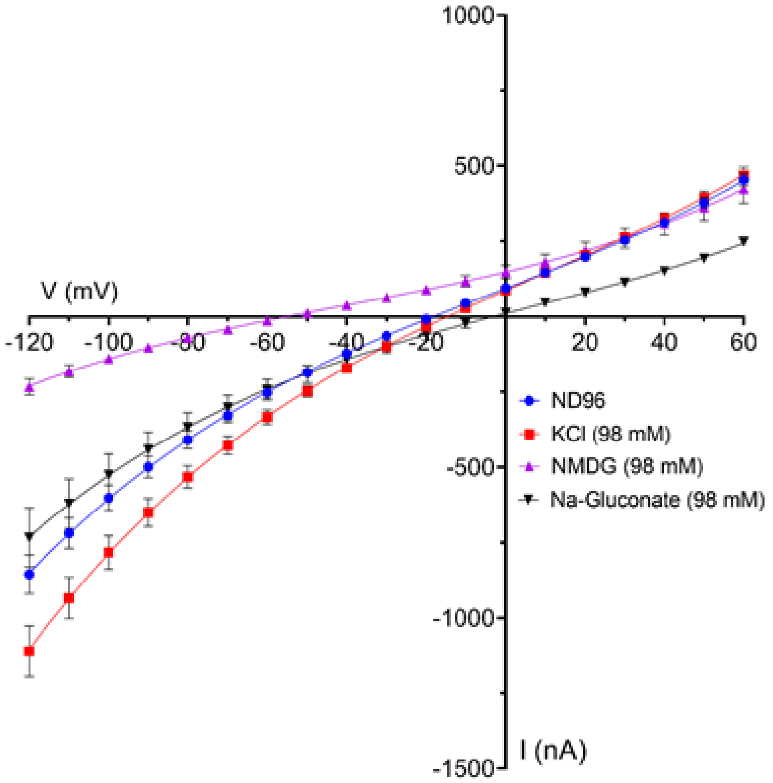

Supplementary Figure 4. Selectivity of *VvCCC*-injected *Xenopus* oocyte currents to K^+, Na^+ and Cl^-.

Current-voltage relationships of oocytes injected with VvCCC at the indicated membrane potential (V) while bathed in 96 mM NaCl and 2 mM KCl (ND96, blue), or the same solution where 96 mM NaCl and 2 mM KCl were replaced with 98 mM KCl (red), or 98 mM NMDG-Cl (purple), and the standard ND96 (black). Data are the mean ± Standard Error of the Mean (SEM, calculated in Microsoft Xcel 2019) of three oocytes after the subtraction of currents from a single water-injected control oocyte.

Supplementary Dataset 1

The list of 76 protein sequences of plant, animal and bacterial CCCs

```
>XP_010655720.1 VvCCC Vitis vinifera
MRNLKISMQGNMSSDAREESSTNHEELNGSERESKLELFGFDSLVNILGLKSMTGEAIAAPSSPRDGEDVSNTPGRSKAN
DLKLGTLMGVFVPCLQNILGIIYYIRFSWIVGMAGIGQSLLLVSFCGLCTFLTSISLSAIATNGAMKGGGPYYLIGRALG
PEVGVSIGLCFFLGNAVAGSLYVLGAVETFLDALPGAGIFGEVVTKVNGTEAAVAVPSPNLHDLQVYGIVVTIILCFIVF
GGVKMINRVAPAFLIPVLFSLFCIFVGAVLARKDHPAVGVTGLSLKSLKDNWSSSYQNTNNAGIPDPDGAVSWNFNALVG
LFFPAVTGIMAGSNRSASLRDTQRSIPVGTLAATLSTSAMYLFSVLLFGSLATREKLLTDRLLTATIAWPLPAIIYIGII
LSTLGAALQSLTGAPRLLAAIANDDILPVLHYFRVAEGSEPHIATLFTALICIGCVIIGNLDLITPTITMFFLLCYAGVN
LSCFLLDLLDAPSWRPRWKFHHWSLSLLGAVLCIVIMFLISWSFTVVSLALASLIYYYVCIKGKAGDWGDGFKSAYFQLA
LRSLRSLGASQVHPKNWYPIPLIFCRPWGKLPENVPCHPKLADFANCMKKKGRGMSIFVSILDGDYHECAEDAKTACRQL
STYIDYKRCEGVAEIVVAPSMSDGFRGIVQTMGLGNLKPNIVVMRYPEIWRRENLIEIPATFVGIINDCIVANKAVVIVK
GLDEWPNEYQRQYGTIDLYWIVRDGGLMLLLSQLLLTKESFESCKIQVFCIAEEDSDAEELKADVKKFLYDLRMHAEVIV
ISMKSWDAQGEGVSQQDESIEAFTGAQRRIAGYLSEMKEAAKREGTPLMADGKSVVVNEQQVEKFLYTTLKLNSTILRYS
RMAAVVLVSLPPPPLNHPAYFYMEYMDLLVENVPRLLMVRGYRRDVVTLFT
>KAK9289193.1 Liquidambar formosana
MDNGEIEGADDEFPPQGGRRYRPVVAHDRAVLEMSSLDPGSSSALPTPKPPLRNIKVNSQANMDSDTSEGSLPTQVEVNG
SQRESKLELFGFDSLVNILGLKSMTGEQIPAPSSPRDGEDISITLGRPKTNDVKLGTLMGVFVPCLQNILGIIYYIRFSW
IVGMAGIGESLLLVAFCGLCTFLTSISLSAIATNGAMKGGGPYYLIGRALGPEVGVSIGLCFFLGNAVAGALYVLGAVET
FLDAVPSAGIFRETITKVNGTTVPEPITSPSLHDLQIYGIVVTIVLCFIVFGGVKMINRVAPAFLIPVLFSFICIFAGIV
LARKDYPKAGITGLSLKSFKENWSSDYQKTNNAGIPDHEGAVDWNFNALVGLFFPAVTGIMAGSNRSASLKDTQRSIPVG
TLAATLTTSALYLFSVLLFGALATREKLLTDRLLTATVAWPFPAIIYIGIILSTLGAALQSLTGAPRLLAAIANDDILPV
LNYFKVADGSEPYIATIFTAFICIGCVLIGNLDLISPTITMFFLLCYAGVNLSCFLLDLLDAPSWRPRWKFHHWSLSLLG
ASLCIVIMFLISWSFTVVSLALASLIYYYVSIKGKAGDWGDGFKSAYFQLALRSLRSLGASQVHPKNWYPIPLIFCRPWG
KLPENVPCHPKLADFANCMKKKGRGMSIFFSILDGDYHECAEDAKAACKQLSMYLDYKRCEGVAEIVVAPNMSVGFRGIV
QTMGLGNLKPNIVVMRYPEIWRRENLTGIPATFVGIINDCIVANKAVVIVKGLDEWPNEYQRQYGTIDLYWIVRDGGLML
LLSQLLLTKESFESCKIQVFCIAEEDSDAEELKADVKKFLYDLRMQAEVIVITMKSWDAQVEGGSQQDESVEAFTGAQRR
IASYLAEMKEAAQREGTSLMADGKPVDVHEQQVEKFLYTTLKLNSTILRYSRMAAVVLVSLPPPPVNHPAYFYMEYMDLL
VENVPRLLIVRGYRKDVVTLFT
>XP_057949125.1 Malania oleifera
MEIQMDNGEIGEGGGGEISPQSGRKYRPVVAHDRAVLEMSSLDSSPSSSSASFSARETPLKKIKVASQANMGLDARKGSS
PTTTNASQRESKLELFGFDSLVNILGLKSMTGEQIPAPSSPRDGEDVSITLGRPKINDVKLGTMMGVFMPCLQNILGIIY
YIRFSWIVGMAGIGESLLLVSFCGLCTFLTSISLSAIATNGAMKGGGPYYLIGRALGPEVGVSIGLCFFLGNAVAGSLYV
LGAVETFLDAVPAAGIFRQTITKVNGTVVPEPIPAPSLHDLQIYGIVVTIILCFIVFGGVKIINRVAPAFLVPVLFSLFC
IFIGIFLARKDHPAAGITGLSLTTFKENWSSDYQITSDAGIPDPDGSIYWNFNALVGLFFPAVTGIMAGSNRSASLKDTQ
RSIPIGTLAATLSTSALYLISVLLFGALATREKLLTDRLLTATVAWPFPAIIYAGIILSTLGAALQSLTGAPRLLAAIAN
DDILPVLNYFKVVDGSEPHIATFFTAFICIGCVVIGNLDLITPTITMFFLLCYAGVNLSCFLLDLLDAPSWRPRWKFHHW
SLSLLGASLCIVIMFLISWSFTVVSLALASLIYYYVSIKGKAGDWGDGFKSAYFQLALRSLRSLGASQVHPKNWYPIPLI
FCRPWGKLPENVPCHPKLADFANCMKKKGRGMSIFVSILDGDYHECAEDAKAACRQLSTYIDYKRCEGVAEIVVAPSMSD
GFRGIVQTMGLGNLKPNIVVMRYPEIWRRENLSEIPATFVGIINDCIVANKAVVIVKGLDEWPNEYQKQYGTIDLYWIVR
DGGLMLLLSQLLLTKESFESCKIQVFCIAEEDSDAEELKTDVRKFLYDLRMQAEVIVITMKSWDAQVEGGSQPDESVEAF
MGAQQRIAHYLADIKEKAERERTPLMADGKPVVVNEQQVEKFLYTTLKLNSTILRYSRMAAVVLVSLPPPPLSHPAYFYM
EYMDLLVENVPRLLIVRGYRRDVVTLFT
>KAL2494420.1 Forsythia ovata
MASNNNNGEEIESSDDNEFSSGRGLGGRKYRPVFAHDHDRAVLEMSSIDPGDASSSSSLPVRNDLKKIKVGMQPNMTSEG
QDGSLPNHGGVNGAQTESKLELFGFDSLVNILGLKSMTGDQIPPPPSLRDGEDVTIPLEPPKTTDVKSGTMMGVFVPCLQ
NILGIIYYIRFSWIVGMAGIGESLLLVAFCGSCTFLTTISLSAIATNGAMKGGGPYYLIGRALGPEVGVSIGLCFFLGNA
VAGALYVLGAVETFLNAVPSAGLFRETITKVNGTAVAEPITSPSLHDLQIYGIIVTIILCFIVFGGVKMINRVAPAFLIP
VLFSLVCIFMGILLARKDKPAVGITGLSLKSFKENWSSDYQNTNNAGIPDPDGKIYWNFNALVGLFFPAVTGIMAGSNRS
ASLKDTQRSIPIGTLAATLTTSVLYIISVLFFGALATREKLLTDRLLTATVAWPFPAIIYIGIILSTLGAALQSLTGAPR
LLAAIANDDILPVLNYFRVADGSEPHVATFFTAFLCIGCVVIGNLDLITPTVTMFYLLCYAGVNLSCFLLDLLDAPSWRP
RWKFHHWSLSLLGASLCIVIMFLISWSFTVVSLALASLIYYYVSIKGKAGDWGDGFKSAYFQLALRSLRSLGASQVHPKN
WYPIPLVFCRPWGKLPENVPCHPKLADFANFMKKKGRGMSIFVSILDGDYHERAEDAKEACKALSTYIDYKRCEGVAEIV
VAPSMSDGFRGIVQTMGLGNLKPNIVVMRYPEIWRRENLTEIPATFVGIINDCIVANKAVVIVKGLDEWPNEYQRQYGTI
DLYWIVRDGGLMLLLSQLLLTKESFESCKIQVFCIAEEDSDAEELKADVRKFLYDLRMQAEVIVISMKSWDAKAEQQDES
VDAFTGAQQRISSYLAGMKERAQKEGTPLMADGKPVVLNEQQVEKFLYTTLKLNSTILKYSRMAAVVLVSLPPPPLNHPA
YFYMEYMDLLVENVPRLLIVRGYRRDVVTLFT
>XP_059666114.1 Cornus florida
MMDNGEIESSDDNEVETSQTRGRKYRPVVAHDKDHAILEMSSLDPGSSSEPLPNRDTSLKKIKVSTQLNMGSDARESSLP
TDGGLNDSQRESKLELFGFDSLVNILGLKSMTGEQIPTPSSPRDGEDVSLALERPKATDVKLGTLMGVFVPCLQSILGII
YYIRFSWIVGMAGIGESLLLVSFCGLCTSLTTISLSAIATNGAMKGGGPYYLIGRALGPEVGVSIGLCFFLGNAVAGALY
VLGAVETFLNAVPGAGIFKETITKVNGTAVAEPISSPNLHDLQIYGIIVTVVLCFIVFGGVKMINRVAPAFLVPVLFSLF
SIFVGILLARKDQPTTGITGLSLDTFKGNWSPDYQTTNNAGIPDPEGKIYWNFNALVGLFFPAVTGIMAGSNRSASLKDT
QRSIPVGTLAATLTTSAMYLFSVLLFGALATRDKLLTDRLLTATVAWPFPALIYIGIILSTLGAALQSLTGAPRLLAAIA
NDDILPVLNYFKVADGSEPHIATLFTAFLCIGCVIIGNLDLITPTVTMFFLLCYAGVNLSCFLLDLLDAPSWRPRWKIHH
WSLSLLGASLCIVIMFLISWSFTVVSLALVSLIYYYVSIKGKAGDWGDGFKSAYFQLALRSLRSLGANQVHPKNWYPIPL
IFCRPWGKLPENVPCHPKLADFANCMKKKGRGMSIFFSILDGDYHECAEDAKTACKQLSTYIDYKRCEGVAEIIVAPSMS
DGFRGIIQTIGLGNLKPNIVVMRYPEIWRRENLTEIPTTFVGIINDCIVANKAVVIVKGLDEWPNEYQRQYGTIDLYWIV
RDGGLMLLLSQLLLTKESFESCKIQVFCIAEEDSNAEELKADVKKFLYDLRMQAEVIVVTMKSWDVQVDSGAQQDESVEA
FTGAQRRIGKYLADMREKAQREGTQLMADGKPVVVNEQQVEKFLYTTLKLNSTILRYSRMAAVVLVSLPPPPVNHPAYFY
MEYMDLLVENVPRLLIVRGYRKDVVTLFT
>CAI9089661.1 Oldenlandia corymbosa
MVGNVEIEASDDNEFPPPSHGLGGRKYSPVVAHDSDRAVVEMSSLDSASSSSSTPFPNRNPLKVKLGNQANNASEVREGS
LPTHVRANGSQVDSKLELFGFDSLVNILGLKSMTGDPIPAPSSPRDGDDATINLGRPRDTGVKLGTMMGVFVPCLQNILG
IIYYIRFSWIVGMAGIGESLLLVAFCGSCTFLTTISLSAIATNGAMKGGGPYYLIGRALGPEVGVSIGLCFFLGNAVAGA
LYVLGAVETFLNAVPSAGIFKETITRVNGTEVAQPITSPSLHDLQIYGIVVTIILCFIVFGGVKIINRVAPAFLIPVLFS
LVCIFAGIFLARKDHPAAGITGLSLDSFKDNWSSDYQTTNNAGIPDPDGKIYWNFNALVGLFFPAVTGIMAGSNRSASLK
DTQRSIPVGTLAATLTTSVLYLVTVLFFGALATREKLLTDRLLTATVAWPFPAIIYIGIILSTLGAALQSLTGAPRLLAA
IANDDILPVLNYFKVADGGEPHIATFFTAFLCIGCVVIGNLDLITPTITMFYLLCYAGVNLSCFLLDLLDAPSWRPRWKF
HHWSLSLVGALLCIVIMFLISWAFTVVSLALASLIYYYVSIKGKAGDWGDGFKSAYFQLALRSLRSLGADQVHPKNWYPI
PLVFCRPWGKLPENVPCHPKLADFANCMKKKGRGMSIFFSIIDGDYHECAEDAKVACKQLSTYIDYKQCEGVAEIVVAPS
MSDGFRGIIQTMGLGNLKPNIVVMRYPEIWRRENLIEIPATFVGIINDCIVANKAVVIVKGLDEWPNAYQRQYGSIDLYW
IVRDGGLMLLLSQLLLTKESFESCKIQVFCIAEEDSDAEELKADVKKFLYDLRMQAEVIVISMKSWDAQAEQQDESFEAF
TGAQQRISSYLAGIKERAQREGTSLMADGKPVVVNEQQVENFLYTTLKLNSTILKYSRMAAVVLVSLPPPPLNHPSYFYM
```

EYMDLLVENVPRLLIVRGYRRDVVTLFT
>XP_057465932.1 Actinidia eriantha
MDNGEIEGSDDNEFASPQGVRGGRKYRPVVAHDNDRAVVEMSSLDPGSSSSASFPNRESSLKKIKVSSQPNLGSDAREGS
LPTHVGTNGPQKESKLELFGFDSLVNILGLKSMTGEQIPAPSSPRDGEDVSMTLERPKTTGLKLGTMMGVFVPCLQNILG
IIYYIRFSWIVGMGGIGESLLLVAFCGSCTFLTAISLSAIATNGAMKGGGPYYLIGRALGPEVGVSIGLCFFLGNAVAGA
LYVLGAVETFLKAVPSAGIFRETVTKVNGTAVAEPISSPSLHDLQVYGIVVTIILCFIVFGGVKMINRVAPAFLIPVLLS
IFCIFVGIVAARKDDPAIGITGLSLGTFKDNWSSEYQPTNNAGIPDPEGKSIWNFNALVGLFFPAVTGIMAGSNRSASLK
DTQRSIPVGTLAATLTTSGLYLISVLLFGALATREKLLTDRLLTATVAWPFPAIVYVGIILSTLGAALQSMTGAPRLLAA
IANDDILPVLNYFKVADGTEPYVATLFTAFICIGCVIIGNLDLITPTITMFFLLCYAGVNLSCFLLDLLDAPSWRPRWKF
HHWSLSLLGASLCIVIMFLISWSFTVVSLALASLIYYYVSIKGKAGDWGDGFKSAYFQLALRSLRSLGANQVHPKNWYPI
PLVFCRPWGKLPENVPCHPKLADFANCMKKKGRGMSIFVSILDGDYHECAEDAKTACKQLSTYIDYKRCEGVAEIVVAPS
MSDGFRGIIQTMGLGNLKPNIVVMRYPEIWRRENLTEIPATFVGIINDCIVANKAVVIVKGLDEWPNEYQRQYGTIDLYW
IVRDGGLMLLLSQLLLTKESFESCKIQVFCIAEEDSDAEGLKADVKKFLYDLRMQAEVIVVTMKSWDVQMEGGPQQDESV
EAFTGAQQRISSYLAGMKEKAQKDGAPLLADGKPVVVNEQQVEKFLYTTLKLNSTILRYSRMAAVVLVSLPPPPLNHPAY
FYMEYMDLLVENVPRLLMVRGYRRDVVTLFT
>XP_065882228.1 Euphorbia lathyris
MENEDVEGGMEDEFRSPGRKYRPVVAHDRAVLEMSSMDHGSSSNADASRMAKVGPREHSNANQAAVPANGNVNGSESQHK
LELFGFDSLVNILGLKSMTAEQVVAPSSLSTEGDDGPHPYERPRVEEIKLGTMMGVFIPCLQSILGIIYYIRFTWIVGMA
GIGESLMLVTLCGLCTFLTSISLSAIATNGAMKGGGPYYLIGRALGPEVGVSIGLCFFLGNAVAGALYVLGAVETFLKAV
PAAGIFRETITHINGTRAVEPIESPSSHDLQIYGIVVTIVLCFIVFGGVKMINRVAPAFLIPVLFSLVCIFIGVLLARKD
HPATGITGLSLKSFQDNWGPAYQLTNNAGIPDPKGEIYWNFNALVGLFFPAVTGIMAGSNRSASLKDTQRSIPVGTLAAT
LTTSAMYVISVVFFGAVATRDKLLTDRLLTATIAWPLPAIVYVGIILSTLGAALQSLTGAPRLLAAIANDDILPVLNYFR
VADGHEPHIATLFTSFICIGCVVIGNLDLITPTVTMFFLLCYAGVNLSCFLLDLLDAPSWRPRWKFHHWSLSLLGALLCI
VIMFLISWSFTVVSLALASLIYYYVSIKGKAGDWGDGFKSAYFQLALRSLRSLGANQVHPKNWYPIPLVFCRPWGKLPEN
VPCHPKLADFANCMKKKGRGMSIFVSILDGDYHEHAEAAKLACKQLSTYIDYKSCEGVAEIVVAPSMSEGFRGIVQTMGL
GNLKPNIVVIRYPEIWRRENLTEIPVTFVGIINDCIVANKAVVIVKGLDEWPNMYQKQYGTIDLYWIVRDGGLMLLLSQL
LLTKESFESCKIQVFCIAEEDSDAEELKADVKKFLYDLRMQAEVIVISMKSWDLQAEGGQPDESLEAFTAAQRRIGSYLG
EMKSRAQELGTSLMADGKPVIVNEQQVEKFLYTTLKLNSTILRHSRMASVVLVSLPPPPVNHPAYFYMEYMDLLVQNVPR
LLIVRGYRRDVVTLFT
>XP_012075650.1 Jatropha curcas
MDNEDVEGGMEDDFQGQHGRKYRPVVAHDRAVLEMSSMDPGSSSSPGTQSSNSKKVKLVVPEDMHANAPQVRIPANGEVH
GSESEHKLELFGFDSLVNILGLKSMSTEQVVAPSSPRDGEDGNVTYERPRANEVKLGTMMGVFVPCLQNILGIIYYIRFT
WIVGMAGIGESLLLVAFCGLCTFLTSISLSAIATNGAMKGGGPYYLIGRALGPEVGVSIGLCFFLGNAVAGALYVLGAVE
TFLKAVPVAGIFRETVTQVNGTATAEPIESPSAHDLQIYGIVVTLLLCFIVFGGVKMINRVAPAFLIPVLFSLFCIFVGT
FTARKDRPAAGITGLSLESFKENWSSDYQFTNDAGIPDPQGKTYWNFNALVGLFFPAVTGIMAGSNRSASLKDTQRSIPV
GTLAATLSTTVLYLISVLFFGALATRDKLLTDRLLTATIAWPAPAIVYIGIILSTLGAALQSLTGAPRLLAAIANDDILP
VLNYFKVADGQEPHIATLFTAFICIACVVIGNLDLITPTVTMFFLLCYAGVNLSCFLLDLLDAPSWRPRWKFHHWSLSLL
GASLCIVIMFLISWSFTVVSLALASLIYYYVSVKGKAGDWGDGFKSAYFQLALRSLRSLGANQVHPKNWYPIPLIFCRPW
GKLPENVPCHPKLADFANCMKKKGRGMSIFVTILDGDYREHAEDAKVACKQLATYIDYKNCEGVAEIVVAPNMFEGFRGI
VQTMGLGNLKPNIVVMRYPEIWRRENLTEIPATFVGIINDCIVANKAVVIVKGLDEWPNEYQRQYGTIDLYWIVRDGGLM
LLLSQLLLTKESFESCKIQVFCIAEEDSDAEELKADVKKFLYDLRMQAEVIVISMKSWDIRVQDGSQQDESFEAFTAAQR
RISNYLHDMKAKAQGEGTTLMADGKPVVVNEQQVEKFLYTTLKLNSTILRYSRMAAVVLVSLPPPPVDHPSYFYMEYMDL
LVENVPRLLMVRGYRRDVVTLFT
>XP_021812404.1 Prunus avium
MDNADVEAGGEDEFHGKSGRKYRPVVDDDRAVLEMSSMDPSSSSSSSSALPVHQASLKTIKVGTQENMGSDAKEGHPPTH
VQANGPQRESKLELFGFDSLVNILGLKSMTDEQSAAPSSPRDGEDISITQGRPKPTGVKLGTLMGVFVPCLQNILGIIYY
IRFSWIVGMAGIAESLFLVSFCGLCTFLTAISLSAIATNGAMKGGGPYYLIGRALGPEVGVSIGLCFFLGNAVAGSLYVL
GAVETFLKAVPAAGLFRETTRVNGTSVAIQSPSSHDLQIYGIVVTIILCFIVFGGVKMINRVAPAFLIPVLLSLFCIYIG
IALARKNYPVDGVTGLSLNSFKDNWNSDYQKTNNAGIPDPDGKVSWNFNAMVGLFFPAVTGIMAGSNRSASLRDTQRSIP
IGTLAATLSTTAMYLVSVLLFGALASRQKLLTDRLLTATIAWPFPAFIYIGIILSTLGAALQSLTGAPRLLAAIANDDIL
PVLNYFKVSDGSEPNIATLFTALLCIGCVVIGNLDLITPTITMFFLLCYAGVNLSCFLLDLLDAPSWRPRWKFHHWSLSL
LGALLCIVIMFLISWSFTVVSLALASLIYYYVSIKGKAGDWGDGFKSAYFQLALRSLRSLGANQVHPKNWYPIPLIFCRP
WGKLPENVPCHPKLADFANCMKKKGRGMSIFFSILDGDYRECAEDAKAACKQLATYLDYKNCEGVAEIVVAPSMSEGFRG
IVQTMGLGNLKPNIVVMRYPEIWRRENLTEIPATFVEIINDCIVANKAVVIVKGLDEWPNEYQRQYGTIDLYWIVRDGGL
MLLLSQLLLTKESFESCKIQVFCIAEEDTDAEGLKADVKKFLYDLRMHAEVIVVTMKSWDVQADSGSPQDESVDAFSGAH
QRIANYMADMKAASEKQGTPLMADGKPVVVDEQQVEKFLYTTLKLNSTILRYSRMAAVVLVSLPPPPANHPAYFYMEYMD
LLVENVPRLLIVRGYRKDVVTLFT
>KAI4372073.1 Melastoma candidum
MDNGDVDGVVDEEYHQQGRRKYRPVVANDSAVVEMSSIDPGSSYPPSSSSAAPPPPPPPLRNIKVGQHANGVDLEEGTSPA
HPTANGPEKESRLELFGFDSLVNILGLKSMTGEQIAAPSSPRDGEDVSITSGHRKMEELKLGTMMGVFVPCLQNILGIIY
YIRFSWIVGMGGIGGSLLLVSFCGLCTFLTSISLSAIATNGAMKGGGPYYLIGRALGPEVGVSIGLCFFLGNAVAGALYV
LGAVETFLKAVPAAGIFRETVTRVNGTDITSIESPSSHDLQIYGIVVTIVLCFIVFGGVKMINRVAPAFLIPVLLSIFCI
FVGITLARMDKPTEGITGLRLSTFRDNWGSAYQSTNNAGIPDPDGKVQWNFNALVGLFFPAVTGIMAGSNRSASLKDTQQ
SIPVGTLSATLATTALYLISVLFFGALATREKLLTDRLLTATVAWPLPAIVYIGIILSTLGAALQSLTGAPRLLAAIAND
DILPILKYFRVAEGNEPHIATLFTALLCVGCVIIGNLDLITPTITMFFLLCYAGVNLSCFLLDLLDAPSWRPRWKFHHWS
LSLLGASLCIVIMFLISWSFTVVSLALASLIYYYVSVKGKAGDWGDGFKSAYFQLALRSLRSLGASQVHPKNWYPIPLIF
CRPWGKLPENVPCHPKLADFANCMKKKGRGMSIFVSILDGDYHECAEDAKAACKQLDTYIDYKNCEGVAEIVVAPSMSEG
FRGIVQTMGLGNLKPNIVVMRYPEIWRRENLTEIPATFVGIINDCIVANKAVVIVKGLDEWPNEYQRQYGTIDLYWIVRD
GGLMLLLSQLLLTKESFESCKIQVFCIAEEDSDAEGLKADVKKFLYDLRMQAEVIVITMKSWDVVENRAQHEESLEAFTA
AQKRITDYLSGMKEAAKREGTQLMADGKPVVVNEQQVEKFLYTTLKLNSTILRYSRMAAVVLVSLPPPPLNHPAYFYMEY
MDLLVENVPRLLMVRGYRRDVVTLFT
>PON54740.1 Parasponia andersonii
MDNGDIESGAEENFHAPLGRKYRPVVDNDSAVLEMSPMDPPGSSSSSSSALPVVRQAPLKKVKVSSRENLDSTVDEEANG
PQRESKLELFGFDSLVNILGLKSMTEEHSAEPSSPRDGENISITHGRPKASEVKLGTLMGVFVPFLQNILGIIYYIRFSW
IVGMAGIGESLLLVAFCGLCTFLTGISLSAIATNGAMKGGGPYYLIGRALGPEVGVSIGLCFFLGNAVAGSLYVLGAVET
FLKAVPAAGIFRETVTRVNGTVVPEPIQSPSSHDLQIYGIVVTIILCFIVFGGVKMINRVAPAFLIPVLFSLFCIFIGIA
VARKDHPTAGITGLSLESFKENWSPDYQNTNNAGIPDPEGKVSWNFNAMVGLFFPAVTGIMAGSNRSASLKDTQRSIPIG
TLAATLTTTAMYLVSVLLFGALATREKLLTDRLLTATVAWPFPAIIYIGIILSTLGAALQSLTGAPRLLAAIANDDILPV
LNYFKVAEGNEPHIATLFTAFLCIGCVIIGNLDLITPTITMFFLLCYAGVNLSCFLLDLLDAPSWRPRWKFHHWSLSLLG
ASLCIVIMFLISWSFTVVSLALASLIYYYVSIKGKAGDWGDGFKSAYFQLALRSLRSLGANQVHPKNWYPIPLIFCRPWG
KLPENVPCHPKLADFANCMKKKGRGMSIFVSILDGDYHECAEDAKEATKQLATYIDYKRCEGVAEIVVAPNMSEGFRGIV
QTMGLGNLKPNIVVMRYPEIWRRENLTEIPATFVGIINDCIVANKAVVIVKGLDEWPNEYQRQYGTIDLYWIVRDGGLML
LLSQLLLTKESFESCKIQVFCIAEEDVDAEALKADVKKFLYDLRMQAEVVVVNMKSWDAQAEGGGHQDESIEAFTSAQQR
IGSYLADMKSRAESQGAQLMADGKPVVVNEQQVEKFLYTTLKLNSTILRYSRMAAVVLVSLPPPPVNHPSYFYMEYMDLL
VENVPRLLIVRGYRRDVLTLFT
>XP_009344396.2 Pyrus x bretschneideri
MDNVDVEAGAENEFRGQRGRKYRPVVDNDRAVLEMSPMDPSSSSSSSSSLPVHQASLKKIKVSTQENMGSNINEGPSTQV
QDKGPQKESKLELFGFDSLVNILGLKSMTDEQTAAPSSPRDGEDIAITQGRPKPSDLKLGTLMGVFVPCLQNILGIIYYI

RFSWIVGMAGIAESLFLVSFCGLCTFLTAISLSAIATNGAMKGGGPYYLIGRALGPEVGVSIGLCFFLGNAVAGSLYVLG
AVETFLKAVPAAGIFKETTRVNGTSIAIQSPSSHDLQIYGIVVTILLCFIVFGGVKMINRVAPAFLIPVLFSLFCIYIGI
ALARKNHPVDGVTGLSSHSFKENWGSDYKKTNNNGIPDPDGKVSWNFNAMVGLFFPAVTGIMAGSNRSASLKDTQRSIPI
GTLAATLSTTAMYLVSVLLFGALATREKLLTDRLLSARIAWPFPVFIYIGIILSTLGAALQSLTGAPRLLAAIANDDILP
VLNYFRVSDGNEPHIATLFTALLCIGCVVIGNLDLITPTITMFFLLCYAGVNLSCFLLDLLDAPSWRPRWKFHHWSLSLL
GATLCIVIMFLISWSFTIVSLALASLIYYYVSIKGKAGDWGDGFKSAYFQLALRSLRSLGANQVHPKNWYPIPLIFCRPW
GKLPENVPCHPKLADFANCMKKKGRGMSIFFSILDGDYRECAEDAKTACKQLATYLDYKNCEGVAEIVVAPSMSEGFRGI
VQTMGLGNLKPNIVVMRYPEIWRRENLTEIPATFVEIINDCIVANKAVVIVKGLDEWPNEYQRQYGTIDLYWIVRDGGLM
LLLSQLLLTKESFESCKIQVFCIAEEDTDAEGLKFDVKKFLYDLRMHAEVIVVTMRSWDVQADGGSPQDESVEAFTGARR
RIADYMANMKAIAEKQGTPLMADGKQVFVDEQQVEKFLYTTLKLNSTILRYSRMAAVVLVSLPPPPANHPAYFYMEYMDL
LVENVPRLLMVRGYRRDVVTLFT
>XP_058196801.1 Rhododendron vialii
MDNGEIEGSDDNEFASSSSSKGVGLGVGRKYRPVVAHDTDRAVLEMSSLDPSSFPKKIKVSTQPNLGSDSTERSLPTHVG
ANGSQRESKLELFGFDSLVNILGLKSMTGEQIPAPSSPRDGEDVSTTLERPKITGVKSGTMMGVFVPCLQNILGIIYYIR
FSWIVGMGGIGESLLLVAFCGACTFLTSISLSAIATNGAMKGGGPYYLIGRALGPEVGVSIGLCFFLGNAVAGALYVLGA
VETFLKAVPSAGIFRETLTNVNATGVAEPIPSPSLHDLQVYGIVVTIIICFIVFGGVKMINRVAPAFLIPVLLSIFCIFI
GIAAAKKDHPASGITGLSLQTFKDNWSSDYQRTNSAGIPDPAGKTDWNYNALVGLFFPAVTGIMAGSNRSASLKDTQRSI
PIGTLAATLTTSGLYIVSVLLFGALATREKLLTDRLLTATVAWPFPLIVYVGIILSTLGAALQSMTGAPRLLAAIANDDI
LPVLNYFKVADGSEPHIATLFTAFICIGCVIIGNLDLITPTVTMFFLLCYTGVNLSCFLLDLLDAPSWRPRWKFHHWSLS
LIGASLCIVIMFLISWSFTVVSLALASLIYYYVSIKGKAGDWGDGFKSAYFQLALRSLRSLGANQVHPKNWYPIPLVFCR
PWGKLPENVPCHPKLADFANCMKKKGRGMSIFVSILDGDYYECAEDAKAACKQLSTYIDYKRCEGVAEIVVAPNMSDGFR
GIVQTMGLGNLKPNIVVMRYPEIWRRENLIEIPATFVGIINDCIVANKAVVIVKGLDEWPNEYQRQYGTIDLYWIVRDGG
LMLLLSQLLLTKESFESCKIQVFCIAEEDSDAEELKADVKKFLYDLRMQAEVFVVSMKTWDVHVEGGPQQDESVEAFTGA
QRRISSYLLGMKEKAEREKTPLMADGKAVVVNEQQVEKFLYTTLKLNSTILRYSRMAAVVLVSLPPPPVNHPAYFYMEYM
DLLVENVPRLLMVRGYRRDVVTLFT
>KAI5671063.1 Catharanthus roseus
MQQRRGDRRWLRRGELRKIKVGKQPNMASEEIEGSLPTHEGVNGSQTESKLELFGFDSLVNILGLKSMAGDPIPAPSSPR
DGDDATTNLGRPRDTAVKLGTMMGVFIPCLQNILGIIYYIRFSWIVGMAGIGESLLLVAFCGSCTFLTTISLSAIATNGA
MKGGGPYYLIGRALGPEVGVSIGLCFFLGNAVAGAMYVLGAVETFLNAVPGAGIFRETVTKVNGTEVATTANLHDLQVYG
VVVTIILCFIVFGGVKMINRVAPAFLIPVVVSLFCIFIGIFLAKKDYPAEGITGLSMKSFKENWASDYQTTNNAGIPDPN
GKQYWNFNALVGLFFPAVTGIMAGSNRSASLKDTQRSIPIGTLAATLATSGLYLVTVLFFGALATRDKLLTDRLLTATVA
WPFPALIYIGIILSTLGAALQSLTGAPRLLAAIANDDILPVLNYFKVADGNEPHIATLFTALLCIGCVVIGNLDLITPTI
TMFYLLCYAGVNLSCFLLDLLDAPSWRPRWKFHHWSLSLVGALLCIVIMFLISWAFTVVSLALATLIYYYVSLKGKAGDW
GDGFKSAYFQLALRSLRSLGADQVHPKNWYPIPLVFCRPWGRLPENVPCHPKLADFANCMKKKGRGMSIFVSIIDGDYHE
CAEDAKAACKQLSTYIDYKQCEGVAEIVVAPSMSDGFRGIVQTMGLGNLKPNIVVMRYPEIWRRENLTEIPATFVDIIND
CIVANKAVVIVKGLDEWPNEYQRQYGSIDLYWIVRDGGLMLLLSQLLLTKESFENCKIQVFCIAEEDSDAEGLKADVKKF
LYDLRMHAEVIVISMKSWDPKAEQQDESYEAFTGAQHRIADYMSRIKDKAQKEGTTTLMADGKPMVVNEQQVEKFLYTTL
KLNSTILKYSRMAAVVLVSLPPPPINHPAYFYMEYMDLLVENVPRLLIVRGYHRDVVTLFT
>XP_027086177.1 Coffea arabica
MAGNGEIEVSDEKEFSSSSSSSSQGLGHGRNYRPVVASDNDRAIVEMSSLESAAAASSSSSSSPFPNRNPMKVKASNQTN
MASEEREGSLPTRAHANGNQKDSKLELFGFDSLVNILGLKSMTEDQTPAPSSPRDGDDGTINLERPRVLDTGVKLGTMMG
VFVPCLQNILGIIYYIRFSWIVGMGGIGESLLLVAFCGSCTFLTTISLSAIATNGAMKGGGPYYLIGRALGPEVGVSIGL
CFFLGNAVAGALYVLGAVETFLNAVPSAGIFKETVTRVNGTEVAQPIASPSLHDLQIYGIVVTIILCFIVFGGVKMINRV
APAFLVAVLFSLFCIFIGILLARKDHPAAGITGLSLESFKENWSSDYQTTNNAGIPDPDGKIDWNFNALVGLFFPAVTGI
MAGSNRSASLKDTQRSIPVGTLAATLTTTGLYLISVLFFGALATREKLLTDRLLTATIAWPFPAIIYIGIILSTLGAALQ
SLTGAPRLLAAIANDDILPVLNYFKVADGNEPHIATFFTAFLCIGCVVIGNLDLITPTITMFYLLCYAGVNLSCFLLDLL
DAPSWRPRWKFHHWSLSLVGALLCIVIMFLISWAFTVVSLALASLIYYYVSIKGKAGDWGDGFKSAYFQLALRSLRSLGA
DQVHPKNWYPIPLVYCRPWGKLPENVPCHPKLADFANCMKKKGRGMSIFLSIMDGDYHECAEDAKIACKQLSTYIDYKQC
EGVAEIVVAPSMTEGFRGIVQTMGLGNLKPNMVIMRYPEIWRRENLTEIPASFVGIINDCIVANKAVVIVKGLDEWPNEY
QRQYGSIDLYWIVRDGGLMLLLSQLLLTKESFESCKIQVFCIAEEDSDAEELKADVRKFLYDLRMQAEVIVISMKSWDAQ
AEQQDESFEAFTGAQQRISNYLAGIKENAHREGTALMADGKPVVVNEQQVEKFLYTTLKLNSTILKYSRMAAVVLVSLPP
PPVNHPAYFYMEYMDLLVENVPRLLIVRGYRRDVVTLFT
>XP_024018921.1 Morus notabilis
MDNGDIEGGPEDNFHAPIGRKYRPVVDNDSAVLEMSSIDPSGSSSSSSALPLPQAPLKKVKVSSQENMDSTVNEEMNGPQ
RESKLELFGFDSLVNILGLKSMTEEHSAEPSSPRDGENISITHGRPKTSDIKLGTMMGVFVPCLQNILGIIYYIRFSWIV
GMAGIGQSLLLVAICGLCTFLTGISLSAIATNGAMKGGGPYYLIGRALGPEVGVSIGLCFFLGNAVAGALYVLGAVETFL
KAVPAAGIFRETITRVNVTVVAEPIQSPSSHDLQIYGIVVTILLCFIVFGGVKMINRVAPAFLIPVLFSLFCIYIGIAAA
GKDHPATGITGLSLESFKENWSSDYQNTNNAGIPDPDGEVSWSFNAMVGLFFPAVTGIMAGSNRSASLKDTQRSIPIGTL
SATLTTTAMYLVSVLLFGALATREKLLTDRLLTATVAWPFPAIIYIGIILSTLGAALQSLTGAPRLLAAIANDDILPVLN
YFKVSEGNEPHIATLFTAFLCIGCVIIGNLDLITPTITMFFLLCYCGVNLSCFLLDLLDAPSWRPRWKFHHWSLSLLGAS
LCIVIMFLISWSFTVVSLALASLIYYYVSIKGKAGDWGDGFKSAYFQLALRSLRSLGANHVHPKNWYPIPLIFCRPWGKL
PENVPCHPKLADFANCMKKKGRGMSIFVSILDGDYHECAEDAKEACKQLGTYIDYKRCEGVAEIVVAPNMSEGFRGIVQT
MGLGNLKPNIVVMRYPEIWRRENLTEIPATFVGIINDCIVANKAVVIVKGLDEWPNEYQRQYGTIDLYWIVRDGGLMLLL
SQLLLTKESFESCKIQVFCIAEEDVDAEALKADVKKFLYDLRMQAEVIVINMKSWDAQAEGGSHQDESIEAFTGAQQRIA
NYLADMKSRAEREGTQLMADGKPVVVNEQQVEKFLYTTLKLNSTILRYSRMAAVVLVSLPPPPVNHPAYFYMEYMDLLVE
NVPRLLIVRGYRRDVVTLFT
>XP_062096246.1 Humulus lupulus
MDNGDIESNSDDNLRAPIGRKYRPVVDNDSAVLEMSPMDPSGSSSSSSALPVVHQVPLKNVNVSSQRNMDTIVDEEAIRP
QRDSKLELFGFDSLVNILGLKSMTEEQSAEPSSPRDGENISITQGRPKTSELKLGTLMGVFVPCLQNILGIIYYIRFSWI
VGMAGIWESLLVVAFCGLCTFLTGISLSAIATNGAMKGGGPYYLIGRALGPEVGVSIGLCFFLGNAVAGSLYVLGAVETF
LKAVPAAGIFRETITRVNGTVIPEPIQSPSSHDLQIYGIVVTIVLCFIVFGGVKMINRVAPAFLIPVLFSLFCIFIGIFV
AKKDHPSPGITGLSLASFKENWGPDYQNTNNAGIPDPDGKVSWNFNAMVGLFFPAVTGIMAGSNRSASLKDTQRSIPIGT
LSATLTTTAMYVVSVLLFGALATREKLLTDRLLTATVAWPFPAIIYIGIILSTLGAALQSLTGAPRLLAAIANDDILPVL
NYFKVVEGNEPYIATLFTAFLCIGCVIIGNLDLITPTITMFFLLCYAGVNLSCFLLDLLDAPSWRPRWKFHHWSLSLLGA
FLCIVIMFLISWSFTVVSLALASLIYYYVSIKGKAGDWGDGFKSAYFQLALRSLRSLGANQVHPKNWYPIPLIFCRPWGK
LPENVPCHPKLADFANCMKKKGRGMSIFVSILDGDYHECAEDAKEATKQLATYIEYKRCEGVAEIVVAPNMSEGFRGIIQ
TMGLGNLKPNIVVMRYPEIWRRENLTEIPATFVGIISDCIVANKAVVIVKGLDEWPNEYQRQYGTIDLYWIVRDGGLMLL
LSQLLLTKESFESCKIQVFCIAEEDNDAEALKADVKKFLYDLRMQAEVIVINMKSWDGQTDGSAQPDESLGAYTGAQQRI
ANYMADMKSKAEREGTQLMADGKPVVVDEHQVEKFLYTTLKLNSTILRYSRMAAVVLVSLPPPPVNHPAYFYMEYMDLLV
ENVQRLLIVRGYRRDVVTLFT
>QDZ20292.1 CpCCC Chloropicon primus
KLGTLMGVFVPCLQNILGVILFIRLSWIVGQAGVLGSLGIVGMCCACTFLTSLSLSAIATNGHIKGGGPYYLIGRALGPE
LGVSVGICFYLGTAVAGAMYILGAVETILDMAPQLNIATESGATAIAKNDYRIYGFILLFFVVSVVFAGMKHLSRLAPAF
LAPVLLSVFFILVGIWSSNSRNLEGDGISGISMANLKDNWSPEYYDTDANGFPGHSSKFKTGASTVPWSFQTLLALFFPS
VTGIMAGSNRSASLENAQKSIPKGTLAAQLTTTTLYLSFVIWYGASAARDTLLDERLLSARISWPAPEIVSIGIILSTVG
AALQSLTGAPRLLQAIANDRILPGLNFLATKNDDDEPTMCLLVTFLVTGAAISAGQLDVITPIITMFFLLCYAGVNSSCA
LLGMMQAPNWRPRWRYYHWFLSVIGLVLCF
>DrNKCC1 NP_001002080 Danio rerio

MSASPPPFAAPGDYLSAPEPDALKPAGPTPSQSRFQVDLVSESAGDSQTLGTSDSSPPAYAAEPPSDGLRDSVAGGEEAK
GRFRVVNFAASSPDAAPAESAQNGDTVMSEGSLHSSTGGQQHHHYDTHTNTYYLRTFGHNTIDAVPNIDFYRQTAAPLGE
KLIRPTLSELHDELDKEPFEDGFANGEELTPAEEAAAKEASESKGVVKFGWIKGVLVRCMLNIWGVMLFIRMTWIVGQAG
IAYSCIIVIMATVVTTITGCSTSAIATNGFVRGGGAYYLISRSLGPEFGGSIGLIFAFANAVAVAMYVVGFAETVVELLI
NSGALMFDQINDVRIIGTITVILLLGISVAGMEWEAKAQIFLLVILITAIFNYFIGSFIPVESKEKFGFFSYDTGILADN
FGPDFRGETFFSVFSIFFPAATGILAGANISGDLADPQMAIPKGTLLAILITGLVYIGVAISAGACIVRDATGIESNITL
SGIVNCTDAACKFGYDFTSCRPTVEGEKSSCKFGLHNDFQVMSVVSGFSPLITAGIFSATLSSALASLVSAPKVFQALCK
DNIYPGITVFGKGYGKNNEPLRGYLLTFAIALAFILIAELNVIAPIISNFFLASYALINFSVFHASLANSPGWRPSFKYY
NMWVSLAGASLCCVVMFIINWWAALLTNVIVLSLYIYVSYKKPDVNWGSSTQALTYHQALTHSLQLCGVADHIKTYRPQC
LVMTGAPNSRPAILHLVHAFTKNVGLMVCGHVRISSRRPNFKELNSDMVRYQRWLLNNKSKAFYTPVVAEDLRQGTQYLL
QAAGLGRLRPNTLVLGFKNDWCIGDIKDVETYINIIHDAFDFQYGVVILRLREGLDISHIQGQDDSSGMKDVVVSVDMSK
DSDGDSSKPSSKATSVQNSPAVQKDEDDDGKAHTQPLLKKDKKSPTLPLNVADQRLLDASQQFQQKQGKGTVDVWWLFDD
GGLTLLIPYLIANKKKWKDCKIRVFIGGKINRIDHDRRAMATLLSKFRIDFSDITVLGDINTKPKSEGLTEFAQMIEPYK
LREDDMEQEAAEKLKSDEPWRITDNELELYKAKSNRQIRLNELLKEHSSTANLIVMSMPLARKGAVSSALYMAWLDTLSK
DLPPILLVRGNHQSVLTFYS
>hNCC NP_000330.3 Homo sapiens
PVRFGWVKGVMIRCMLNIWGVILYLRLPWITAQAGIVLTWIIILLSVTVTSITGLSISAISTNGKVKSGGTYFLISRSLG
PELGGSIGLIFAFANAVGVAMHTVGFAETVRDLLQEYGAPIVDPINDIRIIAVVSVTVLLAISLAGMEWESKAQVLFFLV
IMVSFANYLVGTLIPPSEDKASKGFFSYRADIFVQNLVPDWRGPDGTFFGMFSIFFPSATGILAGANISGDLKDPAIAIP
KGTLMAIFWTTISYLAISATIGSCVVRDASGVLNDTVTPGWGACEGLACSYGWNFTECTQQHSCHYGLINYYQTMSMVSG
FAPLITAGIFGATLSSALACLVSAAKVFQCLCEDQLYPLIGFFGKGYGKNKEPVRGYLLAYAIAVAFIIIAELNTIAPII
SNFFLCSYALINFSCFHASITNSPGWRPSFQYYNKWAALFGAIISVVIMFLLTWWAALIAIGVVLFLLLYVIYKKPEVNW
GSSVQAGSYNLALSYSVGLNEVEDHIKNYRPQCLVLTGPPNFRPALVDFVGTFTRNLSLMICGHV
>DsKCC XP_016029323.1 Drosophila simulans
MGTLIGVFLPCIQNIFGVILFIRLTWVVGTAGAVCGFLIVLTCCCVTMLTAISMSAIATNGVVPAGGSYFMISRSLGPEF
GGAVGMLFYTGTTLAAAMYIVGAVEIVLTYMAPWASIFGDFTKDADAMYNNFRVYGTLLLIFMGLIVFLGVKFVNKFATV
ALACVILSIIAVYVGIFDNIHGNEKLYMCVLGKRLLKDIPLENCTKEDSFLRDIYCPDGKCEEYYLANNVTKVKGIKGLA
SGVFYDNIFPSFLEKGQFISYGKSAIDIENTSGESYNQIMADITTSFTLLIGIFFPSVTGIMAGSNRSGDLADAQKSIPI
GTICAILTTSTVYLSSVMFFAGTVDNLLLRDKFGQSIGGKLVVANIAWPNQWVILIGSFLSTLGAGLQSLTGAPRLLQAI
ARDEIIPFLAPFAKSSKRGEPTRALLLTIVICQCGILLGNVDLLAPLLSMFFLMCYGFVNLACAVQTLLRTPNWRPRFKF
YHWSLSLIGLTLCISVMIMTSWYFALIAMGMAIIIYKYIEYRGAEKEWGDGIRGMALTAARYSLL
>KAK7120047.1 Phoxinus phoxinus
MSASPPPLAGPGDSLSAPEPDALKPAGPTPSQSRFQVDLVSETAGDGHTHSASDSSPPAYDAEPPSDARRDSAAGGEEAK
GRFRVVNFAASSPDAAPADIAQNGDTVMSEGSLHSSTGGQQHHHYDTHTNTYYLRTFGHNTIDAVPNIDFYRQTAAPLGE
KLIRPTLSELHDELDKEPFEDGFANGEELTPAEEAAAKEASESKGVVKFGWIKGVLVRCMLNIWGVMLFIRMTWIVGQAG
IAYSCIIVIMATVVTTITGCSTSAIATNGFVRGGGAYYLISRSLGPEFGGSIGLIFAFANAVAVAMYVVGFAETVVELLI
SSGALMFDPINDVRIIGTITVILLLGISVAGMEWEAKAQIFLLVILITAIFNYFIGSFIRVESKEKYGFFGYDTGIFAEN
FGPDFRGQTFFSVFSIFFPAATGILAGANISGDLADPQLAIPKGTLLAILITGLVYIGVAISAGACIVRDATGIESNITL
SGIVNCTDAACKFGYDFNSCRPMVEGEKSACKFGLHHDFQVMSVVSGFSPLITAGIFSATLSSALASLVSAPKVFQALCK
DNIYPGIGVFGKGYGKNNEPLRGYLLTFGIALAFILIAELNVIAPIISNFFLASYALINFSVFHASLANSPGWRPSFKYY
NMWASLAGAILCCVVMFIINWWAALLTNVIVLSLYIYVSYKKPDVNWGSSTQALTYHQALTHSLQLCGVAEHIKTYRPQC
LVMTGAPNSRPAVLHLVHAFTKNVGLMVCGHVRISSRRPNFKELNSDMVRYQRWLLNNNSKAFYTPVVAEDLRQGTQYLL
QAAGLGRLRPNTLVLGFKNDWRIGDIKDVETYINLIHDAFDFQYGVVILRLREGLDISHIQGQDDSSGLKDVVVSVDMSK
DSDGDSSKPSSKATSVQNSPAVQKDKRSPTLPLNVADQRLLDASQQFQQKQGKGTVDVWWLFDDGGLTLLIPYLIANKKK
WKDCKIRVFIGGKINRIDHDRRAMATLLSKFRIDFSDITVLGDVNTKPKSEGLTEFAEMIEPYKLREDDMEQDAAEKMKS
EEPWRITDNELELYKAKSNRQIRLNELLKEHSSTANLIVMSMPLARKGAVSSALYMAWLDTLSKDLPPILLVRGNHQSVL
TFYS
>XP_016332275.1 Sinocyclocheilus anshuiensis
MSASPPPPAAASDHLSAPEPDALKPAGPTPSQSRFQVDLVSECAAETALASDSSPPEYSAEPLRYPVSGGEEAKGRFRVV
NFAASSPDSAPADCAQNGDTVMSEGSLHSSSGGQQHHHYDTHTNTYYLRTFGHNTIDAVPNIDFYRQTAAPLGEKLIRPT
LSELHDELDKEPFEDGFANGEELTPAEEAAAKEASESKGVVKFGWIKGVLVRCMLNIWGVMLFIRMTWIVGQAGIAYSCI
IVVMATVVTTITGCSTSAIATNGFVRGGGAYYLISRSLGPEFGGSIGLIFAFANAVAVAMYVVGFAETVVELLINSGALM
LDEINDVRIIGTITVILLLGVSVAGMEWEAKAQIFLLFILVTAIFNYFIGTFIPVESKEKFGFFSYDAGILADNFGPDFR
GETFFSVFSIFFPAATGILAGANISGDLADPQMAIPKGTLLAILITGLVYVGVAISAGACIVRDATGMESNVTLSGLNCT
DAACKFGYDFTSCRPVEGGKSACKFGLYNDFQVMSVVSGFGPLITAGIFSATLSSALASLVSAPKVFQALCKDNIYPGIA
VFGKGYGKNNEPLRGYFLTFGIALAFILIAELNIIAPIISNFFLASYALINFSVFHASLANSPGWRPSFKYYNMWVSLAG
AILCCVVMFIINWWAALLTNVIVLSLYIYVSYKKPDVNWGSSTQALTYHQALTHSLQLCGVADHIKTYRPQCLVMTGAPN
SRPALLHLVHAFTKNVGLMLCGHVRASSRRPNFKELNSDMLRYQRWLLNNSSKAFYTPVVAEDLRQGTQYLLQAAGLGRL
RPNTLVLGFKNDWRIGDIKDVETYINLMHDAFDFQYGVVILRLREGLDISHIQGQDDSSAMRDVVVSVDMSKDSDGDSSK
PSSKATSVQNSPAVQKDEDEDGKAHTRPLLKKEKRSPTLPLNVADQRLLDASQQFQQKQGKGTIDVWWLFDDGGLTLLIP
YLIANKKKWKDCKIRVFIGGKINRIDHDRRAMATLLSKFRIDFSDITVLGDINTKPKSEGLTEFAQMIEPYKLREDDMEQ
EAAEKLKSEEPWRITDNELELYRAKSNRQIRLNELLKEHSSTANLIVMSMPLARKGAVSSVLYMAWLDTISKDLPPILLV
RGNHQSVLTFYS
>XP_043106370.1 Puntigrus tetrazona
MSASPPPPAAAASDHLSAPEPDALKPAGPTPSQSRFQVDLVSECAASDSSPPEYSAEPPRDPASGGEEAKGRFRVVNFLA
SSPDAAPADSAQNGDTVMSEGSLQSSTGGQQHHHYDTHTNTYYLRTFGHNTIDAVPNIDFYRQTAAPLGEKLIRPTLSEL
HDELDKEPFEDGFANGEELTPAEEAAAKEASESKGVVKFGWIKGVLVRCMLNIWGVMLFIRMTWIVGQAGIAYSCIIVVM
ATVVTTITGCSTSAIATNGFVRGGGAYYLISRSLGPEFGGSIGLIFAFANAVAVAMYVVGFAETVVELLVSSGALMLDQI
NDVRIIGTITVILLLGISVAGMEWEAKAQIFLLFILVTAIFNYFIGTFIPVETKEKFGFFSYDVGILADNFGPDFRGQTF
FSVFSIFFPAATGILAGANISGDLADPQMAIPKGTLLAILITGVVYMGVAISAGACIVRDATGMDNNVTLSGLNCTDAAC
KFGYDFSSCRSGFEGGKSTCKFGLHNDFQVMSVVSGVGPLITAGIFSATLSSALASLVSAPKVFQALCKDNIYPGIAIFG
KGYGKNNEPLRGYFLTFGIALAFILIAELNVIAPIISNFFLASYALINFSVFHASLANSPGWRPSFKYYNMWVSLAGAIL
CCVVMFIINWWAALLTNVIVLSLYIYVSYKKPDVNWGSSTQALTYHQALTHSLQLCGVADHIKTYRPQCLVMTGAPNSRP
ALLHLVHAFTKNVGLMLCGHVRVSSRRPNFKELNSDMLRYQRWLLNKNSKAFYTPVVAEDLRQGTQYLLQAAGLGRLRPN
TLVLGFKNDWRIGDIKEVETYINLIHDAFDFQYGVVILRLREGLDISHIQGQDDSSAMKDVVVSVDMSKDSDGDSSKPSS
KATSVQNSPAVQKDEDEDGRAYTRPLLKKEKRSPTVPLNMAEQRLLDASQQFQQKQGKGTIDVWWLFDDGGLTLLIPYLI
ANKKKWKDCKIRVFIGGKINRIDHDRRAMATLLSKFRIDFSDITVLGDINTKPKSEGLTEFAQMIEPYKLREDDMEQEAA
EKLKSEEPWRITDNELELYKAKSNRQVRLNELLKEHSSTANLIVMSMPLARKGAVSSALYMAWLETLSKDLPPILLVRGN
HQSVLTFYS
>XP_051539485.1 Myxocyprinus asiaticus
MSASPPPDAATTQSELLPPAEPDALKAAGPTPSQSRFQVDLVSESAGDGHDPGAGGEEAKGRFRVVNFAASSPDAAPAES
AQNGDTVMSEGSLHSSTGGQQHHHYDTHTNTYYLRTFGHNTIDAVPNIDFYRQTAAPLGEKLIRPTLSELHDELDKEPFE
DGFANGEELTPAEEAAAKEASESKGVVKFGWIKGVLVRCMLNIWGVMLFIRMTWIVGQAGIAYSCLIVLMATVVTSITGC
STSAIATNGFVRGGGAYYLISRSLGPEFGGSIGLIFAFANAVAVAMYVVGFAETVVELLNAAGALMLDPINDVRIIGTIT
VILLLGISVAGMEWEAKAQIFLLVILVTAIFNYFIGSFIPVESKEKYGFFGYDAGILADNFGPDFRGETFFSVFSIFFPA
ATGILAGANISGDLADPQMAIPRGTLLAILITGVVYMGVAISSGACIVRDATGIESNSTLSSTINCTDAACKYGYDFTSC
RPVVEGAPSPCKYGLHHDFQVMSVVSGFSPLITAGIFSATLSSALASLVSAPKVFQALCKDNIYPGIAGFAKGYGKNNEP
LRGYFLTFGIALAFILIAELNVIAPIISNFFLASYALINFSVFHASLANSPGWRPSFKYYNMWVSLAGAILCCVVMFVIN

WWAALLTNVIVLSLYIYVSYKKPDVNWGSSTQALTYHQALTHSLQLCGVADHIKTYRPQCLVMTGAPNSRPVILHLVHAF
TKNVGLMVCGHVRISSRRPNFKELNNDTLRYQRWLLNNNSKAFYSPVVAEDLRQGTQFLLQAAGLGRLRPNTLVLGFKNN
WRTGDIKDVETYISVIHDAFDFQYGVVILRLREGLDISHIQGQDDSSGMKDVVVSVDMSKDSDGDSSKPSSKATSVQNSP
AVQKDEDEDGKAYTQPLLKKEKTSPTVPLNVADQQLLDASKQFQRKQGKGTVDVWWLFDDGGLTLLIPYLITNKKKWKDC
KIRVFIGGKINRIDHDRRAMATLLSKFRIDFSDITVLGDINTKPRSEGVTEFAEMIEPYKLREDDMEQEAAEKLKSEEPW
RITDNELELYKAKSNRQIRLNELLMEHSSTANLIVMSMPLARKGAVSSALYMAWLDTLSKDLPPILLVRGNHQSVLTFYS
>XP_042610103.1 Cyprinus carpio
MSASPPPPAAASDHLSAPDALKSRPTPSQSRFQVDLVSEAAGDGGTAVASDSPPEYAAVPPFDGLRDSEEAKGRFRVVNF
AASSPDAAPAEIAQNGDTVMSESSQQHHHYDTHTNTYYLRTFGHNTIDAVPNIDFYRQTAAPLGEKLIRPTLSELHDELD
KEPFEDGFANGEELTPAEEAAAKEASESKGVVKFGWIKGVLVRCMLNIWGVMLFIRMTWIVGQAGIAYSCIIVIMATVVT
TITGCSTSAIATNGFVRGGGAYYLISRSLGPEFGGSIGLIFAFANAVAVAMYVVGFAETVVELLNSSGALMFDVTNDIRI
VGTITVILLLGISVAGMEWEAKAQIFLLVILISAIFNYFIGTFIRVESKEKFGFFSYDVGILAENFGPDFRGETFFSVFS
IFFPAATGILAGANISGDLADPQMAIPKGTLLAILITGLVYMGVAISAGASIVRDATGIESNMTINGSNCTDAACKFGYD
FTSCRPAARGGKTACEFGLHNDFQVMSVVSGFGPLITAGVFSATLSSALASLVSAPKVFQALCKDNIYPGIGVFGKGYGK
NNEPLRGYLLTFGIALAFIVIAELNVIAPIISNFFLASYALINFSVFHASLANSPGWRPSFKYYNMWVSLAGAILCCVVM
FIINWWAALLTNVIVLSLYIYVSYKKPDVNWGSSTQALTYHQALTHSLQLCGVADHIKTYRPQCLVMTGAPNSRPALLHL
VHAFTKNVGLMVCGHVRISSRRPNFKELNSDMVRYQRWLLHNNSKAFYTPVVGEDLRQGTQYLLQAAGLGRLRPNTLVLG
FKNDWRIGDIKDVETYINLMHDAFDFQYGVVILRLREGLDISHIQGQDDSSAVKDVVVSIDMSKDSDADSSKPSSKATSV
QNSPAVQKDEDEDGKAHTQPLLKKDERSPALPLNVADQRLLDASQQFQQKQGKGTIDVWWLFDDGGLTLLIPYLIANKKK
WKDCKIRVFIGGKINRIDHDRRAMATLLSKFRIDFSDITVLGDINTKPKSEGLTEFAQMIEPYKLREDDMEQEAAEKLKS
EEPWRITDNELELYKAKSNRQIRLNELLKEHSSTANLIVMSMPLARKGAVSSALYMAWLDTLSKDLPPILLVRGNHQSVL
TFYS
>XP_016114419.1 Sinocyclocheilus grahami
MSASPPPPAAASDHLSIKPAGPTPSQSRFQVDLVSECAAASDSSPPEYPAEPLRDSASGGEEAKGRFRVVNFAASSPDAA
PADCAQNGDTVMSEGSLHSSSGGQQHHHYDTHTNTYYLRTFGHNTIDAVPNIDFYRQTAAPLGEKLIRPTLSELHDELDK
EPFEDGFANGEELTPAEEAAAKEASESKGVVKFGWIKGVLVRCMLNIWGVMLFIRMTWIVGQAGIAYSCIIVVMATVVTT
ITGCSTSAIATNGFVRGGGAYYLISRSLGPEFGGSIGLIFAFANAVAVAMYVVGFAETVVELLINSGALMLDEINDVRII
GTITVILLLGVSVAGMEWEAKAQIFLLFILVTAIFNYFIGTFIPVESKEKFGFFSYDAGILADNFGPDFRGETFFSVFSI
FFPAATGILAGANISGDLADPQMAIPKGTLLAILITGLVYVGVAISAGACIVRDATGMESNVTLSGLNCTDAACKFGYDF
SSCRPVEGGKSACKFGLYNDFQVMSVVSGFGPLITAGIFSATLSSALASLVSAPKVFQALCKDNIYPGIAVFGKGYGKNN
EPLRGYFLTFGIALAFILIAELNIIAPIISNFFLASYALINFSVFHASLANSPGWRPSFKYYNMWVSLAGAILCCVVMFI
INWWAALLTNVIVLSLYIYVSYKKPDVNWGSSTQALTYHQALTHSLQLCGVADHIKTYR
>KAK2870718.1 Cirrhinus molitorella
MSASPPPPAAATAACDYLSASEPDALKPAGPTPSQSRFQVDLVSESAGTPLALDLSPPEYSLEPPEHRDSTAGGEEAKGR
FRVVNFAASSPDAAPPESAQNGDTVMSEGSLHSSTGGQQHHHYDTHTNTYYLRTFGHNTIDAVPNIDFYRQTAAPLGEKL
IRPTLSELHDELDKEPFEDGFANGEELTPAEEAAAKEASESKGVVKFGWIKGVLVRCMLNIWGVMLFIRMTWIVGQAGIA
YSCIIVIMATVVTTITGCSTSAIATNGFVRGGGAYYLISRSLGPEFGGSIGLIFAFANAVAVAMYVVGFAETVVELLISS
NVLMLDEINDVRIIGTITVILLLGISVAGMEWEAKAQIFLLVILITAIFNYFIGAFIPVESKKKFGFFGYDAGILADNFG
PDFRGQTFFSVFSIFFPAATGILAGANISGDLADPQSAIPKGTLLAILITGLVYMGVAISAGACIVRDATGQDMGINETF
SALNCTDAACRLGYDFSSCRPILEGGKSSCKFGLHNDFQIMSVVSGFGPLITAGIFSATLSSALASLVSAPKVFQALCKD
NIYPGIGIFGKGYGKNNEPLRGYFLTFGIALAFILIAELNVIAPIISNFFLASYALINFSVFHASLANSPGWRPSFKYYN
MWVSLAGAILCCLVMFVINWWAALLTNVIVLSLYIYVSYKKPDVNWGSSTQALTYHQALTHSLQLCGVADHVKTYRPQCL
VMTGAPNSRPALLHLVHAFTKNVGLMVCGHVRISSRRPNFKELNSDMLRYQRWLLNNNSKAFYTPVVAEDLRQGTQYLLQ
AAGLGRLRPNTLVVGFKNDWRIGDIKEVETYINLIHDAFDFQYGVVIIRLREGLDISHIQGQDDSSAMKDVVVSVDLSKD
SDGDSSKPSSKATSVQNSPAVQKDEDEDGKAHTRPLLRKDKKSPTIPLNVADQRLLDASQQFQQKQGKGTIDVWWLFDDG
GLTLLIPYLIANKKKWKDCKIRVFIGGKINRIDHDRRAMATLLSKFRIDFSDITVLGDVNTKPKSEGLTEFAQMIEPYKL
REDDMEQEAAEKLKSEEPWRITDNELELYKAKSNRQIRLNELLKEHSSTANLIVMSMPLARKGAVSSALYMAWLDTLSKD
LPPILLVRGNHQSVLTFYS
>XP_059395758.1 Carassius carassius
MSASPPPPAASDHLSAPEPDALKPAGPTPSQSRFQVDLVSECAAHTLASDSSPPEYSAEPPRDPASGGEEAKGRFRVVNF
AASSPDAAPGDSAQNGDTVMSEGSLHSSTGGQQHHHYDTHTNTYYLRTFGHNTIDAVPNIDFYRQTAAPLGEKLIRPTLS
ELHDELDKEPFEDGFANGEDLTPAEEAAAKEASESKGVVKFGWIKGVLVRCMLNIWGVMLFIRMTWIVGQAGIAYSCIIV
IMATVVTTITGCSTSAIATNGFVRGGGAYYLISRSLGPEFGGSIGLIFAFANAVAVAMYVVGFAETVVELLINSNALMLD
EINDVRIIGTITVILLLGISVAGMEWEAKAQIFLLFILVTAIFNYFIGTFIPVESKEKFGFFGYDAGILADNFGPDFRGQ
TFFSVFSIFFPAATGILAGANISGDLADPQMAIPKGTLLAILITGLVYMGVAISAGACIVRDATGMESNVTLSGLNCTDA
VCKFGYDFTSCRPVAGGKNACKFGLHNDFQVMSVVSGFGPLITAGIFSATLSSALASLVSAPKVFQALCKDNIYPGIGVF
GKGYGKNNEPLRGYLLTFGIALAFIVIAELNVIAPIISNFFLASYALINFSVFHASLANSPGWRPSFKYYNMWVSLAGAI
LCCVVMFIINWWAALLTNVIVLSLYIYVSYKKPDVNWGSSTQALTYHQALTHSLQLCGVADHIKTYRPQCLVMTGAPNSR
PALLHLVHAFTKNVGLMLCGHVRISSRRPNFKELSSDMLRYQRWLLNNSSKAFYTPVVAEDLRQGTQYLLQAAGLGRLRP
NTLVLGFKNDWRIGDIKDVETYINLMHDAFDFQYGVVILRLREGLDISHIQGQDDSSAMKDVVVSVDMSKDSDGDSSKPS
SKATSVQNSPAVQKDEDEDGKAYTRPLLKKEKMSPTVPLNVADQRLLDASQHFQQKQGKGTIDVWWLFDDGGLTLLIPYL
IANKKKWKDCKIRVFIGGKINRIDHDRRAMATLLSKFRIDFSDITVLGDINTKPKSEGLTEFAQMIEPYKLREDDMEQEA
AERLKSEEPWRITDNELELYKAKSNRQIRLNELLKEHSSTANLIVMSMPLARKGAVSSALYMAWLDTLSKDLPPILLVRG
NHQSVLTFYS
>XP_051973837.1 Xyrauchen texanus
MSASPPSFAASRSELLPPVEPVALKPAGPSPSQSRFQVDIVSECAGDGHAPPASDPSPLDLPADPPHDPGEEAKGRFRVV
NFAASSPDAAPAESAQNGDTVMSEGSLHSSTGGQQHHHYDTHTNTYYLRTFGHNTIDAVPNIDFYRQTAAPLGEKLIRPT
LSELHDELDKEPFEDGFANGEELTPAEEAAVKEASESKGVVKFGWIKGVLVRCMLNIWGVMLFIRMTWIVGQAGIAYSCL
IVIMATVVTSITGCSTSAIATNGFVRGGGAYYLISRSLGPEFGGSIGLIFAFANAVAVAMYVVGFAETVVELLSAAGALM
LDPINDVRIIGTITVILLLGISVAGMEWEAKAQIFLLVILVTAIFNYFIGSFIPVESKEKYGFFGYDAGILADNFGPDFR
GETFFSVFSIFFPAATGILAGANISGDLADPQMAIPRGTLLAILITGVVYMGVAVSSGACIVRDATGIESNRTLSASINC
TDAACNLGYDFTSCRPAVKGVSSPCKYGLHNDFQVMSVVSGFGPLISAGIFSATLSSALASLVSAPKVFQALCKDNIYPG
IAGFAKGYGKNNEPLRGYLLTFGIALAFILIAELNVIAPIISNFFLASYALINFSVFHASLANSPGWRPSFKYYNMWVSL
AGAILCCVVMFVINWWAALLTNVIVLSLYIYVSYKKPDVNWGSSTQALTYHQALTHSLQLCGVADHIKTYRPQCLVMTGA
PNSRPAILHLVHAFTKNVGLMVCGHVRISSRRPNFKELNNDTLRYQRWLLHNNSKAFYTPVVAEDLRQGTQYLLQAAGLG
RLRPNTLVLGFKNDWRTGDIKDVETYINIIHDSFDFQYGVVILRLREGLDISHIQGQDDSSGMKDVVVSVDMSKDSDRDS
SKPSSKATSVQNSPAVQKEKTSPTVPLNVADQQLLDASQQFQRKQGKGTVDVWWLFDDGGLTLLIPYLINNKKKWKDCKI
RVFIGGKINRIDHDRRAMATLLSKFRIDFSDITVLGDINTKPKSEDVTKFADMTEPYKLREDDMEQEAAEKLKSEEPWRI
TDNELELYKAKSNRQIRLNELLKEHSSTANLIVMSMPLARKGAVSSALYMAWLDTLSKDLPPILLVRGNHQSVLTFYS

>NP_005063.1 hKCC1 Homo sapiens
PSMGTLMGVYLPCLQNIFGVILFLRLTWMVGTAGVLQALLIVLICCCCTLLTAISMSAIATNGVVPAGGSYFMISRSLGP
EFGGAVGLCFYLGTTFAAAMYILGAIEILLTYIAPPAAIFYPSGAHDTSNATLNNMRVYGTIFLTFMTLVVFVGVKYVNK
FASLFLACVIISILSIYAGGIKSIFDPPVFPVCMLGNRTLSRDQFDICAKTAVVDNETVATQLWSFFCHSPNLTTDSCDP
YFMLNNVTEIPGIPGAAAGVLQENLWSAYLEKGDIVEKHGLPSADAPSLKESLPLYVVADIATSFTVLVGIFFPSVTGIM
AGSNRSGDLRDAQKSIPVGTILAIITTSLVYFSSVVLFGACIEGVVLRDKYGDGVSRNLVVGTLAWPSPWVIVIGSFFST
CGAGLQSLTGAPRLLQAIAKDNIIPFLRVFGHGKVNGEPTWALLLTALIAELGILIASLDMVAPILSMFFLMCYLFVNLA
CAVQTLLRTPNWRPRFKYYHWALSFLGMSLCLALMFVSSWYYALVAMLIAGMIYKYI

```
>NP_001128243.1 hKCC2 Homo sapiens
PRMGTFMGVYLPCLQNIFGVILFLRLTWVVGIAGIMESFCMVFICCSCTMLTAISMSAIATNGVVPAGGSYYMISRSLGP
EFGGAVGLCFYLGTTFAGAMYILGTIEILLAYLFPAMAIFKAEDASGEAAAMLNNMRVYGTCVLTCMATVVFVGVKYVNK
FALVFLGCVILSILAIYAGVIKSAFDPPNFPICLLGNRTLSRHGFDVCAKLAWEGNETVTTRLWGLFCSSRFLNATCDEY
FTRNNVTEIQGIPGAASGLIKENLWSSYLTKGVIVERSGMTSVGLADGTPIDMDHPYVFSDMTSYFTLLVGIYFPSVTGI
MAGSNRSGDLRDAQKSIPTGTILAIATTSAVYISSVVLFGACIEGVVLRDKFGEAVNGNLVVGTLAWPSPWVIVIGSFFS
TCGAGLQSLTGAPRLLQAISRDGIVPFLQVFGHGKANGEPTWALLLTACICEIGILIASLDEVAPILSMFFLMCYMFVNL
ACAVQTLLRTPNWRPRFRYYHWTLSFLGMSLCLALMFICSWYYALVAMLIAGLIYKYIEYRGAEK
>NP_598408.1 hKCC3 Homo sapiens
PQMGTFMGVYLPCLQNIFGVILFLRLTWVVGTAGVLQAFAIVLICCCCTMLTAISMSAIATNGVVPAGGSYFMISRALGP
EFGGAVGLCFYLGTTFAAAMYILGAIEIFLVYIVPRAAIFHSDDALKESAAMLNNMRVYGTAFLVLMVLVVFIGVRYVNK
FASLFLACVIVSILAIYAGAIKSSFAPPHFPVCMLGNRTLSSRHIDVCSKTKEINNMTVPSKLWGFFCNSSQFFNATCDE
YFVHNNVTSIQGIPGLASGIITENLWSNYLPKGEIIEKPSAKSSDVLGSLNHEYVLVDITTSFTLLVGIFFPSVTGIMAG
SNRSGDLKDAQKSIPIGTILAILTTSFVYLSNVVLFGACIEGVVLRDKFGDAVKGNLVVGTLSWPSPWVIVIGSFFSTCG
AGLQSLTGAPRLLQAIAKDNIIPFLRVFGHSKANGEPTWALLLTAAIAELGILIASLDLVAPILSMFFLMCYLFVNLACA
LQTLLRTPNWRPRFRYYHWALSFMGMSICLALMFISSWYYAIVAMVIAGMIYKYIEYQGAEKEWG
>NP_006589.2 hKCC4 Homo sapiens
PRMGTFIGVYLPCLQNILGVILFLRLTWIVGVAGVLESFLIVAMCCTCTMLTAISMSAIATNGVVPAGGSYYMISRSLGP
EFGGAVGLCFYLGTTFAGAMYILGTIEIFLTYISPGAAIFQAEAAGGEAAAMLHNMRVYGTCTLVLMALVVFVGVKYVNK
LALVFLACVVLSILAIYAGVIKSAFDPPDIPVCLLGNRTLSRRSFDACVKAYGIHNNSATSALWGLFCNGSQPSAACDEY
FIQNNVTEIQGIPGAASGVFLENLWSTYAHAGAFVEKKGVPSVPVAEESRASALPYVLTDIAASFTLLVGIYFPSVTGIM
AGSNRSGDLKDAQKSIPTGTILAIVTTSFIYLSCIVLFGACIEGVVLRDKFGEALQGNLVIGMLAWPSPWVIVIGSFFST
CGAGLQSLTGAPRLLQAIARDGIVPFLQVFGHGKANGEPTWALLLTVLICETGILIASLDSVAPILSMFFLMCYLFVNLA
CAVQTLLRTPNWRPRFKFYHWTLSFLGMSLCLALMFICSWYYALSAMLIAGCIYKYIEYRGAEKE

>KAA3622248.1 FsCCC Flavobacterium sp.
MDNSEHIKKFGTFGGVFTPTLLTILGVIMYLRMGWVVGNAGLLGAWLIIIISFLITLCTALSMSAITTNIRIGAGGAYAI
ISQSLGLEVGGSLGIPRYISQGLAVTMYIFGFREGWLGIFPDHNPFLVDISAFIGLFTIAYISANLAIKTQYIIMVVIVA
SLISIVMAAYDGSMAIATDEALKWGTFKGSPENDFSGSNFWLVFAVFFPAATGIMAGANMSGELKEPRKSIPVGTLWAIG
VSFVIYMLLAFWLARTATEQELISNYNIIIEKAYVGPLIIAGVLGATFSSALASIVGSSRILYAMGEHNVLPYSRFLAGQ
SKNGQPRNAMMVTGILIFATMLLRNLNAVAPLVTLFFLVTYAMINIVVIIEQNLGLISYRPIFKVKRWIPWLGLISSILA
MFIINPTISLITIAIVLAVYWFLSRQNLETPFEDVRAGLFVSVAEWAAKHTWGM
>WP_244592994.1 Pukyongia salina
MDKCLIIVTTTMDDNKHIKKFGTFGGVFTPTLLTILGVIMYLRMGWVVGNAGLLGAWLIIIISFLITLCTALSMSAITTN
IRIGAGGAYAIISQSLGLEVGGSLGIPRYISQGLAVTMYIFGFREGWLGIFPDHNPFLVDISAFIGLFTIAYISANLAIK
TQYIIMVVIVASLVSVVMAAYDGSMSIATDEALKWGTFKGSPENNFRGSNFWLVFAVFFPAATGIMAGANMSGELKDPRK
SIPVGTLWAIGVSFVIYMLLAFWLARTATEQELISNYNIIIEKAYVGPLIIAGVLGATFSSALASIVGSSRILFAMGEHN
VLPYSKFLAGQSKNGQPRNAMLVTGILIFATMLLRNLNAVAPLVTLFFLVTYAMINIVVIIEQNLGLISYRPIFKVKRWI
PWLGLVSSVLAMFIINPTISLITIAIVLAVYWYLSRQNLETPFEDVRSGLFVSFAEWAAKHTWGMKKMQQRAWKPNLMVP
VRDIIGVRGNFEFLRNIASPKGSIKLLGIEANSEESEMTKHLDELAASFRDKGVFSSWTVINTTEFAKGVNYGNQALRGA
FFRPNVVFLNLQQHDDYETELRPVIKECIRLEIGVLLFQSHPTALLGQRNTINVWVSNRKNNWSLGWDIGNLDLSTLIAY
KLMKNWKARIRLITVIDDVEEVENATKFLDSLINLARLPETLMEVCVGDFKDVVTAAPHADLNIFGMQEDLRFEFVQEMT
SKTNSSCLFVKDSGHESILA
>MBT8261550.1 Bacteroidia bacterium
METNQPQIKKFGTFGGVFTPTLLTILGVIMYLRLGWVVGNAGLIGAWLIIIISFLITLCTALSMSAITTNIRIGAGGAYA
IISQSLGLEVGGSLGIPRYISQGLAVTMYIFGFREGWLGIFPDHNAFLVDVSAFIGLFTIAYISANLAIKTQYLIMVVII
ASLVSIVLAAYDGSMVIASDEALKWGTFKGAPENDFKGSDFWLVFAVFFPAATGIMAGANMSGELKSPRKSIPIGTLWAI
GVSFVIYMLLAFWLARTATEQELISNYNIIIDKAYVGPLIIAGVLGATFSSALASIVGSSRILFAMGQHNVLPYSGFLAG
QSKNGQPRNAMLVTGILIFGTMLLRNLNAVAPLVTLFFLVTYAMINIVVIIEQNLGLISYRPIFKVKRWIPWLGLISSVL
AMFIINPTISLITIAIVLAVYWYLSRQNLETPFEDVRSGLFVSFAEWAAKHTWGMKNMQQRAWKPNLMVPVRDLTGARGN
FEFLRNIAAPKGSIKLLGIEPDSENSSMTSELDTLAESFRQRGVFSSWTVINTTEFAKGVNYGNQALRGAFFRPNIVFLN
LQQHDDYETELRPVIRECIRLEIGVLLYQSHAKALLGQRNTINVWVSNRKNNWSLGWDIGNLDLSTLIAYKLKRNWKARI
RLITVIDDDSELENATIFLNSLTNLARLPETLTQVFVGDFKDVVVKAPQADLNIFGMHADLQFEFVQEMTEKTNSSCLFV
KDSGHESILA
>NNK83001.1 Flavobacteriaceae bacterium
MDNQPKTIKKFGTFGGVFTPTLLTILGVIMYLRMGWVVGNAGLLGAWLIIIVSFLITLCTALSMSAITTNIRIGAGGAYA
IISQALGLEVGGSLGIPRYISQGLAVAMYIFGFREGWLGIFPTHDPFLVDIAVFLFLFTIAYISANLAIKTQYVIMVVII
LSLVSIVVAAYNGSMEIPTSEALSWGSFQGSSENDFSGSNFWIVFAVFFPAATGIMAGANMSGELKEPKRSIPVGTLWAI
GVSFIIYMALAYWLARTATEEELLTNYNIIIDKAFNGPLIIAGILGATFSSALASIVGSSRILFAMGEHRVLPYSNFLSG
QSANGQPRNAMIVTGVLIFATMLLRNINAIAPLVTLFFLVTYAMINIVVIIEQRLGLISYRPLFKVHKWIPWLGLISSVL
AMFIINPTISWITIFIVFIVYWYLSKQNLETPFEDVRSGLFVSFAEWAAKHTWGMKSMQQRAWKPNLMVPVRDIVGAKGN
FQFLRNIAKPKGSIKLLGIEPFCENSKLVKELDKLSETFVHKGVFSSWTVINTEEFAKGVNYGNQALKGAFFRPNIIFLN
LQDHDDYENEIRPVIKESI
>WP_343485566.1 Allomuricauda sp.
MQAQESILKKFGTFGGVFTPTLLTILGVIMYLRMGWVVGNAGLLGAWLIIIISFLITLCTALSMSAITTNIRIGAGGAYA
IISQALGLEVGGSLGIPRYISQGLAVTMYIFGFREGWLGIFPGHDPFLVDIITFAILITIAYISADLAIKTQFFIMVVII
LSLISIVLAAYHGSMYLPTEDALRWGTFKGSPENNFSGSSFWIVFAVFFPAATGIMAGANMSGELKNPKKSIPQGTLWAI
GVSFVIYMLLAYWLARSASEEELISNYNIIIDKSYFGPLVIAGILGATFSSALASIVGSSRILFAMGEHKVLPYSKFLSG
QSANGQPRNAMLVTGIMIFATMLLRELNAVAPLVTLFFLITYAMINIVVIIEQRLGLISYRPIFKVNKWIPWIGLFSSVL
AMFIINPTISLVSIVIVFVVYWFLSRQNLETPFEDVRSGLFVSFAEWAAKHTWGMKSMQQRAWKPNLMVPVRDINGAKGN
FQFLRNIAAPKGSINLLGIASTENNAFVNELTSLSESFRLKGVFSSWTVIHTDDFAKGVNYGNQALRGAFFRPNIVFLNL
QQHDDYETEIRPVIKECIRLEIGVLLFSAHPTALLGQRNLINVWVSDRRGNWELGWDIGSLDLSTLIAYKLKKNWDARIR
IITVISNAEEEENANNFLKSLISLARLPETFIEVYVGNFREIVQAVPRADLNIFGMDENFKVEFVKEMIDKTNSSCLFVK
DSGHESILA
>WP_368049775.1 Lutibacter sp.
MDNQTKTIKKFGTFGGVFTPTLLTILGVIMYLRMGWVVGNAGLLGAWLIIIISFLITLCTALSMSAITTNIRIGAGGAYA
LVSQALGLEVGGSLGIPRYISQGLAVTMYIFGFREGWLGVFPEHNAFLVDIIVFVVLFTIAYISANLAIKTQFIILGIIA
LSIVSIIIAAYDGSMTIPTSEALSWGSFKGSVENGFSGSNFWIVFAVFFPAATGIMAGANMSGELKNPKQSIPTGTLWAI
GVSFVIYILLAFWISRSATEEELLNNYYIMVEKAYVGPVIIAGILGATFSSALASIIGSSRILFAMGEHKVLPYSKFLAG
QSTNGQPRNAMLVTGILIFLTLLLRNLNAVAPLVTLFFLITYAMINIVVIIEQNLGLISYRPVFKIHRWVPWFGLISSVF
TMFIINPTISLLSITIVLAVYWYLSRQNLETPFEDVRSGLFVSFAEWAAKHTWGMKKMQQRAWKANLMVPVRDVNGLKGT
FQFLRNIAKPKGSIKLLGIEPFSETSTLAEELEAISTSFRQKEVFSSSSVIHTGEFAKGINYGSQALQGAFFKPNIVFLN
MQDHDDYENELRPVMKESIRLQIGVLLYLSHPTALLGQRNTINVWISDRQNNWDLGWDIGNLDLSLLVAYKLKMNWGARI
RLITVITDSNEENNAKEFLSSLINLARLPETLTEVYIGDFNSIIMKAPPADLNIFGMHEDLEFGFVKEMSEKTKSTCLFV
KDSGHESILA
>MEO1010969.1 Bacteroidota bacterium
MESKDQQQKKFRTFGGVFTPTLLTILGVIMYLRMGWVVGNAGLLGAWLIILISFTITLTTALSMSAITTNIRIGAGGAYA
IISQALGLEVGGSLGIPRYISQGLAVTMYIFGFREGWLGIFPDHNAFLVDIIVFAVLLTIAYISADLAIKTQFFIMGVII
```

LSLLSIVLAAYYGSMHIATEEAVRWGSFKGSPENGFTGSDFWIVFAVFFPASTGIMAGANMSGELKDPRKSIPLGTLWAI
AVSFVIYVFLAYWLAKSATEEELVSNYNIIIEKAYVGPLIIAGILGATFSSALASIVGSSRILFAMGEHRVLPYARFLAG
QSANGQPRNAMMVTGIMIFVTMLLRDLNAVAPLVTLFFLITYAMINIVVIIEQNLGLISYRPIFKVHKWIPWLGLVSSVL
AMFIINPTVSLVSISIVFVVYWFLSRQNLETPFEDVRSGLFVSFAEWAAKHTWGMKSMQQRAWKPNLMVPVRDIKGAKGN
FQFLRNIARPKGSIKLLGIAPYTKENSFTDELSQLSEAFYAKGVFSSWTVIHAEDFARGVNYGNQALRGAFFRPNIVFLN
LQQHDDYETELRPVIKECIRLEIGVLLYQAHPTALLGQRNTINVWVSDRGGNWSLGWDIGNLDLSTLIAYKLKKNWDAKI
RLITVIKDEGEYENAKTFLDSLINLARLPDTLTEIHVGQFKEVIKKASPADLNIFGMHADLRFEFVTEMTQKTESSCLFV
KDSGHESILA
>WP_224477966.1 Winogradskyella vincentii
MANQPKALKKFGTFGGVFTPTLLTILGVIMYLRLGWVVGNAGLLGAWLIIIMSFLITLCTALSMSAITTNIRIGAGGAYA
IVSQALGLEVGGSLGIPRYVSQGLAVTMYIFGFREGWLGIFPDHNAFLVDVIVFGVLFTIAYISANLAIKTQFIIMGIII
LSLVSIVVAAYEGSMTLPVSDALSWGSFKGSMENGFSGSSFWMVFAVFFPAATGIMAGANMSGELKDPKKSIPNGTLWAI
AVSFVIYMLLAFWISRSATEQELVSDYYIMVEKAYFGPLIIAGILGATFSSALASIVGSSRILFAMGEHNVLPYSKLLAG
QSKTGQPRNAMLVTGILIFLTLLLRNLNAVAPLVTLFFLITYAMINIVVIIEQNLGLISYRPIFKIKRWVPWLGLISSIF
AMFIINPTVSLISIMIVLTVYWYLSRQNLETPFEDVRSGLFVSFAEWAAKHTWGMKKMQQRAWKANLMVPVRDVNGLKGT
FEFLRNIAKPKGSIKLLGIEPFTEHSSLALKLDDISASFRQKGVFSSSTVIHTNEFANGINYGSQALQGAFFRPNILFLN
LQDHDDYENELRPVMNESIRLEIGILLFSLHKTGLLGQRNVINVWVSDRRGNWELGWDIGNLDLSILIAYKLKMNWTAQI
RLITVLENKEDEAEATEFLDKLINLARLPNTLTEIHIGKFSDVVTVAPSADLNIFGMEPNLKFEFIKEMTTKTSSSCLFV
KDSGHESILA
>NNL01109.1 Eudoraea sp.
MKTKETVLKKFGTFGGVFTPTLLTILGVIMYLRLGWVVGNAGLLGAWIIILVSFLITLTTALSMSAITTNIRIGAGGAYA
IVSQALGLEVGGSLGIPRYISQGLAVTMYIFGFREGWLGIFPNHSPFLVDVIVFLALFGIAYYSANLAIKTQYIIIVIIV
LSFISIVFAAYEGSMVNSTSEVLRFGNFKGSPENDFSGSNFWIVFAVFFPAATGIMAGANMSGELKDPKKSIPVGTLWAI
GVSFIIYMLLAFWIARSATESELISNYYVIVEKAYVGPLILAGILGATFSSALASIVGSSRILYAMGEHKVLPYSKFLAG
TSANGQPRNAMIITGILIFATMLLRNINAVAPLVTLFFLITYAMINIVVIIEQNLGLISYRPIFKIPRWVPWAGLISSIF
AMFIINPSVSLLSLMIVFVVYWYLSRQNLETPFEDVRSGLFVSFAEWAAKHTWGMKKMQQRAWKPNLMVPVRDINGVKGI
FPFLRNIAKPKGSIKLLGIEPFSEKSTLANQLEALSESFRTKGVFSSWTVIHTDEFTKGINYGSQALQGAFFKPNIIFLN
LQEHDDYEQELRPIIKESIRLETGVLLYVAHPTALLGQRNTINVWISDRRDNWSLGWDIGNLDLSMLIAYKLKLNWDAKI
RLITVINDPKEEENAKKFLHSLINLARLPQTLTEVYVGSFKEIVEQAPIADLNIFGMEENLSFHFVKEMTYKTNSSCLFV
KDSGHESILA
>WP_290292714.1 Lutimonas halocynthiae
MINEPKTIKKFGTFGGVFTPTLLTILGVIMYLRLGWVVGNAGLLGAWLIIIISFLITLCTALSMSAITTNIRIGAGGAYA
IVSQALGLEVGGSLGIPRYISQGLAVTMYIFGFREGWLGIFPEHNAFLVDISVFVILYTIAYISANLAIKTQFIIMAIIV
LSLISIAVAAYDGSMTIPTSEALSWGSFKGSIENDFSGSNFWIVFAVFFPAATGIMAGANMSGELEFPKKSIPSGTLWAI
GVSFVIYMVLAFWIARSATENELLNDYYIMVDKAFFGPLVIAGILGATFSSALASIVGSSRILFAMGEHQVLPYSNFLKG
QSKNGQPRNAMIVTGILIFSTLLLRNINAVAPLVTLFFLITYAMINIVVIIEQNLGLISYRPVFKIHRWVPWFGLFSSVF
AMFIINPAVSLISIMIVLMVYWYLQRQNLETPFEDVRSGLFVSFAEWAAKHTWGMKNMQQRAWKANLMVPVRDISGLKGN
FEFLRNIAKPKGSIKLLGIEPFSASSSLASELEAISAAFRQKEVFSSSSVIHTQEFAKGINYGNQALQGAFFRPNIVFLN
LQDHDNYETELRPVMKECIRLEIGVLLYLSHPTALLGQRNTINVWVSDRRGNWNLGWDIGNLDLSILVAYKLKMNWGARI
RLITVVNNPDEEENAKEFLNTLTSLARLPQTMTEVYVGDFYTVVKNAPNADLNIFGMDEDLKFEFVKEMSHKTKSSCLFV
RDSGHESILA
>WP_282160768.1 Ulvibacterium marinum
MKNQNQQLKKFGTFGGVFTPTLLTILGVIMYLRMGWVVGNAGLLGAWLIILVSFTITLTTALSMSAITTNIRIGAGGAYA
IISQALGLEVGGSLGIPRYISQGLAVTMYIFGFREGWLGIFPGHSAFLVDIVVFVVLLAIAYISADLAIKTQFFIMLVIV
LSLISIVIAAYYGSMYLPTDEAVRWGTFKGSPENGFSGSDFWIVFAVFFPASTGIMAGANMSGELKNPKRSIPLGTLWAI
GVSFVIYMGLAYWLARSASEEELISNYNIIIEKAYVGPLVIAGILGATFSSALASMVGSSRILFAMGEHRVLPYAKFLAG
QSANGQPRNAMIVTGIMIFATMLLRDLNSVAPLVTLFFLITYAMINIVVIIEQNLGLISYRPIFKIHKWVPWLGLLSSVL
AMFIINPTISLISISIVFVVYWFLSRQNLETPFEDMRSGLFVSFAEWAAKHTWGMKSMQQRAWKPNLMVPIRDIKGAKGN
FQFLRNIARPKGSIKLLGIAPFTESNSFTDEISQLSEAFYKKGVFSSWTVIHTKDFAQGVNYGNQALRGAFFRPNIVFLN
LQQHDNYETELRPVIKECIRLEVGVLLYQAHPTALLGQRNIINVWVSDRKGNWSLGWDIGNLDLSTLIAYKLKRNWEAQI
RLITVINDEEEYENAKEFLNSLINLARLPETLTEIHIGNFRDVVGKASNADLNIFGMHADLPFEFVKEMTQKTESSCLFV
KDSGHESILA
>WP_161436312.1 Poritiphilus flavus
MPQKKVKQFGTFAGVFTPTLLTILGVIMYLRMGWVVGNAGLLGAWLIIIISFAITLSTALSMSAITTNIRIGAGGAYAII
SQALGLEVGGSLGIPRYVSQGLAVTMYIFGFREGWLGIFPDHSPFLIDIIVFAVLITIAYISADLAIKTQFFIMIVIILS
LLSIVLAAYHGSMSVPTEEAIRWGTFKGSVENGFSGSDFWLVFAVFFPASTGIMAGANMSGELKDPKKSIPVGTLWAIGV
SFVIYMSLAFWLARSASEQELISNYNIVIDKAYVGPLVIAGILGATFSSALASLVGSSRILYAMGEHRVLPYSKVLAGTS
ANGQPRNAMIVTGIMIFVTMLLRDLNAVAPLVTLFFLITYAMINIVVIIEQKLGLISYRPIFKVNKWIPWIGLVSSVVAM
FIINPTVSLISISIVFVVYWFLSRQNIETPFEDVRSGLFVSFAEWAAKHTWGMKNMQQRAWKPNLMVPIRDIDGARGNFQ
FLRNIARPKGSIKLLGIAPNVENSVFPDELATLSESFRQKDVFSSWTVIHSENFAQGVNYGNQALKGAFFRPNIVFLNLQ
QHDNYETEIRPVIQECIRLEIGVLLFSLNSTTLLGQRNVINVWVSDRTDNWNLGWDIGNLDLSTLIAYKLKKNWEAQIRL
IIVIRDEKEKENAHNFLQSLTSLARLPSTLTEVHIGSFREVVQKAPKADLNIFGMDGNLKFEFVNEMTSKTGSSCLFVKD
SGHESILA
>MFK2820336.1 Flavobacteriaceae sp.
MSKTANHIKKFGTFGGVFTPTLLTILGVIMYLRMGWVVGNAGLLGTWLIIIISFGITLCTALSMSAITTNIRIGAGGAYA
IISQSLGLEVGGSLGIPRYISQGLAVTMYIFGFREGWLGIFPAHDPFLVDLVVFLVLISIAYISANLAIKTQFVIMVIIV
LSLVSIVMAAYNGSMQIPVTEAISWGEFKGSPENNFSGSNFWIVFAVFFPAATGIMAGANMSGELKDPKRSIPQGTLWAI
GVSFVIYMVLAFWLARTATQEELLTNYNIIIDKAYFGPLIIAGILGATFSSALASIVGSSRILFAMGEHQVLPYSKFLAG
QSANGQPRNAMLVTGVLIFATMLLRNINAIAPLVTLFFLVTYAMINIVVIIEQRLGLISYRPLFKVNKWVPRLGLISSVL
AMFIINPTISLVTIMIVFVVYWFLSRQNLETPFEDVRSGLFVSFAEWAAKHTWGMKSMQQRAWKPNLMIPIRDLNGIKGN
FQFLTNIAYPKGSLKLLGIEPDGKNNALLDELENLSKSFREQGVFSSSTVINTSEFAKGVNYGNQALRGAFFRPNIVFLN
LQQHDDFENEIRPVIHESIRLEIGVLLFLAHPTAALGQRNIINVWVSDRTNNWSLGWDIGNIDLSALIAYKLKLNWNAQI
RFITVIKDNSEKENAEKFLNKLIHLARLPKTQTKVYVGNFMTIVQEAPAADLNIFGLNEDLKEEFIHGMTKHTNSSCLFV
KDSGHESILA
>WP_342158407.1 Flavobacteriaceae sp.
MNNEPKSIKKFGTFGGVFTPTLLTILGVIMYLRLGWVVGNAGLLGAWLIIVISFLITLCTALSMSAITTNIRIGAGGAYA
IVSQALGLEVGGSLGIPRYISQGLAVTMYIFGFREGWLGIFPDHNAFLVDIIVFTVLFTIAYISANLAIKTQFIIMGIIV
LSLISIVAAAYNGSMQIPTSEALRWGSFKGSIENGFTGSNFWVVFAVFFPAATGIMAGANMSGELEHPKRSIPSGTLWAI
GVSFVIYMLLAFWIARSASESELLNDYYIMVDKAYYGPMIIAGILGATFSSALASIVGSSRILFAMGEHKVLPYSNFLHG
QSKNGQPRNAMLVTGILIFLTLLLRNINAVAPLVTLFFLITYAMINIVVIIEQNLGLISYRPVFKIHKWVPWFGLLSSVF
AMFIINPSVSLISIMIVLMVYWYLSRQNLETPFEDVRSGLFVSFAEWAAKHTWGMKKMQQRAWKANLMVPVRDVSGLKGS
FEFLRNIAKPKGSIKLLGIEPFSETSTLAEELEGISAAFRQKEVFSSSSVIHTDEFAKGINYGSQALQGAFFKPNIVFLN
MQDHDDYENELRPVMKESIRLEIGVLLYLSHPTALLGQRNTINVWVSDRKGNWNLGWDIGNLDLSILVAYKLKMNWGARI
RLITVVNDPEEEENAKDFLKTLTNLARLPKTLREVFVGDFYEVVKKAPTADLNIFGMDEDLKFEFVKEMSVKTSSSCLFV
RDSGHESILA
>MBT8257378.1 Bacteroidia bacterium
MNGQTKQIKTFGTFGGVFTPTLLTILGVIMYLRMGWVVGNAGLLGAWLIIIISFLITLCTALSMSAITTNIRIGAGGAYA
IISQALGLEVGGSLGIPRYISQGLAVTMYIFGFREGWLGIFPAHDPFLVDLAVFGVLITIAYISANLAIKTQFIIMGVIV

LSLISIVLAAYHGSMEIPLQDAVKWGTFKGSVENDFGGSSFWVVFAVFFPAATGIMAGANMSGELKDPKRSIPVGTLWAI
GVSFIIYMILAFWLAKTATEEELLTNYNIIVEKAFNGPLIIAGILGATFSSALASLVGSSRILYAMGEHRVLPYSKFLAG
TSKSGQPRNAMIVTGILIFATMLLRNINAVAPLVTLFFLVTYAMINIVVIIEQNLGLISYRPLFKINRWIPWMGLISSIF
AMFIINPTVSLITVVIVLVVYWFLSKQNLETPFEDVRSGLFVSFAEWAAKHTWGMKSMQQRAWKPNLMVPIRDINGANGN
FQFMRNIARPRGSIKLLGIEQFSEDSELVNELDDMSEAFRNQGVFSSWTIINTDEFAKGVNYGNQALKGAFFRPNIVFLN
LQDHDNYETEIQPVIKECIRLEIGVLLYLAHPTALLGQRNMINVWVRDRENNWNLGWDIGNVDLSTLIAYKLKLNWDAKI
RLITVIRDPKEELQAREFLQSLVTLARLPKTLVEVHVGDFRTIVNQAPVADLNIFGMEENLRFDIIKEISKSTNSSCLFV
KDSGHESILA
>WP_321250689.1 Psychroserpens sp.
MSNQTKALKTFGTFGGVFTPTLLTILGVIMYLRLGWVVGNAGLLGAWLIIIMSFLITLCTALSMSAITTNIRIGAGGAYA
IVSQALGLEVGGSLGIPRYVSQGLAVTMYIFGFREGWLGIFPDHNAFLVDVIVFGVLFTIAYISANLAIKTQFIIMGIIV
LSLISIVIAAYEGSMTLPTSDALSWGSFRGSPENGFSGGSFWMVFAVFFPAATGIMAGANMSGELKDPKRSIPSGTLWAI
GVSFIIYMALAFWISRSASESELISNYYIMVDKAYFGPMIIAGILGATFSSALASIVGSSRILYAMGEHKVLPYSDILSG
KSKNGQPRNAMIVTGILIFLTLLMRNLNAVAPLVTLFFLITYAMINIVVIIEQNLGLISYRPVFKIHRWVPWLGLISSVF
AMFIINPTVSLISIMIVLTVYWYLSRQNLETPFEDVRSGLFVSFAEWAAKHTWGMKKMQQRAWKANLMVPVRDVNGLKGT
FEFLRNITKPKGSIKLLGIEPFTEHSKLADQLDTISASFREKGVFSSSTVIHTEEFANGINYGSQALQGAFFRPNIIFLN
LQDHDDYEYELKPVMNESIRLEIGVLLFSLHKTALLGQRNTINVWVSDRRGNWELGWDIGNLDLSILIAYKLKMNWNAQI
RLIMVTEHEEDIEKATDFLDRLINLARLPQTMTEVRLGKFIDIVQDAPSADLNIFGMEPNLKYDFIKDMTIKTSSSCLFV
KDSGHESILA
>MBT8203974.1 Eudoraea sp.
MAKPTQNLKKFGTFGGVFTPTLLTILGVIMYLRLGWVVGNAGLLGALLIIAVSFLITLTTALSMSAITTNIRIGAGGAYA
IISQALGLEVGGSLGIPRYISQGLAVTMYIFGFREGWLGIFPGHNPFLVDIIVFGVLVTIAYISADLAIKTQYLIMGVII
LSLISIVMAAYYGSMYIPTEEALKWGSFKGSPENDFGGSTFWLVFAVFFPASTGIMAGANMSGELKDPRKSIPVGTLWAI
GVSFVIYVFLAFWISRSATEAELVSNYNILIDKAFFGPLVIAGILGATFSSALASIIGSSRILYAMGEHRVLPYGKFLAG
TSANGQPRNAMIVTGIMIFATMLLRDLNAVAPLVTLFFLITYAMINIVVIIEQNLGLISYRPIFKVNRWIPWVGLVSSVL
AMFIINPTISLASFSIVFVVYWFLSRSNIETPFEDVRSGLFVSFAEWAAKHTWGMKTMQQRAWKPNLMVPIRDINGAKGN
FQFLRNIARPRGSIKLLGIEPFKENSPLVQELDTLSEAFRDKGVFSSWTVIHSDNFAQGVNYGNQALRGAFFKPNIVFLN
LQQHDNYETEIRPVIKECIRLEIGVLLYSAHPTALLGKRNMINVWVSDRTNNWNLGWDIGNLDLSTLIAYKLKRNWQATI
RLIIAIKDPKEEKNAREFLDSLVSLARLPKTLTEVHVDDFRSVVAKAPPADLNIFGMDGNLRFEFVQEMTEKTNSSCLFV
RDSGHESILA
>WP_371002241.1 Muriicola sp.
METNATRLKKFGTFGGVFTPTLLTILGVIMYLRLGWVIGNAGLLGAWLIIIISFAITLCTALSMSAITTNIRIGAGGAYA
IVSQALGLEVGGSLGIPRYVSQGLAVTMYIFGFREGWLGIFPDHNPFLVDVLVFGILFGIAYVSANLAIKTQYIIMCVIV
LSFVSIIWAAYEGSMFQANEDVLRFGSFKGSVENNFSGSNFWIVFAVFFPAATGIMAGANMSGELKDPKKSIPVGTLWAI
GVSFVIYMALAYWLSRSATEAELISDYYVIVEKAAFGPLIIAGILGATFSSALASIVGSSRILFAMGEHKVLPYSKFISG
QSANGQPRNAMIITGILIFATMLLRNINAIAPLVTLFFLITYAMINIVVIIEQNLGLISYRPVFKIHRWVPWIGLISSVF
AMFIINPTVSLLSVLIVFVVYWYLSRQNLETPFEDVRSGLFVSFAEWAAKHTWGMKKMQQRAWKPNLMVPVRDINGAKGI
FEFLRNIAKPKGSIKLLGIEPYSEKSTLARQLGNLSESFRTKGVFSSWTVIHTEEFTKGINYASQALQGAFFKPNVVFLN
LQEHDDYEKEVRPIIKESIRLETGVLLYVSHPTALLGERNMINVWISDRQDNWNLGWDIGNLDLSLLIAYKLKTNWKAQI
RLITVINNPKEEENAKNFLNSLINLARLPMTKTEVFVGEFKTIVENAPSADLNIFGMEENLSFRFVKEMVYKTNSSCLFV
KDSGHESILA
>WP_348370759.1 Maribacter sp.
MEQPSNAPKKFGTFGGVFTPTLLTILGVIMYLRLGWVVGNAGLLGAWLIIIISFLITLCTALSMSAITTNIRIGAGGAYA
IVSQALGLEVGGSLGIPRYISQGLAVTMYIFGFREGWLAIFPDHNAFLVDVAVFALLFSIAFISANLAIKAQFVIMGIIL
LSLISIVIAAYQGSMTIPTSEALSWGSFKGSVENEFSGSNFWVVFAVFFPAATGIMAGANMSGELKDPKRSIPTGTLWAI
GVSFMIYMVLAFWISRSASESELVNNYYIMVEKAYIGPLILAGILGATFSSALASIVGSSRILFAMGEHKVLPFSDFLAG
HSKNGQPRKAMLVTGILIFATLLLRNLNAVAPLVTLFFLITYAMINIVVIIEQKLGLISYRPVFKIHRWVPWFGLVSSVF
AMFIINPTVSLISIMIVLMVYWYLSRQNLETPFEDVRSGLFVSFAEWAAKHTWGMKKMQQRAWKANLMVPVRDVEGLKGT
FEFLRNIAMPKGSIKLMGIEAFSEASTLADALEGVSASFRQKGVFSSSSVIHTEEFAKGINYGNQALQGAFFRPNTVFLN
LQDHDDYETELRPVMKEAIRLEIGVLLFLSHPIALLGQRNIINVWVSDRKTQWDLGWDIGNLDLSILIAYKLKMNWGARI
RLITVINDENEAANAQDFLSLLINLARLPETLTEVIVGDFNEIIQNAPAADLNIFGMHDDLKFEFVKAVSAKTKSSCLFV
KDSGHESILA
>WP_266012388.1 Lentiprolixibacter aurantiacus
MANPTNNLKKFGTFGGVFTPTLLTILGVIMYLRMGWVVGNAGLLGAWLIIAISFLITLTTALSMSAITTNIRIGAGGAYA
IISQALGLEVGGSLGIPRYVSQGLAVTMYIFGFREGWLGIFPDHNPFLVDIIVFTVLIGIAYISADLAIKTQYIIMGIII
LSLISIVMAAYYGSMYIPTDEALKWGTFKGSPENNFGGSNFWLVFAVFFPASTGIMAGANMSGELKEPRKSIPVGTLWAI
GVSFVIYILLAFWIARSASEAELISNYNILIDKAYFGPLVIAGILGATFSSALASIIGSSRILYAMGEHRVLPYGKFLAG
TSANGQPRNAMIVTGIMIFITMLLRDLNAVAPLVTLFFLITYAMINIVVIIEQNLGLISYRPIFKVNRWIPWVGLVSSVL
AMFIINPTISLASFSIVFIVYWFLSRANIETPFEDVRSGLFVSFAEWAAKHTWGMKSMQQRAWKPNLMVPIRDIHGAKGN
FQFLRNIARPKGSIKLLGIEPFVENSPLVQDLDTLSEAFRNQGVFSSWTIIHSEDFAQGVNYGNQALRGAFFKPNIVFLN
LQQHDNYETEIRPVIRECIRLEIGVLLYSAHPTALLGKRNMINVWVSDRSNNWNLGWDIGNLDLSTLIAYKLKRNWQATI
RLIIAIRDPQEEQNAREFLDSLISLARLPQTLTAVYVEDFHSVVSKAPPADLNIFGMDGDLRFEFVQEMTEKTGSSCLFV
KDSGHESILA

>AQK93276.1 Zea mays
MTSMEPGSSSSTIDAVVTPQPPRNLKPDANLTIDPSMREGSPDDHATSSGSQGDSKLELFGFDSLVNILGLKSMTGEQIQ
APSSPRDGEDVAITIGRPKESGPKFGTMMGVFVPCLQNILGIIYYIRFTWIVGMSGVWQSLVLVSFCGACTFLTSISLSA
IATNGAMKGGGPYYLIGRALGPEVGVSIGLCFFLGNAVAGSMYVLGAVETFLDAVPSAGLFQKSVTVVNNTLVNGTETAG
TSTISTPSLHDLQVYGVIVTILLCFIVFGGVKIINKVAPAFLIPVLFSLLCIYLGVFIAPRHNAPKGITGLSITTLKDNW
GENYQRTNNAGVPDPSGSIYWDFNALVGLFFPAVTGIMAGSNRSASLKDTQRSIPIGTLSATLSTTAMYLFSVLLFGALA
TREELLTDRLLTATVAWPAPAVIYIGIILSTLGAALQSLTGAPRLLAAIANDDILPVLNYFKVSEGSEPHAATLFTAFIC
ICCVVIGNLDLITPTITMFFLLCYAGVNLSCFLLDLLDAPSWRPRWKFHHWSLSLVGALLCVASSITM
>VAI21230.1 Triticum turgidum
MLHRVDLKGIQSWSFLGLIHLLIFWDSRGEQTQAPSSPREGEDVAITIGRPKETGPKFGTMMGVFVPCLQNILGIIYYIR
FTWIVGMAGIWQSLVLVSFCGACTFLTGLSLSAIATNGAMKGGGPYYLIGRALGPEVGVSIGLCFFLGNAVAGSMYVLGA
VETFLDAVPSAGLFQESVTVVNNTLINGTATAGTATISTPSLHDLQVYGVIVTILLCFIVFGGVKIINKVAPAFLIPVLF
SLLCIYLGVFIAPRHNAPKGITGLRLTSLRDNWGSEYQRTNNAGVPDPNGSIYWDFNALVGLFFPAVTGIMAGSNRSASL
KDTQRSIPIGTLSATLTTTVMYLLSVLLFGALSTREELLTDRLLTATVAWPAPVVIYIGIILSTLGAALQCLTGAPRLLA
AIANDDILPVLNYFKVSEGVEPHAATLFTALICIGCVIIGNLDLITPTITMFFLLCYAGVNLSCFLLDLLDAPSWRPRWK
YHHWSLSLVGALLCVGTLFASILNFD
>XP_019095235.1 Camelina sativa
MTGEQIPTPSSPRDGEDISITQGHPKPLKMGTMMGVFVPCLQNILGIIYYIRFTWIVGMAGIGQGLILVLLCGLCTFLTT
ISLSAIATNGAMKGGGPYYLIGRALGPEVGISIGLCFFLGNAVAGALYVLGAVETFLKAFPAAGIFRETITKVNGTAVAE
SIQSPNSHDLQIYGIVVTILLCFIVFGGVKMINRVAPAFLVPVLLSIFCIFIGIFLAKTDDPDSNFTGLRLKSFKDNWGS
AYQMTNDAGIPDPTGGTYWSFNELVGLFFPAVTGIMAGSNRSASLKDTQRSIPVGTLAATLTTTSLYLISVLFFGAVATR
NKLLTDRLLTATIAWPFPAIVHVGIILSTLGAALQSLTGAPRLLAAIANDDILPILNYFKVADTSEPHIATLFTAFICIG
CVVIGNLDLITPTVTMFYLLCYSGVNLSCFLLDLLDAPSWRPRWKYHHWSLSFVGASLCIVIMFLISWSFTVVAIALASL
IYKYVGLKGKAGDWGDGFKSAYFQLALRSLRSPGAT

>XP_014421908.1 Camelus ferus
MALFEEEMDSNPMVSSLLNKLANYTNLSQGVVEHEEAEDSRRREVQGPRMGTFIGVYLPCLQNILGVILFLRLTWIVGAA
GVLESLLIVSMCCTCTMLTAISMSAIATNGVVPAGGSYYMISRSLGPEFGGAVGLCFYLGTTFAGAMYILGTIEIFLTYI
SPSAAIIQAEAADGEAAAMLHNMRVYGTCTLALMAMVVFVGVKYVNKLALVFLACVVLSILAIYAGVIKTAFDPPDISVC
LLGNRTLSRRGFDVCAKVQAANNGSMATALWGLFCNSSTASTSCDQYFLQNNVTEIQGIPGVASGVLLDNLWSAYADKGA
FVEKKGLPSVAVPEDSGAGGPPYVLTDIATCFTLLVGIYFPSVTGIMAGSNRSGDLKDAQKSIPMGTILAIVTTSFIYLS
CIVLFGACIEGVVLRDKFGEALQGSLVIGALAWPSPWVIVIGSFFSTCGAGLQSLTGAPRLLQAIARDGIVPFLQVFGHG
KANGEPTWALLLTALICETGILIASLDSVAPILSMFFLMCYMFVNLACALQTLLRTPNWRPRFKYYHW
>XP_013306057.1 Necator americanus
MVKESVLSQVSKKESEVKFGWIEGVFVRCLQNIIGVILYLRLTWVAGQAGICMFLVYISRTLGAEFGGSIGLIFCLANCV
GGALYVVGFAETISHLMDGYGITIIDGGVWDVRIISLATCTTLVALICVSATIESKLQQVLLVPLFLSVLSFVVGSFIWT
EKKERYGYTGYQADTLAANMWPDFRQGHSFFSIFSVYFPAATGIMAGANISGNLKNPQDAIPRGTLSAILVSTIIYMSVL
TIAGATYVRDADGEIPLNPLSTPDCCYNYSCPFGLLNYYQIVMVTSVWPPLITVGIVAATLCSALASLISAPKIFQVRGI
FVVYLIAKGISHVITIIFKKTAVEYNDKDPEIEDKPQQQLELQGELVASSHCESDEDLLAPNYFQAICEDKVIPAVHFFA
KGYGRGNDPRRAYLLAFFVTSAVLMIGRLLINQCKRVEVVEQSYGDYSSELNYIAPFISNFFLCSYALLNYSCFSASFSQ
SPGFRPAFRYYSHWLSLLGAVMCVAIMVIMSWLTTLLTFLFFLTVYAFIKHLKP
>VDO18867.1 Heligmosomoides polygyrus
MTDSPNGAGNHAVVFTQRDSQDEDDAKENGFQSQDQPAPRSRGLQSDELVEGQIEKPSKVRVEKFNPPPTQTRVKFGWIQ
GVFVRCLLNIFGVMLYLRISWVAGQAGVGLGSMIVLLASLVTSITAISTCAICTNGDVKGGGAYFLISRSLGPEFGGSIG
LIFSVANAVGAAMYVVGFAETVRDLLKEAGLRIIDSGLWDVRIVGFASCIVLMAIVFIGTEFESKMQMGLLVILVASIID
YMIGSVFPPNENMALRGATGYSLNTLVENLPPAFRGEDFFSVFAVYFPAATGIMAGANISGDLADPQGAIPIGTLLAIAV
TTAIYLATVWMTGASCVRDADGIFPPAWNATVFVPPDCAANYTCPYGLMNYFQIMEMESLWGPLITAGIFAATLSSALAS
LAVCKDHLFPKIGYFAKGYGKNEEPRRAYALTFIIAMAMIGIGDLNAIAPIISNFFLASYALINYACFDASFADSPGFRP
AFKYYNMWVSLAGALLCIVVMFIISWATALITFFCFAALFLYILHRKPDVNWGSSTQAHSYKNALSGMIKLANTEEHVKN
YR
>OTF78144.1 Euroglyphus maynei
GKIVIQKAGAEQEPQESKAVKFGWIKGVLVRCLLNIWGVMLFLRLSWVAAQSGIIHGTIILLLASVVTIVTTMSMSAICT
NGEVKGGGTYYMISRSLGPEFGGSIGIIFSLANAVAVAMYTVGFAETVRDHLGQYGKMIFDGGLNDIRLISSLTVIVLLA
IVYIGTEWEAQTQVFLLFILLTAMTDFMIGAFLPPTDSQISRGYIASLIWENMFPAYRNESFFSVFSVYFPAATGILAGA
NISGDLKDPSYSIPLGTFLAIIITTISYVGFHFMVAANVVRDANGVVELVHHPGVKDVVQAIRNCSLSPDEICHYGSMNN
FQIIELMSWFGPIIYAGIYAATLSSALASMVSAPKVFQALCNDKLFPGIEYFGKGFGVNNEPRRGYILTFLIGLGCCLMG
ELNMIAPIISNFFLAAYTLINFSCFHASWSKSPGFRPSFKYYNLWIALCGSFLCLGVMFMTGWIPALLTFIIVFALHVWV
QRRATGKDFFFRCCC
>XP_014040925.1 Salmo salar
NGRISVTNTVEDREGSDGTPSDDVESVIPLDNKDGTVRFGWIKGVLVRCMLNIWGVMLFIRLSWVFGQAGWGLGIVVIAL
SCVVTTVTGLSMSAICTNGVVRGGGAYYLISRSLGPEFGGSIGLIFAFANAVAVAMYVVGFAETVVDLLKENDAIMIDEL
NDIRIIGCISVVLLLGISVAGMEWEAKAQIGLLIILLVAIANVFVGTGIPASTDKKSKGFFNYDAKIFMENLPPDFRDGE
TFFSVFAIFFPAATGILAGANISGDLKDPQDALPKGTLLAILITGVTYLGVALCVMTMVSGFGPLITAGTFSATLSSALA
SLVSAPKVFQALCKDNIYTALKFFAKGHGKNNEPIRGYVLTFIIAVAFIIIADLNVIAPIISNFFLASYALINFSCFHAS
YAKSPGWRPAYRYYNMWLSLFGAVLCCGVMFVINWWAALLTYAIEIFLYVYVTVKKP
>POI32771.1 Bambusicola thoracicus
EPFEDGYANGEEGTPAGDAAATYTPDSKGIVKFGWIKGVLVRCMLNIWGVMLFIRLSWIVGQAGIGLSVVVIAMATVVTT
ITGLSTSAIATNGFVRGGGAYYLISRSLGPEFGGAIGLIFAFANAVAVAMYVVGFAETVVELLKENGTLMIDEMNDIRII
GAITVVILLGISVAGMEWEAKAQIVLLVILILAIGDFVIGTFIPLDSKKAKGFFGYKAEIFMENFGPDFREETFFSVFAI
FFPAATGILAGANISGDLADPQSAIPKGTLLAILITTLVYMGIAVSVGSCVVRDATGNINNTIITELTNCTTAACKLNYD
FSSCQTGCHYGLMNNFQALCKDNIYPGFQMFAKGYGKNNEPLRGYLLTFLIALGFILIAELNVIAPIISNFFLASYALIN
FSVFHASLAKSPDVNWGSSTQALTYLNALQHSIRLSGVEDHVKNFSFEFQYHGDCLDVAT
>XP_014878990.1 Poecilia latipinna
LRQPTQSCNIQSFKELRADLINPRGPATEELFNHLGNLVLRDWRVRPRVPRLSFLNGWHVGGFEEVFEVFRPPAHKVLSQ
GQQQTIPTINSEPFEDGFPNGDELSPAEEAAAKEAAEPKGVVKFGWIKGVLVRCMLNIWGVMLFIRMTWIVGQAGIALAC
VIVGMATVVTTITGLSTSAIATNGFVRGGGAYYLISRSLGPEFGGSIGLIFAFANAVAVAMYVVGFAETVVELLKGVDAL
MTDDINDIRIIGTITVIVLLGISVAGMEWEAKAQIFLLVVLITAIVNYFIGSFISVELKKPSGFLGYDAEIMWENMGPDF
RDETFFSVFAIFFPAATGILAGANISGDLADPQMAIPKGTLLAILITGIVYLGVAVSTGSCIVRDASGNLNDTIGTQFSA
NCTGASCKFGFDFSSCKSQKNCRYGLHYDFQVMSLVSGFSPIISAGIFSATLSSALASLVSAPKVFQALCKDNIYPYIGI
FAKGYGKNNEPLRGYILTFGIALAFILIAELNTIAPIISNFFLASYALINFSVFHASLANSPGKIKVQKVKTSIKTC
>RME67410.1 Verrucomicrobiota bacterium
MVRLPMATASAAEKTAPVKFGTFGGVFTPNVLTILGVIMFLRTGWVVGNAGLKQALIILCIANVITLLTSLSLSAVATNI
RVKGGGAYFLISRNLGLEVGGAIGLPLFLAQAVSVAFYIVGFIESVRFLVPDIPAREVSMGVLAVLLVISWVGAGLAVKT
QYAILTALGLSLLAFFAGWSPQPGFVERLEPAYVPGHNFWSVFAIFFPAVTGIMSGVSMSGDLRDPSRSIPKGTILAVLV
TWAIYALQMFWLAWNANRQELVENTLVMQRISRVPFLIFVGLWAATLSSALASLLAAPRTLQALASDGVVPRWMGRGSGK
EKEPKLALVLTAILAGACLLIGELDLIAPIISMFFLTTYGTVNLVAGLSGLVSNPSYRPTFKVHWS
>PKN36275.1 Deltaproteobacteria bacterium
MNETAQSNALSKKFGTFGGVFTPSLLTILGVIMFLRFATVVGYAGLWNTLAILAGAKAISVITGMSISSVATNMRVRGGG
AYFLISRSLGVEFGGVIAVFFYVAQAVAVTLYVVGFTEALFSAFPDLPLTFSAVATITNCLVFASVYIGAGWTIRLQYAI
LAVLLVSVASFLAGALDAGSLQTLQGNLSPNWQPGYTFFPVFALFFPAVTGIMAGVNMSGDLQNPSASIPRGTFLSIFVS
AAIYLGIAVALAASTPRHELLGDGFVMQERAIWPALVYAGVICATLSSALGSMMGAPRILQAFARDNVFRRLRWFGQGSG
SAGEPRRAIVLTFLISQAGVLAGDLDTIAPVITMFFLLTYGTMNLACFYEGFIRNPSFRPTFRFNHWSVSLLGAVGCLGV
MVLIAPAWAVVSMALAGILYYVIARAEIQVRWGDVDSGLAYHQARKALLKLERERYHPKNWRPSILVL
>TSA54908.1 Planctomycetaceae bacterium
MGQKGNQFSTFAGVFTPSILTILGVIMFLRAGYVVGEAGIINTIFILCVAEVISLLTVISMSAIATNTPVAGGGAYFLIS
RALGPQFGGAIGLALFLAQALSVPFYILGFTNSIVASFPSLSAWFVPIALGTAAILFTVNIISSGFAIRVQYIVMAMLAL
AIVSFLGGAILLFDPALLQANLGSSYTKPTIGFWTVFAIYFPAVTGILAGVNMSGDLKDPARSLVRGTMAAIAVGFVIYL
SEIILCGGSQQRVDLQAQPFEMLVKNALWGTGFLVMGGVFAATISSAVGSFLGAPRVLQALARDRIFPILGIFGHGSKDK
DEPRYGLVLTLGLTLGVILILGGGDSMSAFDMIAAIVTMFFLCTYGMINLAAFVESFGANPSFRPSFRFYHWTTSILGFV
GCLVVMILIDAFAALAAILVIAGIYLFLSRRVFRSAFGDARLGFQYALVVRTLQKLRRMTPHAKNWRPTFLVMAGNTQTH
MAL
>RLD32845.1 Bacteroidota bacterium
MHQRFNFAVFKESPDKKLSTFGGVFTPDVLTILGVIMYLRLGWVVGNAGFLGALAIIALAKSITICTALSMSSITTNIKI
GAGGAFSIITKSLGLEAGGSIGIPFYIAQTLSTALYIVGFKEGWLYIFPDHPPFLVAFIAWLLLMGISFISARFAIRVQY
LIMVIVGLSIFAFLFTRSQPVPSLPMMGEFKDVNFWKVFAIFFPAVTGIMAGANLSGDLKNPRKAIPVGTLSAIGVTFVI
YVAMAWVAARYIPADELRTNQMVMVDYALWGPMVLMGILAATFSSALGSFIGAPRILQALSEQRTVPLYKIFSKKTKNNE
PRNATIFTSLIILVALLAGNLNALASLITMFFLITYGIINLVVFMQQGMKIISFRPTFKIPMFVSFLGAAGSFFVMFLIN
AAFSLVAFATIISLYIWLGRRGLKSESGDIRGGFFLVLAE
>WP_146152808.1 Canobacterium CCP4
MSKSQDSSVILSKGQASQGLGTFGGVFTPSILTILGVIMYLRFGWVVGHVGLWQSLLIVTISTSITLLTGLSISAIATDQ
VVRAGGAYYMISRSLGIETGGAVGIPLFLAQAFSVALYTIGFAESLVQVFPMLSQTLVALITTILVAVLALKSADIAIKA
QYFIMAAIVLSLGSLLLGHSVEPTSLPASESPGTVGFWAVLAVFFPAVTGIMAGVNMSGDLKDPQRSIPAGTLAAIGVGY
IIYMGIPILLSQWADPFTLVAEPLVMRRMARWGDVILLGVWGATLSSAIGSILGAPRVLQALARDGILPAWLRWLGRGNG

PLDEPRLGTLFTLGIALAAVTIGDLNLIAPVLTMFFLTTYLVLNVSAGLETFLQSPSFRPTFKVHWSLSLLGAVGCLVVM
FLINAVATVMAAVIVLGIFVWLQRREMQGAWGDVRNGLWMTLVRTSLFQTVSYTHLRA
>KAA3665132.1 Chloroflexota bacterium
MVSKFLSLFSRNGVAVENGRKFGTFGGVFTPTVLTILGVIMYLRTGQVVGNAGLGGAILIILLAHVITVSTGLAVSSVAT
NIRVGAGGAFSIISQSLGLEVGGGIGIPLYLAQSVSVALYILGFSIAWQGVFPSHNEKIIAIIAFFCVFGIAYISAQFAA
RIQYLILALVAFSLVSVFLGSFPIGEQVGFTEAPQLWGNFQLWSFWETFAIFFPAVTGIMVGISLSGSLQSPRKSLPIGT
MSAIGLTLIVYLLLTFWLSRVATPEELRDNELIMVGKAYWGWAILAGTLGATFSSALGSIVAAPRVMQALAVYGLLPYSN
KLAEVGNDGEPRTAMLATGGISLLTLIFAWLSGGFDAVAPLITMFFLITYGMLNVVVLIEQTLAMVSFRPTFRIPRIVPL
IGTVGCLFVMFLINPMFSLVAAVLVLIIYSFLVRQKLKTLPSDVRSGLFFSIAEWAMKRASRMPAAPERTWKPAVLVPVT
STSELTGSYRFLWAMTSPRGAVQA
>WP_035828674.1 Crocosphaera watsonii
MLLRLKKLIRFLNPLSFNSPTYPPKKYDTFEGVFKPTLLTILGAIMYLRIGWVVGNAGLFGGLAIVLLSVSITLATGLSI
ASIATNTRMGEGGPYAMISKSLGLEIGGSVGLPLFVSQALAAAMYIFGFREGWLFLFRDHSPLVIDLVAFLFIFIIAYIS
AYFAFRVQYLVLILIILSLISIFSSSITWETTGTWQDWGDFPNTNFWGVFAVFFPATTGIMSGVNMSGELKNSRKNIPIG
TLSAIGLSTIVYIILCWWVARAASPQELINNYTILIEKSRWQPLVLMGLLAATFSASLSSLVGAPRILMALAKDGVIPWG
NSLAKLSKNGEPRRALLVSATLVLLALLLRDLNAIAPLITLFFLLTYATINLVVLVESSLSLMNFRPPFRVLIIVPLYGI
IGCVLAMLVIRPFLSLVAITIVFGIYLQLVSVPKEKRSTDV
>RKX39530.1 Verrucomicrobiota bacterium
MGENSEKQGYSFGTFKGVYTPSVLTIFGVIMYLRFGWVLGNVGLAGTLLIVTIATAITFLTGLSISAMATNMKVGGGGAY
YMIARSLGLEAGAAIGLPLFFARAIGVAFYIAGFTEALLSIANPLPFDPAITAKIVSSATLVLLTALAYFSADLALKVQF
GIFAAIGISLVSFFMGNPTVEALAIPPDAIIPPKAGFWVVFAVFFPAVTGIEAGLGLSGDLKNPAKALPLGTLLAIVTGY
LVYMVIPFFLNSKVPDANLLLIDSNIMSTVARWAPLVILGVFAASLSSALGSLLGAPRTLQALAVDRVIPRFIGRGFGKD
KADPRIATFLSFVVALAAVLLGDLNMIAPILSMFFLLSYGLLNLSAGLEGLIESPSWRPRFKV
>KPK46148.1 Nitrospira bacterium
MPEAEKVGYQFGTFKGVFTPNILTILGVIMYLRFGWVLGNVGLVPTLVIVTISTALTFLTGLSVSTLATNMKVKTGGAYY
IISRALGIEAGASIGLPLYMAQTLGISFYAIGFAESVIQIIPYADINIKLIGIVTLAVLTIISLKSTDIALKTQYIILAL
IALSLISFFAGSGERIPPISSDIIVPDKAKFWTVFAIFFPAVTGILSGLSMSGDLKNPEKAIPWGTLASVACSYAIYMAI
PIFFVKYITDHRIFLIDYLIMFKVARWGFFILAGLWGAALSSALASLLSAPRTLQAMAKDGIVFRFLGYGSGKNQEPRIA
VMISFLIALTGILAGDLNIIASILSMFFLTTYGLLNLSAGFGRLINSPSWRPTFRLHWGFSFAGAFGCFACMFMINPGAT
FVAIFIAIGIYTLTKRRRLKTRWGDMKYGILMLLIQFGLYRLSSKKPNIETWRPNILVLSGSPTARWYLIEIANAFSHGY
GLLTVAAVLSDKHISNERKENMEETI
>PLX18294.1 Marinilabiliales bacterium
MQNKSAQLNTFGGVFTPSLLTILGVIMYLRFGWVVGNVGLIGTLIIVTLSTSITFLTALSIAAIATNAPVKSGGAYFMIS
RSLGIEIGGAIGIPLYLAQAFSVALYIIGFSESIVSIFPAVDIKIVGIITTIVLGTLSLFSTKAAIRAQYIILGVIALSL
LSLVFGGPIENSQIEMWGVPSSEAVNFWKVFAVFFPAVTGIMAGVNMSGDLKDPSKSIPKGTFIAVGVGYVIYMTLPMIL
ATRADAKTLVENPMIMQQIAFWGGAILLGIWGATLSSAVGSLMGAPRVLQALTNDKVIPRQFSFLGKGSGKEKIPRWATI
FTIVLTLILVYLGNLNTIAPILTMFFLTTYGILNITAGIEKLVESPSFRPKFKVHWLFSLLGTIGCFAVMFLINPIATII
AIIFISIVYTWLRRRNLKATWGDVRSGMLLQIIRYALLRLEKEASPKSWR
>TVP54884.1 Gemmatimonadales bacterium
MKHPPDTSITDDSAKGTFRGAGLGTFGGVFTPSILTILGVIMYLRFGWVVGNVGLLGTLLIVVMCTSITFLTGLSMASIA
TDRRVGTGGAYFMISRSLGLETGGAVGIPLYLALTLSMALYVIGFAEIVGDVYPDLDPRWVGVVTAIAVAGLAIRSADLV
IRAQYIVMAVIGLSLLSFFFGGPVDDTEIELMATAPDTISVGFWVAFAVFFPAVTGIEAGVNMSGDLKKPSRSIPMGTFA
ALGVGFVVYMTMPFFFSMWADPVTLVEDPFIMRRLAYWGPLILLGAWGATLSSAMGSILGAPRVLQALARDGVLPGPLRI
LGRGSGEDDAPRIGTVVTLALALVAIYLGDLNLVAPILTMFFLTSYMTVNFAAGIEGFLQSPSFRPA

Supplementary Dataset 2

The list of ancestral sequences A24-A37 and their alignment in ProMals3D

>A24
LEAKPPEEEVANESARKKEKGKKFGTFGGVFVPCLLNILGVILFLRFSWVVGQAGIWNTLIILCLASAITLLTGLSMSAI
ATNGRVKGGGAYYLISRSLGPEFGGAIGLCFYLANAVAVAMYVVGFTETILDLFPDLGLLIQDSGLHDLRVVGLVTTVLL
FAIVYIGAGWAIKVQYIILAVLLLSILSFFAGALLPPDEAEALRGFPGYAAGTLLENLWQGYNLSPASRPGYNFFSVFAI
YFPAVTGIMAGANMSGDLKDPQRSIPRGTLLAILVTTVIYLGVVFLLGASAQRQDFACPYELLNDPLVMQKTALWPPLIF
AGVFAATLSSALGSLLGAPRVLQALARDNVFPRLLRWFGRGSGKNNEPRRALILTFLIALACVLIGDLDMIAPIITMFFL
TTYGLINLACFLEGFVRNPSFRPTFKYYHWSISLLGAVLCLGVMFLINWLAALVALLLVAALYLYIARRKLKARWGDARS
GLAYQLARRALLKLERERPHPKNWRPSILVL
>A25
LEQKPPEEEAANEQGRKKEKGPKFGTFGGVFVPCLLNILGVILFLRFSWVVGQAGIWQSLIIVCLASAVTLLTGISMSAI
ATNGRVKGGGAYYLISRSLGPEFGGAIGLCFYLANAVAVAMYVVGFTETILDLFPELGLLIQDGGLHDLRVVGLVTCVLL
FAIVFIGAEWANKLQLILLAVLLLSILSFFVGAFLPPDDAQELRGFPGYAADTLLENLWQDYNLSPAGRPGYSFFSVFAI
YFPAVTGIMAGANMSGDLKDPQRSIPRGTLLAILVTTVIYLGVVLLLGASVQRADFSCPYELLNDPLVMQKTALWPPLIY
AGIFAATLSSALASLVGAPRVLQALARDNIFPRLQYFGKGYGKNNEPRRALILTFLIALACVLIGDLDMIAPIITMFFLA
SYALINLACFLESFVKSPSFRPTFKYYHWSISLLGAILCLGVMFLINWLAALVALLLVAALYLYIGRRKP
>A35
LRQPTQSCNIQSFKELRAMTASPPGAATEELFNHLGNLVLRDWRVRPRPPRLLLPDGWHVGNFAVVFALREGEADDNAKA
NGRQGQGPLELFGLPSLLNRLALADGLLEGQMARPKKPLPDEELANESAQKKQKGKKFGTFGGVFTPSLLTILGVIMFLR
FGWVVGNAGLWNTLLILCLAKAITLLTGLSMSAIATNMRVKGGGAYFLISRSLGPEFGGAIGLAFYLAQAVAVALYVVGF
TESILSAFPDLPLLILEEITLVNGTAVNGTETAGADGEADSGDHDFRVVGLVTTVLLFAIVYIGAGWAIRVQYVILAVLL
LSILSFFAGALAAPDELETLGITGLGNRTLSRRGFDVCAKVQAANNGSMATALWGLFCNSSTASTSCDQYFLQNNVTEIR
GFPGYGAGTLLENLWQGYAGKGAFTENKGLPSVAVPEDSGAGGLPSPNWQPGYNFFSVFAIFFPAVTGIMAGVNMSGDLK
DPSRSIPRGTLLAILVSAVIYLGIAFLLAASVAQRQDGLLPLALGAGVKDVVLLLPDCTGAACKLGYDFSSCRAAVLAAN
FACPYGELLEDPLVMLQKTALWGTPPLIFAGVFAATLSSALGSLLGAPRVFQVRGIFVVYLIAKGISHVITIIFKKTAVE
YNDKDPEIEDKPQQQLELQGELVASSHCESDEDLLAPNYLQALARDNVFPRRLRWFGRGSGKNKNEPRRALVLTFLIALA
CLLIGVLILNQCKRVEVVEQGGGDSLGDLDMIAPIITMFFLTTYGMINLACFLEGFVRNPSFRWGPTFKFYHWSVSLLGA
VGCLGVMDHVKLFSFLINPLAALVAIVLVAGIYFFIARRELKARWGDVRSGLAYQLARRALLKLERERPHPEKNWRPSIL
VLAGSTQTLTGSYRFHWALIEPAGAFSHGYGLLTVAAVLSDKHISNERKENMEETI
>A36
ANESAQKKQKGKKFGTFGGVFTPSILTILGVIMFLRFGWVVGNAGLLNTLLILCIAKAITLLTGLSMSAIATNMRVKGGG
AYFLISRSLGPEFGGAIGLALYLAQAVSVALYIVGFTESILSVFPDLPLLILFRVVGLVTTVILFAIAYIGAGFAIRVQY
VILAVLALSILSFFAGALPQPDELELLQGYNLSPSSQPGYNFWSVFAIFFPAVTGIMAGVNMSGDLKDPSRSIPRGTLLA
ILVSYVIYLAIAFLLAASAQRQDELLEDPLVMQKTALWPPLIFAGVFAATLSSALGSLLGAPRVLQALARDGVFPRLRWF
GRGSGKENEPRRALVLTFLIALACILLGDLDMIAPIITMFFLTTYGMINLAAFLEGFVRNPSFRPTFKFYHWSVSLLGAV
GCLAVMFLINPLAALVAIVIVAGIYLFLRRRELKARWGDVRSGLAYQLARRALLKLERERPHP
>A37
ANESAQKEQKGKKFGTFGGVFTPSILTILGVIMFLRFGWVVGNAGLLNTLLILCIANAITLLTGLSMSAIATNMRVKGGG

AYFLISRSLGLEVGGAIGLPLYLAQAVSVALYIVGFTESILSIFPARVVGLVTLVILFAIAYIGAGFAIRVQYVILAVLA
LSLLSFFAGAPPEPGQIELLQDYAPSQPGYNFWSVFAIFFPAVTGIMAGVNMSGDLKDPSRSIPRGTLLAILVSYVIYMA
LAFWLAASANRQDELVEDPLVMQRIARWPPLIFAGVWAATLSSALGSLLGAPRVLQALARDGVIPRLRWLGRGSGKDNEP
RLALILTFILALACILLGDLDMIAPIITMFFLTTYGMINLAAGLEGFVENPSFRPTFKVHWSISLLGAVGCLAVMFLINP
IAALVAIIIVAGIYLWLRRRELKARWGDVRSGLLYQLARRALLRLEREKPHP

>A28
SSGSQGDSKLELFGFDSLVNILGLWDMTGEQIQAPSSPRDGEDVAITIGRPKESGPKFGTMMGVFVPCLQNILGIIYYIR
FTWIVGMAGIWQSLVLVSFCGACTFLTSISLSAIATNGAMKGGGPYYLIGRALGPEVGVSIGLCFFLGNAVAGSMYVLGA
VETFLDAVPSAGLFQESVTVVNNTLVNGTETAGTSTISTPSLHDLQVYGVIVTILLCFIVFGGVKIINKVAPAFLIPVLF
SLLCIYLGVFIAPRHNAPKGITGLRLTSLKDNWGSNYQRTNNAGVPDPSGSIYWDFNALVGLFFPAVTGIMAGSNRSASL
KDTQRSIPIGTLSATLTTTAMYLLSVLLFGALATREELLTDRLLTATVAWPAPAVIYIGIILSTLGAALQSLTGAPRLLA
AIANDDILPVLNYFKVSEGSEPHAATLFTAFICIGCVVIGNLDLITPTITMFFLLCYAGVNLSCFLLDLLDAPSWRPRWK
YHHWSLSLVGALLCVATMFTMILNFD
>A27
KLFDSLVNILGLWDMTGEQIQAPSSPRDGEDVAITQGRPKESGPKFGTMMGVFVPCLQNILGIIYYIRFTWIVGMAGIWQ
SLVLVSLCGACTFLTSISLSAIATNGAMKGGGPYYLIGRALGPEVGVSIGLCFFLGNAVAGAMYVLGAVETFLDAVPSAG
LFQESVTVVNNTAVAETISTPSLHDLQVYGVIVTILLCFIVFGGVKIINKVAPAFLVPVLLSILCIYLGVFIAPRDNPDK
GITGLRLTSLKDNWGSEYQRTNNAGVPDPSGSIYWSFNALVGLFFPAVTGIMAGSNRSASLKDTQRSIPIGTLSATLTTT
AMYLLSVLLFGALATREELLTDRLLTATVAWPAPAVIYIGIILSTLGAALQSLTGAPRLLAAIANDDILPVLNYFKVSEG
SEPHAATLFTAFICIGCVVIGNLDLITPTITMFFLLCYAGVNLSCFLLDLLDAPSWRPRWKYHHWSLSLVGALLCVAIMF
LISWNFT
>A26
PLLSSLLNKLALYDNLGEQIQGPRAEEEVAEDSGRRKEQGPKFGTFMGVFVPCLQNILGVILFLRFTWIVGAAGIWQSLL
IVSLCGACTLLTAISMSAIATNGAVKGGGSYYLISRSLGPEFGGAIGLCFYLGNAVAGAMYVLGAVETFLDAIPPAGAII
QEEAADGEASPALHDLRVYGIVTTVLLCLIVFVGVKFVNKLALVFLACVLLSILAIYAGVFLAPRDPPDIGICGQGIPGL
RAGTLLDNLWSGYQDTENKGPPDPLGDIAWSFTALVGIYFPAVTGIMAGSNRSGDLKDAQRSIPLGTLLAILTTTFIYLA
CVLLFGACVERVVLRDKFGELLQDSLVTGTVAWPSPAVIYIGIFLSTLGAALQSLTGAPRLLQAIARDDILPLLQYFGHG
KANGEPRWALLLTALICIACILIGNLDLIAPIITMFFLMCYAFVNLACFLQSLLRTPSWRPRFKYYHWSLSLVGALLCIA
VMFLISWAFA

>A29
VEGQIGKPAEEKAAKEQPPQKESAVKFGWIKGVFVRCLLNIFGVMLFLRLSWVAGQAGIGLGLIIVLLASVVTTITGISM
SAICTNGEVKGGGAYYLISRSLGPEFGGSIGLIFSLANAVAAAMYVVGFAETVRDLLKEYGLLIIDGGLHDIRIIGFVTC
IVLLAIVFIGTEWESKMQMFLLVILLVSIIDFMIGSFLPPDENKEARGFTGYSADTLLENLWPDFRDESFFSVFAVYFPA
ATGIMAGANISGDLKDPQDAIPRGTLLAILVTTVIYLGVVFMVGASVVRDADGVIPPTWNAVVPDCAANYTCPYGLMNNF
QIMEMTSLWGPLITAGIFAATLSSALASLVSAPKVFQALCKDNIFPGIQYFAKGYGKNNEPRRAYILTFLIALACILIGE
LNMIAPIISNFFLASYALINFSCFHASFAKSPGFRPAFKYYNMWISLAGAILCIGVMFIISWLTALLTFLIVLALYLYIQ
HRKP
>A30
VEGQIEKPSKKKAAKAQPPKKQTRVKFGWIQGVFVRCLLNIFGVMLYLRLSWVAGQAGIGLGLMIVLLASVVTTITAISM
SAICTNGDVKGGGAYFLISRSLGPEFGGSIGLIFSLANAVGAAMYVVGFAETVRDLLKEYGLRIIDGGLWDVRIIGFATC
IVLLAIVFIGTEFESKMQMGLLVILLVSIIDFMIGSFLPPDENKALRGFTGYSADTLVENLWPDFRGESFFSVFAVYFPA
ATGIMAGANISGDLKDPQDAIPRGTLLAILVTTVIYLGVVWMTGASCVRDADGIFPPAWNATVFVPPDCAANYTCPYGLM
NYFQIMEMTSLWGPLITAGIFAATLSSALASLVSAPKVFQAICKDNIFPKIGYFAKGYGKNNEPRRAYVLTFLIAMACIL
IGELNAIAPIISNFFLASYALINYSCFHASFAKSPGFRPAFKYYNMWVSLAGALLCIAVMFIISWLTALLTFLIFLALYL
YIQHRKPDVNWGSSTQA
>A31
AEGQEGKPAEQKAAKEQAPQKEKAVKFGWIKGVLVRCLLNIWGVMLFLRLSWVAGQAGIGLGVIIILLASVVTTITGLSM
SAICTNGEVKGGGAYYLISRSLGPEFGGSIGLIFSLANAVAVAMYVVGFAETVRDLLKEYGALIIDGGLNDIRIIGCITV
IVLLAIVFVGTEWEAKAQIFLLVILLTAIVDFMIGSFLPPDDNKKSRGFTGYDADILLENLWPDFRDESFFSVFAVYFPA
ATGILAGANISGDLKDPQDAIPKGTLLAILITTIVYLGVAFMVGASVVRDANGVIPLTHHSGVQAIPNCTAPNETCHYGL
MNNFQIMELVSWFGPLITAGIFAATLSSALASLVSAPKVFQALCKDNIFPGIQYFAKGYGKNNEPRRGYILTFLIALACI
LIGELNVIAPIISNFFLASYALINFSCFHASFAKSPGFRPSFKYYNMWISLAGAILCLGVMFIINWLAALLTFIIVLALY
IYVQHRKPGKDWGSSCCA
>A32
AEGEEGTPADDAAATIQAPDKKGTVKFGWIKGVLVRCMLNIWGVMLFIRLSWVVGQAGIGLGVVIIALATVVTTITGLSM
SAICTNGVVRGGGAYYLISRSLGPEFGGSIGLIFAFANAVAVAMYVVGFAETVVDLLKENGALMIDELNDIRIIGCITVV
LLLGISVAGMEWEAKAQIFLLVILIVAIANFFIGTFIPADTSKKSKGFFGYDAEIFMENLGPDFRDETFFSVFAIFFPAA
TGILAGANISGDLKDPQDAIPKGTLLAILITGVVYLGVALSVGSCVVRDATGNINNTIGTLLSNCTSCHYGLMNNQVMSM
VSGFGPLITAGIFSATLSSALASLVSAPKVFQALCKDNIYPGLQFFAKGYGKNNEPLRGYILTFLIALAFILIAELNVIA
PIISNFFLASYALINFSCFHASFAKSPGWRPSFKYYNMWLSLAGAILCCGVMFVINWWAALLTYAIVLSLYVYVTYKKP
>A33
EPFEDGFANGEEGTPAEDAAATETPDSKGVVKFGWIKGVLVRCMLNIWGVMLFIRLSWIVGQAGIGLSVVIIAMATVVTT
ITGLSTSAIATNGFVRGGGAYYLISRSLGPEFGGSIGLIFAFANAVAVAMYVVGFAETVVELLKENGALMIDEMNDIRII
GTITVVILLGISVAGMEWEAKAQIFLLVILITAIANFFIGTFIPVDSKKAKGFFGYDAEIFMENFGPDFRDETFFSVFAI
FFPAATGILAGANISGDLADPQSAIPKGTLLAILITGLVYLGVAVSVGSCIVRDATGNINNTIGTLLSNCTDAACKFGYD
FSSCRSSCHYGLMNNQVMSLVSGFSPLISAGIFSATLSSALASLVSAPKVFQALCKDNIYPGIQIFAKGYGKNNEPLRGY
ILTFLIALAFILIAELNVIAPIISNFFLASYALINFSVFHASLAKSPGWRPSFKYYNMWASLAGSILCCGVMNFVINWWA
ALLTYAI
>A34
EPFEDGFPNGDEGTPAEEAAAKEAPEPKGVVKFGWIKGVLVRCMLNIWGVMLFIRMTWIVGQAGIALSCVIVAMATVVTT
ITGLSTSAIATNGFVRGGGAYYLISRSLGPEFGGSIGLIFAFANAVAVAMYVVGFAETVVELLKDSGALMIDEINDIRII
GTITVILLLGISVAGMEWEAKAQIFLLVILITAIVNYFIGSFIAVDSKKPSGFFGYDAEILMENFGPDFRDETFFSVFAI
FFPAATGILAGANISGDLADPQMAIPKGTLLAILITGLVYLGVAISAGSCIVRDATGNLNNTIGTQLISNCTDAACKFGY
DFSSCRSTKSCRYGLHNDFQVMSLVSGFSPLISAGIFSATLSSALASLVSAPKVFQALCKDNIYPGIAIFAKGYGKNNEP
LRGYILTFGIALAFILIAELNVIAPIISNFFLASYALINFSVFHASLANSPGWRPSFKYYNMWASLAGSILCCGVMFIIN
WWAALLTNVI

Supplementary Dataset 3

PROMALS3D alignment (sequences in aligned order)

```
Conservation:                                                             6         9           9996  996666 669 966 669  9669 996   5 6   6
A32              1  -----------------------------------------------AEGEEGTP----ADDAAATIQAPDKKGTVKFGWIKGVLVRCMLNIWGVMLFIRLSWVVGQAGIGLGVVIIALA   69
A34              1  ---------------------EPFEDGFP-------------------NGDEGTP----AEEAAAK-EAPEPKGVVKFGWIKGVLVRCMLNIWGVMLFIRMTWIVGQAGIALSCVIVAMA   75
A33              1  ---------------------EPFEDGFA-------------------NGEEGTP----AEDAAAT-ETPDSKGVVKFGWIKGVLVRCMLNIWGVMLFIRLSWIVGQAGIGLSVVIIAMA   75
A31              1  -----------------------------------------------AEGQEGKP----AEQKAAKEQAPQKEKAVKFGWIKGVLVRCLLNIWGVMLFLRLSWVAGQAGIGLGVIIILLA   69
```

```
A29              1  -----------------------------------------------VEGQIGKP----AEEKAAKEQPPQKESAVKFGWIKGVFVRCLLNIFGVMLFLRLSWVAGQAGIGLGLIIVLLA   69
A30              1  -----------------------------------------------VEGQIEKP----SKKKAAKAQPPKKQTRVKFGWIQGVFVRCLLNIFGVMLYLRLSWVAGQAGIGLGLMIVLLA   69
A37              1  ----------------------------------------------------------------ANESAQKEQKGKKFGTFGGVFTPSILTILGVIMFLRFGWVVGNAGLLNTLLILCIA   56
A36              1  ----------------------------------------------------------------ANESAQKKQKGKKFGTFGGVFTPSILTILGVIMFLRFGWVVGNAGLLNTLLILCIA   56
A35              1  ANGRQGQGPLELFGLPSLLNRLALADGLL-------------------EGQMARPKKPLPDEELANESAQKKQKGKKFGTFGGVFTPSLLTILGVIMFLRFGWVVGNAGLWNTLLILCLA  101
A25              1  --------------------------------------------------LEQKP----PEEEAANEQGRKKEKGPKFGTFGGVFVPCLLNILGVILFLRFSWVVGQAGIWQSLIIVCLA   66
A24              1  --------------------------------------------------LEAKP----PEEEVANESARKKEKGKKFGTFGGVFVPCLLNILGVILFLRFSWVVGQAGIWNTLIILCLA   66
A28              1  ----------------SSGSQGDSKLELFGFDSLVNILGLWDMTGEQIQAPSSPR----DGEDVAITIGRPKESGPKFGTMMGVFVPCLQNILGIIYYIRFTWIVGMAGIWQSLVLVSFC  100
A27              1  ------------------------KL----FDSLVNILGLWDMTGEQIQAPSSPR----DGEDVAITQGRPKESGPKFGTMMGVFVPCLQNILGIIYYIRFTWIVGMAGIWQSLVLVSLC   88
A26              1  ----------------------------PLLSSLLNKLALYDNLGEQIQ---GPR----AEEEVAEDSGRRKEQGPKFGTFMGVFVPCLQNILGVILFLRFTWIVGAAGIWQSLLIVSLC   85

Conservation:        6 9 69 6959996996 66999 969969699796966999 7 666995 7697696 96  5  6  76                               7 66 9  6 6 9  9
A32             70  TVVTTITGLSMSAICTNGVVRGGGAYYLISRSLGPEFGGSIGLIFAFANAVAVAMYVVGFAETVVDLLKENGALM-----------------------ID-ELNDIRIIGCITVVLLGI  165
A34             76  TVVTTITGLSTSAIATNGFVRGGGAYYLISRSLGPEFGGSIGLIFAFANAVAVAMYVVGFAETVVELLKDSGALM-----------------------ID-EINDIRIIGTITVILLLGI  171
A33             76  TVVTTITGLSTSAIATNGFVRGGGAYYLISRSLGPEFGGSIGLIFAFANAVAVAMYVVGFAETVVELLKENGALM-----------------------ID-EMNDIRIIGTITVVILLGI  171
A31             70  SVVTTITGLSMSAICTNGEVKGGGAYYLISRSLGPEFGGSIGLIFSLANAVAVAMYVVGFAETVRDLLKEYGALI-----------------------IDGGLNDIRIIGCITVIVLLAI  166
A29             70  SVVTTITGISMSAICTNGEVKGGGAYYLISRSLGPEFGGSIGLIFSLANAVAAAMYVVGFAETVRDLLKEYGLLI-----------------------IDGGLHDIRIIGFVTCIVLLAI  166
A30             70  SVVTTITAISMSAICTNGDVKGGGAYFLISRSLGPEFGGSIGLIFSLANAVGAAMYVVGFAETVRDLLKEYGLRI-----------------------IDGGLWDVRIIGFATCIVLLAI  166
A37             57  NAITLLTGLSMSAIATNMRVKGGGAYFLISRSLGLEVGGAIGLPLYLAQAVSVALYIVGFTESILSIFP------------------------------------ARVVGLVTLVILFAI  140
A36             57  KAITLLTGLSMSAIATNMRVKGGGAYFLISRSLGPEFGGAIGLALYLAQAVSVALYIVGFTESILSVFPDLPLL-----------------------------ILFRVVGLVTTVILFAI  147
A35            102  KAITLLTGLSMSAIATNMRVKGGGAYFLISRSLGPEFGGAIGLAFYLAQAVAVALYVVGFTESILSAFPDLPLLILEEITLVNGTAVNGTETAGADGEADSGDHDFRVVGLVTTVLLFAI  221
A25             67  SAVTLLTGISMSAIATNGRVKGGGAYYLISRSLGPEFGGAIGLCFYLANAVAVAMYVVGFTETILDLFPELGLLI-----------------------QDGGLHDLRVVGLVTCVLLFAI  163
A24             67  SAITLLTGLSMSAIATNGRVKGGGAYYLISRSLGPEFGGAIGLCFYLANAVAVAMYVVGFTETILDLFPDLGLLI-----------------------QDSGLHDLRVVGLVTTVLLFAI  163
A28            101  GACTFLTSISLSAIATNGAMKGGGPYYLIGRALGPEVGVSIGLCFFLGNAVAGSMYVLGAVETFLDAVPSAGLFQESVTVVNNTLV-NGTETAGTSTISTPSLHDLQVYGVIVTILLCFI  219
A27             89  GACTFLTSISLSAIATNGAMKGGGPYYLIGRALGPEVGVSIGLCFFLGNAVAGAMYVLGAVETFLDAVPSAGLFQESVTVVNNTAV--------AETISTPSLHDLQVYGVIVTILLCFI  200
A26             86  GACTLLTAISMSAIATNGAVKGGGSYYLISRSLGPEFGGAIGLCFYLGNAVAGAMYVLGAVETFLDAIPPAG-AIIQEE--------------AADGEASPALHDLRVYGIVTTVLLCLI  190

Conservation:          9     6 6   9  6  66     9 5  56                                                                  96 96 6  7 695   6
A32            166  SVAGMEWEAKAQIFLLVILIVAIANFFIGTFIPADTSK-----------------------------------------------------------KSKGFFGYDAEIFMENLGPDFR-  225
A34            172  SVAGMEWEAKAQIFLLVILITAIVNYFIGSFIAVDSK------------------------------------------------------------KPSGFFGYDAEILMENFGPDFR-  230
A33            172  SVAGMEWEAKAQIFLLVILITAIANFFIGTFIPVDSK------------------------------------------------------------KAKGFFGYDAEIFMENFGPDFR-  230
A31            167  VFVGTEWEAKAQIFLLVILLTAIVDFMIGSFLPPDDNK-----------------------------------------------------------KSRGFTGYDADILLENLWPDFR-  226
A29            167  VFIGTEWESKMQMFLLVILLVSIIDFMIGSFLPPDENK-----------------------------------------------------------EARGFTGYSADTLLENLWPDFR-  226
A30            167  VFIGTEFESKMQMGLLVILLVSIIDFMIGSFLPPDENK-----------------------------------------------------------ALRGFTGYSADTLVENLWPDFR-  226
A37            141  AYIGAGFAIRVQYVILAVLALSILSFFAGAPPEPGQIE---------------------------------------------------------------------------LLQDY--  183
A36            148  AYIGAGFAIRVQYVILAVLALSILSFFAGALPQPDELE---------------------------------------------------------------------------LLQGYN-  191
A35            222  VYIGAGWAIRVQYVILAVLLLSILSFFAGALAAPDELETLGITGLGNRTLSRRGFDVCAKVQAANNGSMATALWGLFCNSSTASTSCDQYFLQNNVTEIRGFPGYGAGTLLENLWQGYAG  341
A25            164  VFIGAEWANKLQLILLAVLLLSILSFFVGAFLPPDDAQ-----------------------------------------------------------ELRGFPGYAADTLLENLWQDYN-  223
A24            164  VYIGAGWAIKVQYIILAVLLLSILSFFAGALLPPDEAE-----------------------------------------------------------ALRGFPGYAAGTLLENLWQGYN-  223
A28            220  VFGGVKIINKVAPAFLIPVLFSLLCIYLGVFIAP-RHN-----------------------------------------------------------APKGITGLRLTSLKDNWGSNYQ-  278
A27            201  VFGGVKIINKVAPAFLVPVLLSLICIYLGVFIAP-RDN-----------------------------------------------------------PDKGITGLRLTSLKDNWGSEYQ-  259
A26            191  VFVGVKFVNKLALVFLACVLLSILAIYAGVFLAP-RDPPD------------------------------------------------------IGICGQGIPGLRAGTLLDNLWSGYQ-  254


Conservation:                                                       9 6666 69996999699 9 966979 6 699 9996969 6   97  6   66
A32            226  -------------------------------D--------------ETFFSVFAIFFPAATGILAGANISGDLKDPQDAIPKGTLLAILITGVVYLGVALSVGSCVVRDATGNINNTIGT  300
A34            231  -------------------------------D--------------ETFFSVFAIFFPAATGILAGANISGDLADPQMAIPKGTLLAILITGLVYLGVAISAGSCIVRDATGNLNNTIGT  305
A33            231  -------------------------------D--------------ETFFSVFAIFFPAATGILAGANISGDLADPQSAIPKGTLLAILITGLVYLGVAVSVGSCIVRDATGNINNTIGT  305
A31            227  -------------------------------D--------------ESFFSVFAVYFPAATGILAGANISGDLKDPQDAIPKGTLLAILITTIVYLGVAFMVGASVVRDANGVIPLTHHS  301
A29            227  -------------------------------D--------------ESFFSVFAVYFPAATGIMAGANISGDLKDPQDAIPRGTLLAILVTTVIYLGVVFMVGASVVRDADGVIPPTWNA  301
A30            227  -------------------------------G--------------ESFFSVFAVYFPAATGIMAGANISGDLKDPQDAIPRGTLLAILVTTVIYLGVVWMTGASCVRDADGIFPPAWNA  301
A37            184  --------------------------APSQPG--------------YNFWSVFAIFFPAVTGIMAGVNMSGDLKDPSRSIPRGTLLAILVSYVIYMALAFWLAAS-ANRQD---------  253
A36            192  ------------------------LSPSSQPG--------------YNFWSVFAIFFPAVTGIMAGVNMSGDLKDPSRSIPRGTLLAILVSYVIYLAIAFLLAAS-AQRQD---------  263
A35            342  KGAFTENKGLPSVAVPEDSGAGGLPSPNWQPG--------------YNFFSVFAIFFPAVTGIMAGVNMSGDLKDPSRSIPRGTLLAILVSAVIYLGIAFLLAASVAQRQDGLLPLALGA  447
A25            224  ------------------------LSPAGRPG--------------YSFFSVFAIYFPAVTGIMAGANMSGDLKDPQRSIPRGTLLAILVTTVIYLGVVLLLGAS-VQRAD---------  295
A24            224  ------------------------LSPASRPG--------------YNFFSVFAIYFPAVTGIMAGANMSGDLKDPQRSIPRGTLLAILVTTVIYLGVVFLLGAS-AQRQD---------  295
A28            279  -------------------------------RTNNAGVPDPSGSIYWDFNALVGLFFPAVTGIMAGSNRSASLKDTQRSIPIGTLSATLTTTAMYLLSVLLFGALATREEL---------  358
A27            260  -------------------------------RTNNAGVPDPSGSIYWSFNALVGLFFPAVTGIMAGSNRSASLKDTQRSIPIGTLSATLTTTAMYLLSVLLFGALATREEL---------  339
A26            255  -------------------------------DTENKGPPDPLGDIAWSFTALVGIYFPAVTGIMAGSNRSGDLKDAQRSIPLGTLLAILTTTFIYLACVLLFGACVERVVLRDKFGEL--  341

Conservation:                                               69                 669 6965 6996699 99 69966
A32            301  -------LLSNCT----------------------SCHY-GLMNN-QVM-SMVSGF--GPLITAGIFSATLSSALASLVSAPKV------------------------------------  350
A34            306  Q------LISNCTDAACKFGYDFSSCR-----STKSCRY-GLHNDFQVM-SLVSGF--SPLISAGIFSATLSSALASLVSAPKV------------------------------------  374
A33            306  -------LLSNCTDAACKFGYDFSSCR-------SSCHY-GLMNN-QVM-SLVSGF--SPLISAGIFSATLSSALASLVSAPKV------------------------------------  370
A31            302  GVQ----AIPNCTA------------------PNETCHY-GLMNNFQIM-ELVSWF--GPLITAGIFAATLSSALASLVSAPKV------------------------------------  359
A29            302  -------VVPDCA-------------------ANYTCPY-GLMNNFQIM-EMTSLW--GPLITAGIFAATLSSALASLVSAPKV------------------------------------  355
A30            302  TVF----VPPDCA-------------------ANYTCPY-GLMNYFQIM-EMTSLW--GPLITAGIFAATLSSALASLVSAPKV------------------------------------  358
A37            254  ----------------------------------------ELVEDPLVM-QRIARW--PPLIFAGVWAATLSSALGSLLGAPRV------------------------------------  294
A36            264  ----------------------------------------ELLEDPLVM-QKTALW--PPLIFAGVFAATLSSALGSLLGAPRV------------------------------------  304
a35            448  GVKDVVLLLPDCTGAACKLGYDFSSCRAAVLAANFACPYGELLEDPLVMLQKTALWGTPPLIFAGVFAATLSSALGSLLGAPRVFQVRGIFVVYLIAKGISHVITIIFKKTAVEYNDKDP  567
A25            296  ----------------------------------FSCPY-ELLNDPLVM-QKTALW--PPLIYAGIFAATLSSALASLVGAPRV------------------------------------  341
A24            296  ----------------------------------FACPY-ELLNDPLVM-QKTALW--PPLIFAGVFAATLSSALGSLLGAPRV------------------------------------  341
A28            359  -----------------------------------------LTDRLLTA-TVAWPA--PAVIYIGIILSTLGAALQSLTGAPRL------------------------------------  398
A27            340  -----------------------------------------LTDRLLTA-TVAWPA--PAVIYIGIILSTLGAALQSLTGAPRL------------------------------------  379
A26            342  -----------------------------------------LQDSLVTG-TVAWPS--PAVIYIGIFLSTLGAALQSLTGAPRL------------------------------------  381

Conservation:                                        66966 9 6 9  6  7  6 99   99  6  696 76 66 67                       6 96 9696966999  96
A32            351  ---------------------------------FQALCKDNIYPG-LQFFAKGYGKN-NEPLRGYILTFLIALAFILI----------------------AELNVIAPIISNFFLASYAL  413
A34            375  ---------------------------------FQALCKDNIYPG-IAIFAKGYGKN-NEPLRGYILTFGIALAFILI----------------------AELNVIAPIISNFFLASYAL  437
A33            371  ---------------------------------FQALCKDNIYPG-IQIFAKGYGKN-NEPLRGYILTFLIALAFILI----------------------AELNVIAPIISNFFLASYAL  433
A31            360  ---------------------------------FQALCKDNIFPG-IQYFAKGYGKN-NEPRRGYILTFLIALACILI----------------------GELNVIAPIISNFFLASYAL  422
A29            356  ---------------------------------FQALCKDNIFPG-IQYFAKGYGKN-NEPRRAYILTFLIALACILI----------------------GELNMIAPIISNFFLASYAL  418
A30            359  ---------------------------------FQAICKDNIFPK-IGYFAKGYGKN-NEPRRAYVLTFLIAMACILI----------------------GELNAIAPIISNFFLASYAL  421
A37            295  ---------------------------------LQALARDGVIPR-LRWLGRGSGKD-NEPRLALILTFILALACILL----------------------GDLDMIAPIITMFFLTTYGM  357
A36            305  ---------------------------------LQALARDGVFPR-LRWFGRGSGKE-NEPRRALVLTFLIALACILL----------------------GDLDMIAPIITMFFLTTYGM  367
A35            568  EIEDKPQQQLELQGELVASSHCESDEDLLAPNYLQALARDNVFPRRLRWFGRGSGKNKNEPRRALVLTFLIALACLLIGVLILNQCKRVEVVEQGGGDSLGDLDMIAPIITMFFLTTYGM  687
A25            342  ---------------------------------LQALARDNIFPR-LQYFGKGYGKN-NEPRRALILTFLIALACVLI----------------------GDLDMIAPIITMFFLASYAL  404
A24            342  ---------------------------------LQALARDNVFPRLLRWFGRGSGKN-NEPRRALILTFLIALACVLI----------------------GDLDMIAPIITMFFLTTYGL  405
A28            399  ---------------------------------LAAIANDDILPV-LNYFKVSEG---SEPHAATLFTAFICIGCVVI----------------------GNLDLITPTITMFFLLCYAG  459
A27            380  ---------------------------------LAAIANDDILPV-LNYFKVSEG---SEPHAATLFTAFICIGCVVI----------------------GNLDLITPTITMFFLLCYAG  440
A26            382  ---------------------------------LQAIARDDILPL-LQYFGHG--KANGEPRWALLLTALICIACILI----------------------GNLDLIAPIITMFFLMCYAF  443

Conservation:       69 6576  6   9669  9 6956666 99 97 69 659       9 7 5  5966  6
A32            414  INFSCFHASFAKSPGWR--PSFKYYNMWLSLAGAILCCGVM-------FVINWWAALLTYAIVLSLYVYVTYKKP---------------------------------------------  479
A34            438  INFSVFHASLANSPGWR--PSFKYYNMWASLAGSILCCGVM-------FIINWWAALLTNVI----------------------------------------------------------  490
A33            434  INFSVFHASLAKSPGWR--PSFKYYNMWASLAGSILCCGVM------NFVINWWAALLTYAI----------------------------------------------------------  487
A31            423  INFSCFHASFAKSPGFR--PSFKYYNMWISLAGAILCLGVM-------FIINWLAALLTFIIVLALYIYVQHRKPGKDWGSSCCA-----------------------------------  498
A29            419  INFSCFHASFAKSPGFR--PAFKYYNMWISLAGAILCIGVM-------FIISWLTALLTFLIVLALYLYIQHRKP---------------------------------------------  484
A30            422  INYSCFHASFAKSPGFR--PAFKYYNMWVSLAGALLCIAVM-------FIISWLTALLTFLIFLALYLYIQHRKPDVNWGSSTQA-----------------------------------  497
A37            358  INLAAGLEGFVENPSFR--PTFKV-HWSISLLGAVGCLAVM-------FLINPIAALVAIIIVAGIYLWLRRRELKARWGDVRSGLLYQLARRALLRLEREKPHP---------------  452
A36            368  INLAAFLEGFVRNPSFR--PTFKFYHWSVSLLGAVGCLAVM-------FLINPLAALVAIVIVAGIYLFLRRRELKARWGDVRSGLAYQLARRALLKLERERPHP---------------  463
A35            688  INLACFLEGFVRNPSFRWGPTFKFYHWSVSLLGAVGCLGVMDHVKLFSFLINPLAALVAIVLVAGIYFFIARRELKARWGDVRSGLAYQLARRALLKLERERPHPEKNWRPSILVLAGST  807
A25            405  INLACFLESFVKSPSFR--PTFKYYHWSISLLGAILCLGVM-------FLINWLAALVALLLVAALYLYIGRRKP---------------------------------------------  470
A24            406  INLACFLEGFVRNPSFR--PTFKYYHWSISLLGAVLCLGVM-------FLINWLAALVALLLVAALYLYIARRKLKARWGDARSGLAYQLARRALLKLERERPHPKNWRPSILVL-----  511
A28            460  VNLSCFLLDLLDAPSWR--PRWKYHHWSLSLVGALLCVATM-------FTMILNFD----------------------------------------------------------------  506
A27            441  VNLSCFLLDLLDAPSWR--PRWKYHHWSLSLVGALLCVAIM-------FLISWNFT----------------------------------------------------------------  487
A26            444  VNLACFLQSLLRTPSWR--PRFKYYHWSLSLVGALLCIAVM-------FLISWAFA----------------------------------------------------------------  490

Conservation:
A32                 --------------------------------------------------
A34                 --------------------------------------------------
A33                 --------------------------------------------------
A31                 --------------------------------------------------
A29                 --------------------------------------------------
A30                 --------------------------------------------------
A37                 --------------------------------------------------
A36                 --------------------------------------------------
A35            808  QTLTGSYRFHWALIEPAGAFSHGYGLLTVAAVLSDKHISNERKENMEETI  857
A25                 --------------------------------------------------
A24                 --------------------------------------------------
A28                 --------------------------------------------------
A27                 --------------------------------------------------
A26                 --------------------------------------------------
```
